# The effect of leisure time physical activity and sedentary behaviour on the health of workers with different occupational physical activity demands: a systematic review

**DOI:** 10.1186/s12966-021-01166-z

**Published:** 2021-07-20

**Authors:** Stephanie A. Prince, Charlotte Lund Rasmussen, Aviroop Biswas, Andreas Holtermann, Tarnbir Aulakh, Katherine Merucci, Pieter Coenen

**Affiliations:** 1 Centre for Surveillance and Applied Research, Public Health Agency of Canada, 785 Carling Avenue, Ottawa, Ontario K1A 0K9 Canada; 2 School of Epidemiology and Public Health, Faculty of Medicine, University of Ottawa, Ottawa, Canada; 3 National Research Centre for the Working Environment, Copenhagen, Denmark; 4 Institute for Work & Health, Toronto, Canada; 5 Dalla Lana School of Public Health, University of Toronto, Toronto, Canada; 6 School of Kinesiology and Health Studies, Queen’s University, Kingston, Canada; 7 Health Library, Health Canada, Ottawa, Canada; 8 Department of Public and Occupational Health, Amsterdam Public Health Research Institute, Amsterdam UMC, Vrije Universiteit Amsterdam, Amsterdam, the Netherlands

**Keywords:** Physical activity, Sedentary behaviour, Occupation, Leisure, Cardiovascular disease, Mortality

## Abstract

**Background:**

Although it is generally accepted that physical activity reduces the risk for chronic non-communicable disease and mortality, accumulating evidence suggests that occupational physical activity (OPA) may not confer the same health benefits as leisure time physical activity (LTPA). It is also unclear if workers in high OPA jobs benefit from LTPA the same way as those in sedentary jobs. Our objective was to determine whether LTPA and leisure time sedentary behaviour (LTSB) confer the same health effects across occupations with different levels of OPA.

**Methods:**

Searches were run in Medline, Embase, PsycINFO, ProQuest Public Health and Scopus from inception to June 9, 2020. Prospective or experimental studies which examined the effects of LTPA or LTSB on all-cause and cardiovascular mortality and cardiovascular disease, musculoskeletal pain, diabetes, metabolic syndrome, arrhythmias and depression among adult workers grouped by OPA (low OPA/sitters, standers, moderate OPA/intermittent movers, high OPA/heavy labourers) were eligible. Results were synthesized using narrative syntheses and harvest plots, and certainty of evidence assessed with GRADE.

**Results:**

The review includes 38 papers. Across all outcomes, except cardiovascular mortality, metabolic syndrome and atrial fibrillation, greater LTPA was consistently protective among low OPA, but conferred less protection among moderate and high OPA. For cardiovascular mortality and metabolic syndrome, higher levels of LTPA were generally associated with similar risk reductions among all OPA groups. Few studies examined effects in standers and none examined effects of LTSB across OPA groups.

**Conclusions:**

Evidence suggests that LTPA is beneficial for all workers, but with larger risk reductions among those with low compared to high OPA jobs. This suggests that, in our attempts to improve the health of workers through LTPA, tailored interventions for different occupational groups may be required. More high-quality studies are needed to establish recommended levels of LTPA/LTSB for different OPA groups.

**Protocol registration:**

PROSPERO #CRD42020191708.

**Supplementary Information:**

The online version contains supplementary material available at 10.1186/s12966-021-01166-z.

## Background

Physical activity and sedentary behaviour play key roles in the prevention of non-communicable chronic conditions and mortality [1–3]. Although generally accepted that greater physical activity reduces the risk for ill health, accumulating evidence suggests that occupational physical activity (OPA) may not confer the same health benefits as leisure time physical activity (LTPA) [4]. Higher levels of OPA have been associated with increased risk for work absence [5, 6], musculoskeletal disorders [7], high blood pressure [8], cardiovascular disease [9, 10], and all-cause mortality (among men) [11].

The postulated health sequelae of OPA are attributed to postures and movement patterns undertaken during occupational tasks (e.g., working while bending/twisting, kneeling, lifting, carrying/pulling heavy loads, and extensively walking or standing) [12], that are often performed for long periods of time with insufficient opportunities to rest. This is in contrast to LTPA, which is typically voluntary and performed in shorter bouts [13]. Furthermore, workers in jobs involving high OPA are generally found to perform less LTPA than workers in sedentary jobs [14], with a need to recover from the fatigue and pain of high OPA being commonly cited barriers to engaging in LTPA [15–17]. Because workers with high OPA often achieve the physical activity guideline of ≥ 150 min/week of moderate-to-vigorous intensity physical activity [18] at work alone, it is important to investigate if they receive the same benefit of LTPA as workers in sedentary jobs.

The primary objective of this systematic review was to determine whether LTPA confers the same health benefits among workers with different levels of OPA. The secondary objective was to examine the health effects of leisure time sedentary behaviour (LTSB) across OPA levels.

## Methods

This review was prospectively registered with PROSPERO (CRD42020191708) and documented on Open Science Framework (https://osf.io/5angw/), and adheres to the PRISMA statement [19].

### Study inclusion criteria

#### Participants

Adult workers (mean age: 18–65 years) with adequate information on OPA, who were generally healthy and did not report the outcomes of interest at baseline (e.g., pre-existing musculoskeletal pain, depression), except if baseline values were controlled for in the analyses.

#### Exposures

The primary exposure was LTPA. Physical activity is defined as “any bodily movement produced by skeletal muscles that results in energy expenditure” [20] > 1.5 metabolic equivalents and can include time spent in light, moderate and vigorous intensity [21]. LTPA specifically refers to physical activity during free time (i.e. outside work hours; e.g. recreational-, travel- and household-related physical activity), and is based on personal interests and needs (e.g., walking, gardening, sports, exercising, dancing) [20, 22]. LTPA can be measured via self-report (e.g., questionnaire, diary/log) or by device (e.g., accelerometers in combination with self-reported work or leisure time to differentiate OPA from LTPA).

To meet the second objective studies were required to provide a quantification of LTSB. Sedentary behaviour includes non-sleeping activities undertaken at ≤ 1.5 metabolic equivalents while sitting, lying or reclining [23]. Also, a measure to identify whether sedentary behaviour occurred outside of work hours was needed. LTSB could include total leisure time spent sedentary, sitting, or in a specific sedentary behaviour (e.g., watching television, reading, using an electronic device).

#### Outcomes

All-cause and cardiovascular mortality, diabetes, metabolic syndrome, arrhythmias, cardiovascular disease, musculoskeletal pain, and depression.

#### Study designs

Intervention/experimental, retrospective or prospective cohort studies, and case-control studies. Cross-sectional studies were excluded as they were unable to assess some level of causation.

#### Publication status and language

Publications in English, French, Danish, Norwegian, or Dutch were eligible based on authors’ language capacity. Published studies were eligible provided they included sufficient information. All literature, regardless of date of publication, was considered.

### Search strategy

The search strategy was created by a research librarian (KM) in collaboration with other members from the authorship team. The search was first created in Medline using a combination of index terms/unique subject headings and keywords related to occupations, physical activity, sedentary behaviour, study designs, and adult populations. After trial searches, search strategies were modified to ensure that pre-identified key papers were captured. Once the strategy was finalized (Supplemental Table 1), the following electronic bibliographic databases were searched: Ovid Medline(R) All; Ovid Embase; Ovid APA PsycINFO; ProQuest Public Health; and, Scopus (from database inception to June 9, 2020).

### Selection of studies

After removing duplicates, two independent reviewers screened titles and abstracts to identify potentially relevant articles using Covidence. Full texts of articles that either met the inclusion criteria or provided insufficient information in the abstract were obtained. Two independent reviewers screened the full texts for inclusion. Discussion between the reviewers and a possible third reviewer was conducted to resolve potential conflicts. Reviewers were not blinded to the authors of the studies, but they did not screen or extract data from any of their own papers.

### Risk of bias

The risk of bias of studies was assessed using a standard (interventional studies) [24] and modified version (observational studies) [25] of the *Cochrane Collaboration’s Tool for Assessing Risk of Bias* for consistent comparisons across studies of different designs. Potential biases included selection bias, performance bias, detection bias, attrition bias, selective reporting bias and, other possible sources of bias. Risk of bias assessments were carried out by one independent reviewer and verified by a second, and were summarized using summary graphs [26]. Although the tool was not designed to provide a score, to provide some judgement regarding the overall quality of each study, studies with ≤ 1 biases (high/unclear) were rated as ‘high quality’, 2–4 as ‘moderate quality’ and ≥ 5 as ‘low quality’.

### Data extraction and analysis

Classification of OPA was based on the study’s description of physical job demands, job title and/or self-reported or device-based OPA. OPA for each study was assigned by one independent reviewer and, once completed; a second reviewer verified for accuracy. Workers were grouped, where possible, based on their most frequent occupational tasks and OPA into the following four groups: (1) **low OPA/sitters**: tasks largely conducted while sitting with little OPA; (2) **standers**: tasks largely conducted while stationary standing; (3) **moderate OPA/intermittent movers**: tasks largely involving frequent postural changes (e.g. sitting to standing) with low-intensity OPA and without heavy laborious tasks; and, (4) **high OPA/heavy labourers**: tasks involving moderate-to-high intensity OPA and may include carrying/lifting/pushing/pulling heavy loads, and extensive walking. From henceforth, we refer to the four groups as low OPA, standers, moderate OPA, and high OPA.

Standardized data extraction forms were developed and completed using Google Forms. One reviewer independently extracted data and a second independent reviewer verified the accuracy of the extractions. Effect estimates (e.g., odds ratio, relative risk, hazards ratio) for the association between LTPA or LTSB and each outcome were extracted and included results from fully adjusted models. A narrative synthesis summarizes and describes trends in risk associated with lower compared to higher quantities of LTPA across all outcomes within each of the OPA groups. Although a meta-analysis was initially planned in the protocol, it was precluded due to much heterogeneity between studies resulting from differences in populations, measures of LTPA and OPA, and reference and comparison categories used in analyses. Harvest plots display the data visually in terms of risk (higher/lower/no risk) associated with LTPA among the OPA groups, and are based on statistical significance (*p* < 0.05) where possible [27]. Within each outcome, findings from all study designs were combined. For studies where no direct statistical tests were conducted, a visual trend of patterns across effect estimates of indirect comparisons or summaries from article text were used to determine direction of association (and denoted in the harvest plots). Studies that did not reach statistical significance, but had clinically relevant findings (i.e. ≥ 10% higher or lower risk/odds) were placed under ‘null’, but identified as trending. Risk of bias assessments were incorporated into the harvest plots by lowering the study bar height by one unit for each of the six biases that were identified as unclear/high risk. When multiple articles described the same study, only one article per study and outcome was included in the harvest plots. We chose the one that provided the most direct comparison across LTPA and OPA groups, had the greatest sample size or was the most recent (in this order). Additional articles on that same dataset were retained and presented in the summary of findings table if findings were presented differently (i.e. different reference/comparison groups) or if they reported on different outcomes. A priori identified subgroup analyses included an examination of results by sex, publication year and risk of bias. In contrast to our protocol, we were unable to compare self-report vs. device-based LTPA as all studies relied on self-reported LTPA.

The certainty of the evidence within each health outcome was assessed as high, moderate, low or very low using a modified Grading of Recommendations Assessment, Development and Evaluation (GRADE) approach adapted for narrative syntheses [28, 29]. The GRADE approach assesses certainty of evidence based on study design, possible risk of bias, indirectness, imprecision, inconsistency, and suspicion of publication bias. Within this approach, for the musculoskeletal pain and depression outcomes, randomized controlled trials began as high-quality and non-randomized studies began as low-quality evidence and then graded down depending on aforementioned factors. In case of other more distal disease outcomes (i.e., diabetes, arrhythmias, metabolic syndrome, cardiovascular disease, mortality) where experimental designs would be harder to achieve, prospective cohort studies began as high-quality evidence.

## Results

### Study characteristics

**Fig. 1:**
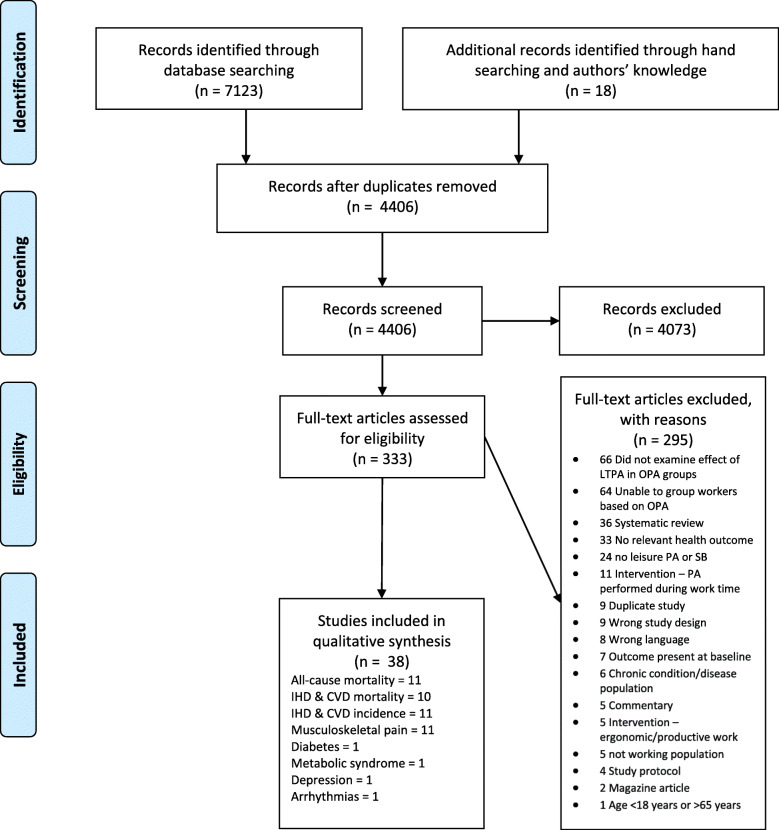
PRISMA flow diagram

The search of electronic databases identified 7123 potentially relevant papers (Fig. 1). Of these, 2495 were identified in Medline, 2392 in Embase, 731 in PsycINFO, 305 in ProQuest Public Health, and 1200 in Scopus. Author’s knowledge and bibliographies identified 18 additional papers. Title and abstract review resulted in the retrieval of 333 full text papers for assessment. Of these, 38 papers representing 34 independent studies met the eligibility criteria [10, 30–66]. A list of excluded full texts and reasons is found in Supplemental Table 2. Individual study characteristics and findings for LTPA and LTSB are shown in Supplemental Tables 3–6. Table 1 presents the summary of findings and the certainty of evidence.

**Table 1:**
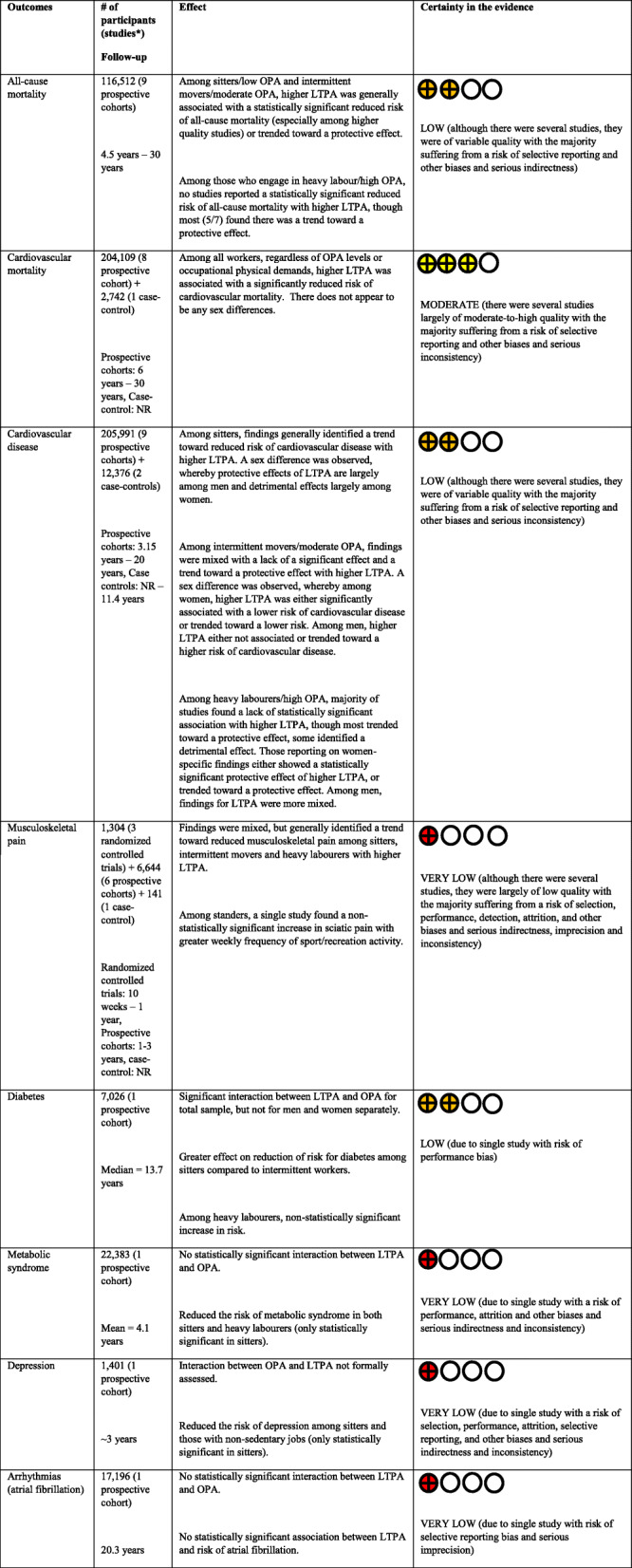
Summary of findings table for effect of high vs. low LTPA on health outcomes across occupational physical demand groups.

*LTPA* Leisure time physical activity, *NR* not reported, *OPA* occupational physical activity.

Included papers were published between 1981 and 2020 with the majority (61%) from the past 10 years. Fifteen countries were represented, with most papers reporting on data from Finland (26%) and Denmark (26%); two studies [33, 64] captured data from North America. The majority of studies included both men and women, with eight including only men, and five including only women. All studies used self-report methods to ascertain LTPA and OPA. Only one study examined LTSB [55]. Low OPA occupations were the most studied. Only three studies [30, 36, 56] had workers classified in the standers group. Only one study per outcome was identified for diabetes [33], metabolic syndrome [52], depression [32], and arrhythmias [60]. Musculoskeletal pain included general pain, and pain in specific locations such as in the neck, lower back, shoulders, and upper limbs. Cardiovascular disease included ischemic heart disease, myocardial infarction, stroke, and heart failure. Prospective cohort studies were the most common study design, except for musculoskeletal pain, which was assessed with a mix of randomized controlled trials and cohorts.

### Risk of bias

Risk of bias is summarized in Supplemental Fig. 1 (randomized controlled trials and observational studies separated) and captured in the harvest plots. Among randomized controlled trials, the majority (75%) had a low risk of performance and selective reporting bias. Most (75%) had a high or unclear risk of selection bias due to poor allocation concealment. Loss to follow-up was an issue in half of the studies.

Among cohort and case-control studies, 20% had a high risk of selection bias, largely due to convenience samples limiting generalizability of findings; for another 20% the risk of selection bias was unclear. Approximately 30% had a high risk of performance bias due to the use of self-reported LTPA measures that were non-validated, an additional 25% were unclear. The majority (80%) had a low risk of detection bias as most outcomes were assessed objectively using medical records and registries. Most had a low risk of attrition bias with < 20% loss to follow-up. For half of the papers, it was unclear if selective reporting bias was present due to a lack of clarity on whether all available data were reported. More than half of the papers had a high risk of ‘other’ biases largely attributed to a lack of adjustment for the potential for reverse causality.

### All-cause mortality

**Fig. 2:**
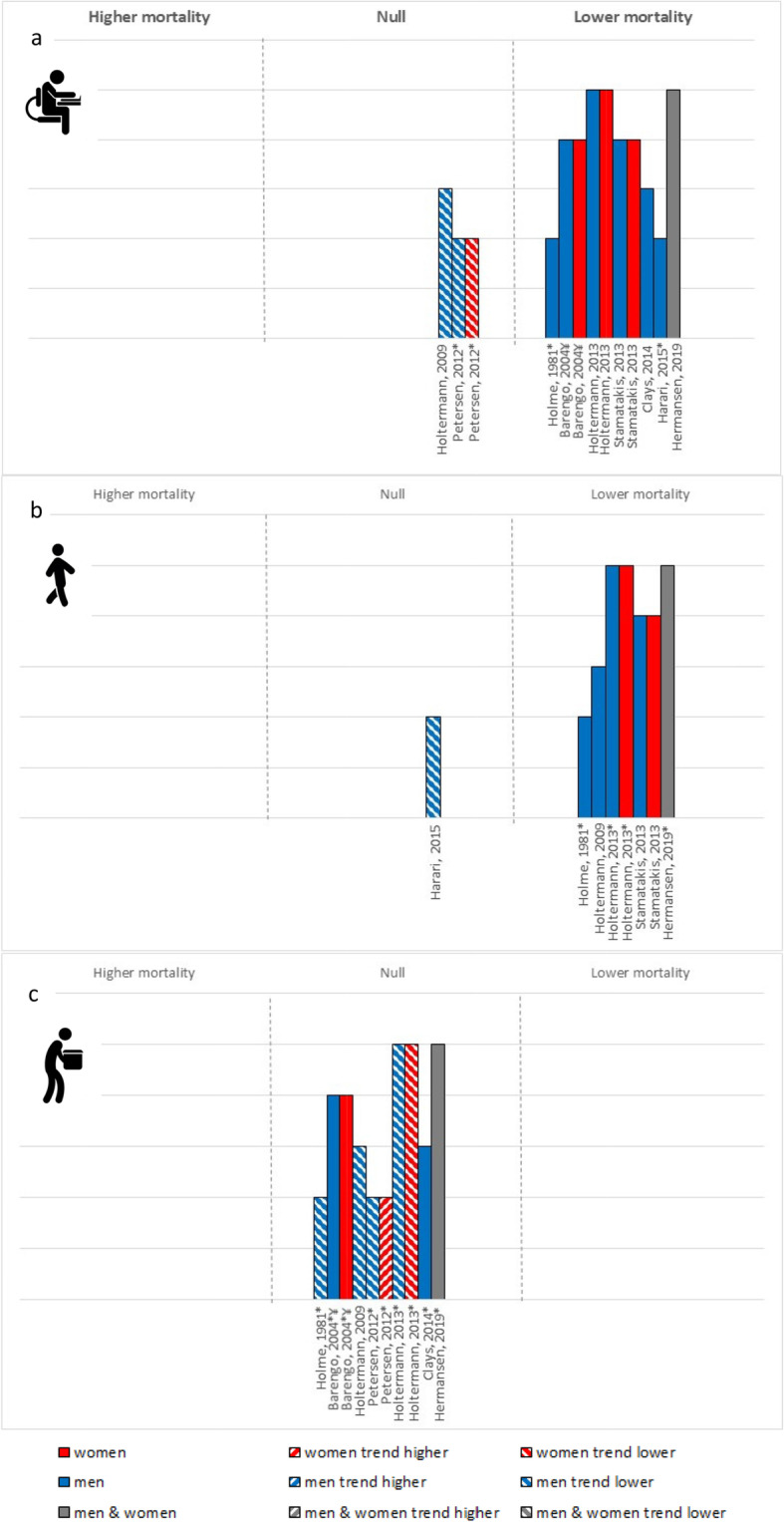
All-cause mortality risk associated with high vs. low LTPA among (**a**) low OPA group, (**b**) moderate OPA and (**c**) high OPA. Each bar represents a study/analysis. The height of each bar indicates the study quality; with higher bars assessed as higher quality with fewer biases. Bars are arranged by publication date moving from oldest to newest. * – Not based on formal statistical testing, but visual trends in the data. ¥ – data compared low LTPA to mod-high LTPA

Eleven papers (9 studies) of variable quality, examined the interaction between OPA and LTPA and all-cause mortality using prospective cohorts (Fig. 2). Among low OPA and moderate OPA, high vs. low LTPA was generally associated with a statistically significant reduction in all-cause mortality risk (especially among higher quality studies) or trended toward a protective effect. Among those who engage in high OPA, no studies reported a statistically significant reduced risk of all-cause mortality for high vs. low LTPA, though most (5/7 studies) found a trend toward a protective effect. The overall certainty in the evidence for all-cause mortality was low.

One study examined the interactive effects of LTSB and OPA on all-cause mortality. This study only included low OPA; therefore, comparisons across OPA groups could not be made.

### Cardiovascular mortality

**Fig. 3:**
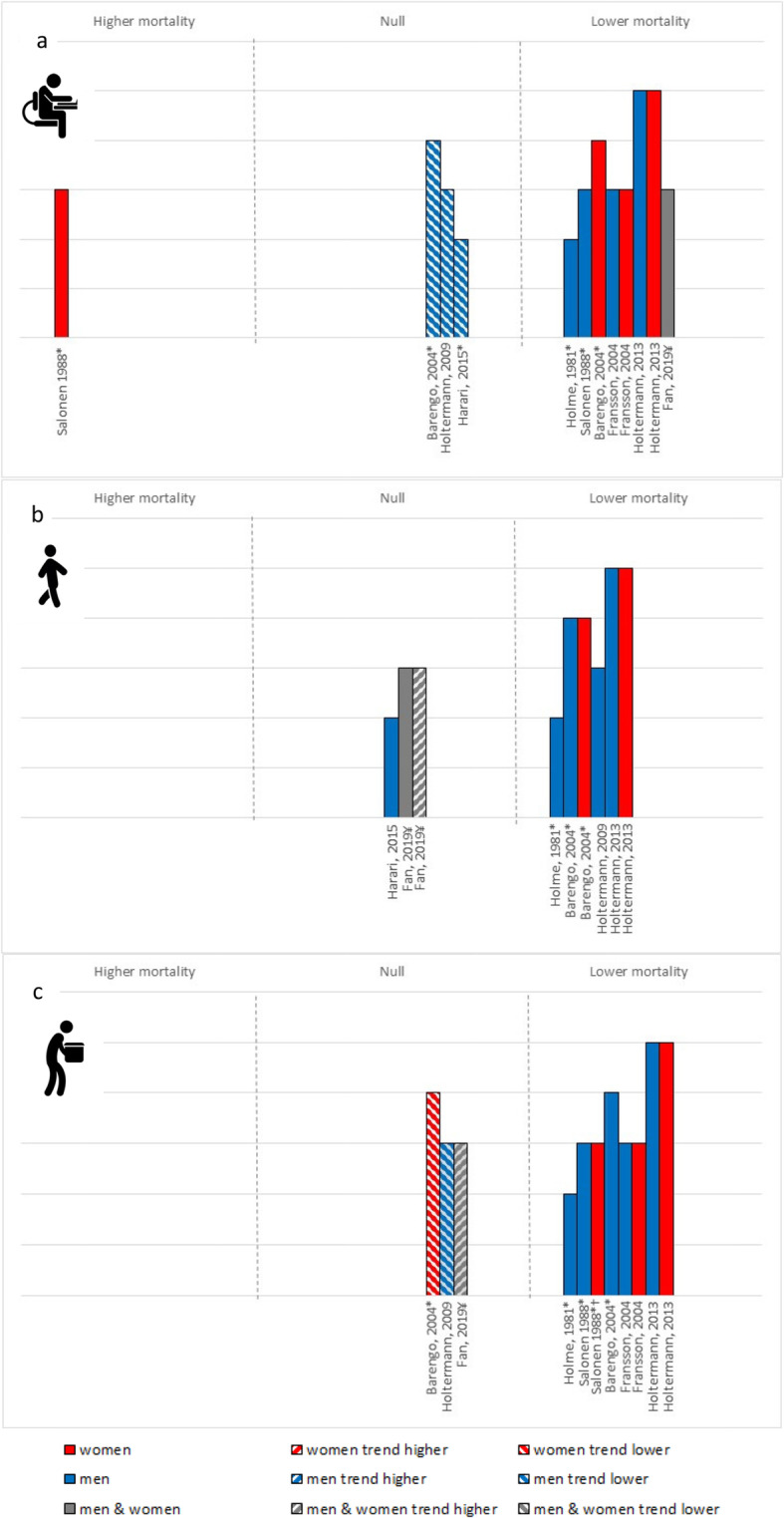
Cardiovascular mortality risk associated with the high vs. low LTPA among (**a**) low OPA, (**b**) moderate OPA and (**c**) high OPA. Each bar represents a study/analysis. The height of each bar indicates the study quality; with higher bars assessed as higher quality with fewer biases. Bars are arranged by publication date moving from oldest to newest. * – Not based on formal statistical testing, but visual trends in the data. ¥ – Findings for (**a**) and (**c**) are for both coronary heart disease and cardiovascular disease events, for (**b**) coronary heart disease is trending and cardiovascular disease events are not associated. †Among women in active occupations, findings showed protective effects among those with low body mass index, but detrimental effects among those with high body mass index

Ten papers (7 studies; 9 cohort, 1 case control) of moderate-to-high quality examined the interaction between OPA and LTPA and the risk of cardiovascular mortality (Fig. 3). Regardless of OPA, greater LTPA was generally associated with a statistically significant reduced risk of cardiovascular mortality. There were no clear sex differences in the association between LTPA and cardiovascular mortality among workers by OPA, nor any patterns by publication date. The overall certainty in the evidence for cardiovascular mortality was moderate.

### Cardiovascular disease

**Fig. 4:**
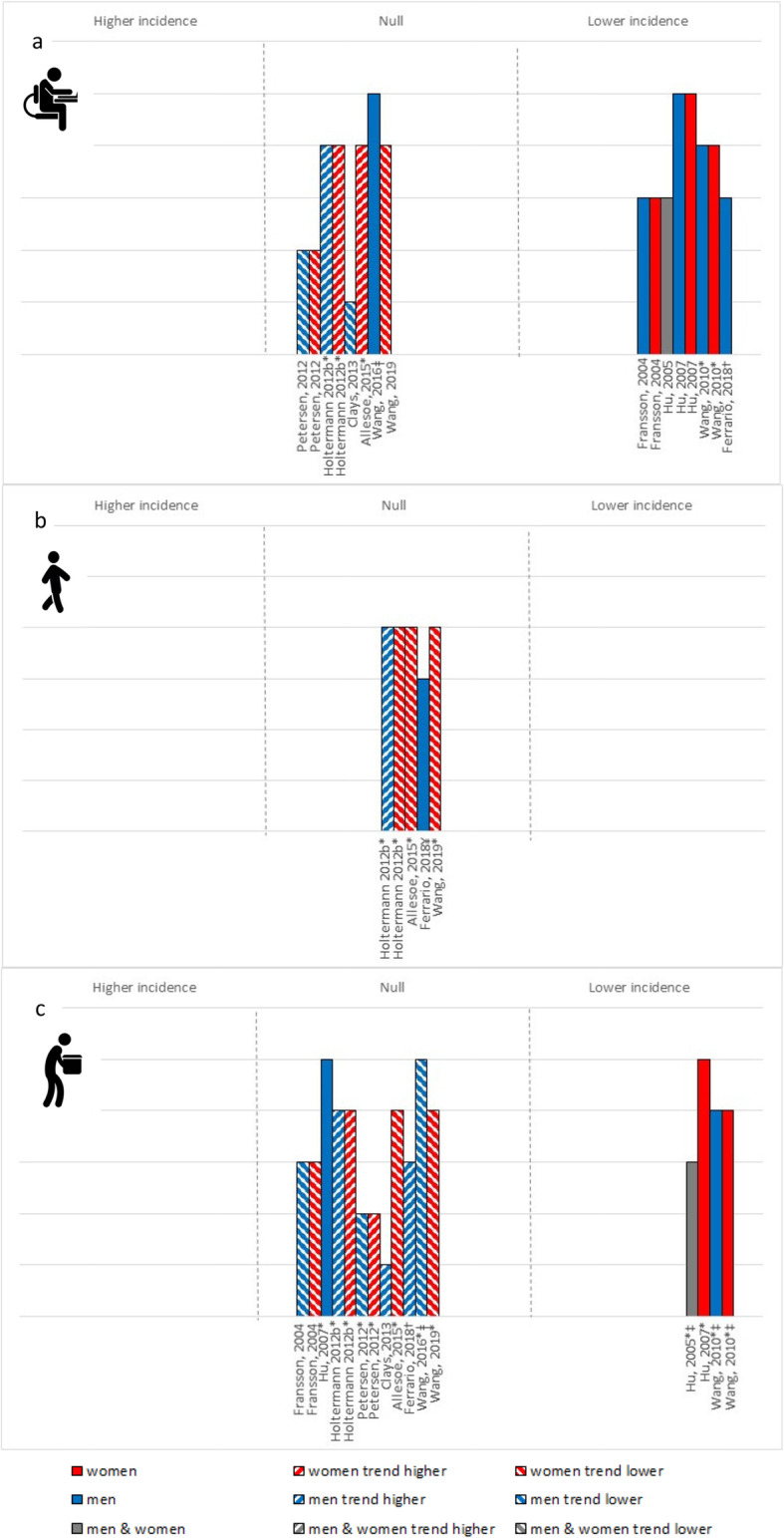
Cardiovascular incidence risk associated with high vs. low LTPA among (**a**) low OPA (**b**) moderate OPA and (**c**) high OPA. Each bar represents a study/analysis. The height of each bar indicates the study quality; with higher bars assessed as higher quality with fewer biases. Bars are arranged by publication date moving from oldest to newest. * – Not based on formal statistical testing, but visual trends of data. ¥ – Trend of increasing risk for coronary heart disease and decreasing for cardiovascular disease. † − Findings are for both coronary heart disease and cardiovascular disease events. ‡ − Includes moderate and active OPA group (greater % active vs. moderate among men, and ~ equal distribution among women). ǂ – Findings are for men without ischemic heart disease

Eleven papers (10 studies; 9 cohorts, 2 case-control), examined the interaction between OPA and LTPA and cardiovascular disease (Fig. 4). All studies examined the effects of LTPA among low OPA workers and were largely of moderate-to-high quality. Of the 11 papers, five reported a statistically significant reduced risk of cardiovascular disease with higher vs. lower amounts of LTPA; they included heart failure and stroke outcomes. Six studies examined a coronary heart disease endpoint and found no statistically significant effects, but half trended toward a protective effect, while the others trended toward a detrimental effect. Four papers examined the effects among moderate OPA and found no statistically significant effect of LTPA. In this group of workers, a sex difference was observed. Among women, high vs. low LTPA always trended toward a lower risk. Among men, there was either no association or a trend toward a higher risk. All eleven papers also examined the effects of LTPA among high OPA workers. While most (75%) found a lack of statistical association, four found it associated with lower cardiovascular disease risk (3/4 included heart failure and stroke outcomes). Those reporting on women-specific findings either showed a statistically significant protective effect of high compared to low LTPA, or trended toward a protective effect. Among men, findings were more mixed. The overall certainty in the evidence for cardiovascular disease was low.

### Musculoskeletal pain

**Fig. 5:**
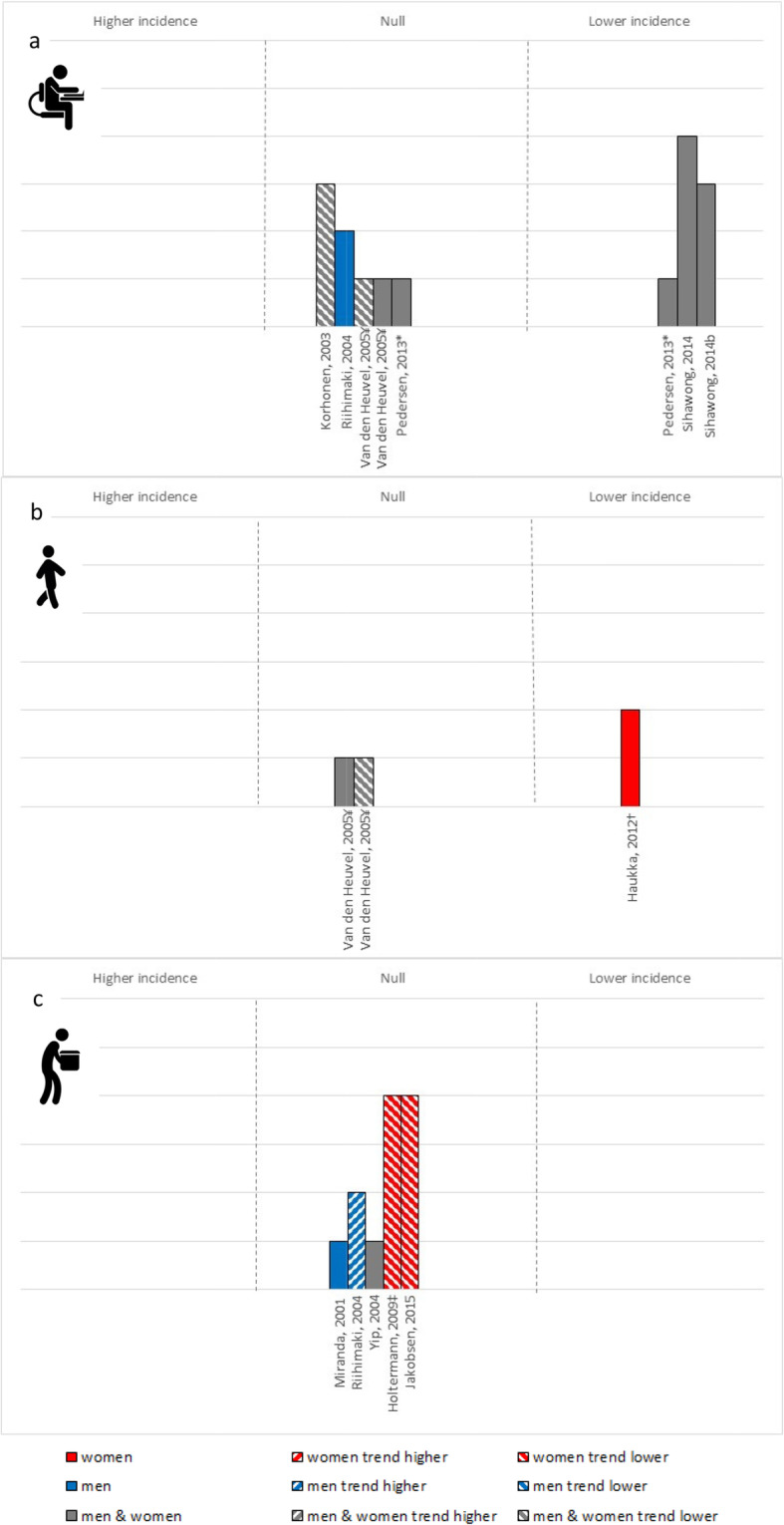
Musculoskeletal pain risk associated with high vs. low LTPA among (**a**) low OPA, (**b**) moderate OPA and (**c**) high OPA. Each bar represents a study/analysis. The height of each bar indicates the study quality; with higher bars assessed as higher quality with fewer biases. Bars are arranged by publication date moving from oldest to newest. * – Null findings are for left shoulder, elbow or hand pain, whereas there were significant intervention effects for neck pain, right shoulder pain and right hand pain. ¥ – Null findings for both neck-shoulder symptoms, as well as elbow/wrist/hand symptoms using sport frequency per week. † − Findings based on risk of high vs. low trajectory for musculoskeletal pain. ‡ − Data presented for the home-based exercise intervention arm

Eleven papers (11 studies; 4 randomized controlled trials, 6 cohorts, 1 case-control) examined the interaction between OPA and LTPA and the risk of musculoskeletal pain (Fig. 5). Six papers (5 studies) of low-to-moderate quality explored the effects of LTPA among low OPA workers. Evidence was mixed, but trended toward a reduced risk of musculoskeletal pain across multiple pain sites with high compared to low levels of LTPA. Two studies [40, 62] with a high risk of bias examined the effects of LTPA among moderate OPA. One found that the odds of a high vs. low trajectory of musculoskeletal pain was greater with low compared to high LTPA (OR = 2.3, 95% CI: 1.1–4.7) [40]. The other found a trend toward lower elbow/wrist/hand symptoms, but no significant effect on neck/shoulder pain with weekly frequency of sport and recreation [62]. Five studies of varying quality found no statistical evidence of a protective effect of higher vs. lower volumes of LTPA among workers with high OPA. Two higher quality studies [43, 50] reported on effects among women and found a trend toward a protect effect for global musculoskeletal pain. Two lower quality studies among men found either no association with shoulder pain [53] or a trend toward higher incidence of sciatic pain [56] with greater LTPA. Only one study explored effects of LTPA among standers, and found that greater LTPA was associated with a non-statistically significant increase in sciatic pain among male machine operators (RR = 1.24, 95% CI: 0.86–1.81) [56]. There were no clear trends by publication date or study design. The overall certainty in the evidence for musculoskeletal pain was very low.

### Diabetes

One high quality cohort study assessed the association between LTPA and OPA for diabetes risk [33]. A significant interaction was found between LTPA and OPA in the overall sample, but was not observed among men and women separately. Higher LTPA had a significant effect on reducing diabetes risk for workers with low OPA (HR = 0.63, 95% CI: 0.47–0.85), but had a weaker and non-statistical association among those with moderate OPA (HR = 0.92, 95% CI: 0.55–1.55). Those with high OPA even had an increase (though not statistically significant) in diabetes risk with high compared to low levels of LTPA (HR = 1.07, 95% CI: 0.73–1.56). The overall certainty in the evidence for diabetes was low.

### Metabolic syndrome

One moderate quality cohort study assessed the interaction between LTPA and OPA and the risk of metabolic syndrome [52]. No statistically significant interaction between OPA and LTPA was found. Higher LTPA was associated with a similar reduced risk of metabolic syndrome in the low and high OPA groups, though was only statistically significant in the low OPA group. The overall certainty in the evidence for metabolic syndrome was very low.

### Depression

One low quality cohort study examined the risk of depression [32]. Among low OPA, compared to those with low levels of LTPA (less than once per month), higher LTPA was associated with a statistically significant reduced likelihood of approximately 40% for future depression (OR = 0.62, 95% CI: 0.43–0.91). While not statistically significant, those with non-sedentary jobs observed a reduction of 20% in the odds of future depression with higher compared to lower LTPA (OR = 0.80, 95% CI: 0.53–1.21). The overall certainty in the evidence for depression was very low.

## Arrhythmias

One high quality cohort study examined the risk of arrhythmias; specifically atrial fibrillation [60]. There was no statistically significant interaction between OPA and LTPA. The overall certainty in the evidence for atrial fibrillation was very low.

## Discussion

### Main findings and comparisons with the literature

This systematic review offers insight into whether LTPA confers the same health benefits for workers with different OPA levels. Across various health outcomes (i.e., all-cause mortality, cardiovascular disease, musculoskeletal pain, diabetes, and depression), greater LTPA was consistently protective among low OPA, but conferred less protection among moderate and high OPA. However, no clear statements can be drawn due to the relatively few studies and low-to-very low certainty of evidence. The most consistent evidence was found for cardiovascular mortality and metabolic syndrome, showing a protective effect of LTPA across the OPA groups. No evidence was available regarding health risks from LTSB across OPA groups. These findings support the need for more studies investigating the effects of LTPA and LTSB on health risk among workers with different OPA levels. Moreover, while there is little doubt that LTPA is beneficial for workers in sedentary jobs, we still lack high quality evidence to give recommendations of LTPA among workers in jobs involving moderate and high OPA levels or standing.

Previous systematic review findings complement those observed in the present review, suggesting that LTPA confers a stronger independent protective effect compared to OPA for type 2 diabetes [67], cardiovascular disease [68] and all-cause mortality [69]. In fact, evidence suggests that high OPA may have a detrimental effect on all-cause mortality independent of LTPA [70]. An overview of reviews on OPA and health [71] found that high vs. low OPA was associated with a greater risk for all-cause mortality among men, mental ill health (i.e., depression, anxiety), osteoarthritis, and sleep quality and duration, although there was also positive health benefits, including reduced risk of colon and prostate cancer, ischemic stroke, and coronary heart disease. Additionally, the authors found a greater risk reduction for coronary heart disease, distal colon cancer and type 2 diabetes with higher levels of LTPA among workers with low OPA compared to workers with high OPA [71]. It is possible that these findings can be attributed to reduced levels of LTPA among workers with high OPA [14, 72]. However, among the studies examining cardiovascular disease, and cardiovascular and all-cause mortality and reported LTPA levels across OPA groups, most found that higher OPA groups either showed higher or similar levels of LTPA compared to lower OPA groups [10, 30, 39, 42, 44, 45, 47]. Additionally, among studies that reported on the effects of OPA after controlling for LTPA, there was no clear pattern as to whether there was an independent increased or decreased risk of cardiovascular disease and cardiovascular or all-cause mortality associated with high compared to low OPA. A 24-h approach to assessing the contributions of LTPA and OPA might better elucidate their contributions to health.

There appeared to be differences between men and women on the effects of LTPA on cardiovascular disease, especially among high OPA. Differences may be attributed to a mix of sex and gender-specific considerations such as: differences in the tasks or occupational exposures of men and women in the same jobs/workplaces; effects and exposure to toxic workplace substances; hormonal and other factors implicated in the development of cardiovascular disease; employment status and hours worked; interactions between tasks undertaken at work and home; or, high OPA representing different types of occupations with different types of demands for women [73]. It is also possible that our findings are partially driven by a lower power to detect effects among women as most combined male/female samples had fewer women than men in the high compared to low OPA group. A systematic review and meta-analysis by Coenen et al. found that among men, compared to those with low OPA, those with high OPA were at an 18% increased risk for all-cause mortality (HR = 1.18, 95% CI: 1.05–1.34). However, this effect was not observed among women (HR = 0.90, 95% CI: 0.80–1.01) [74]. Given the potential for sex and gender differences, future studies should report results for men and women separately.

### Implications for research

Physical inactivity is among the most recognized modifiable risk factors for the maintenance of good health and longevity [3]. Recently, the World Health Organization launched the new Physical Activity and Sedentary Behaviour Guidelines [75] that recommend adults undertake 150–300 min/week of moderate-to-vigorous intensity physical activity or 75–150 min/week of vigorous intensity physical activity. Due to insufficient evidence to determine whether the health benefits of physical activity vary by type or domain, the guidelines suggest that physical activity at work, leisure, home and during transportation all contribute equally to the recommended amounts [75]. Although some of the first studies on physical activity and health explored the effects of OPA (e.g., London bus driver studies, San Francisco longshoremen [76, 77]), much of the contemporary literature has focused on LTPA [3]. The potential differential health effects of OPA and LTPA, which has been referred to as the “physical activity health paradox”, have challenged the assumption that all physical activity is healthful, suggesting that higher levels of OPA increase the risk of adverse health outcomes in contrast to the consistent benefits associated with LTPA [4, 13, 78]. With most workers spending at least half of their day at work, job demands play an important role in the level of OPA, but also on the time, resources and ability to engage in LTPA. There remains a need for high quality studies, using valid and reliable measures of physical activity, to disentangle whether the domain of physical activity matters for achieving the physical activity guidelines, and to what extent people with different levels of physical demands at work benefit from additionally engaging in LTPA.

We found very few studies examined the health effects of LTPA on workers with prolonged occupational standing. Prolonged occupational standing has been associated with muscle and psychological fatigue [79, 80], low back pain [81], and vascular symptoms and disease [80, 82]. Future work is needed to explore health outcomes among workers who spend a large portion of their day standing, and the role of LTPA in this group. Additionally, there is a need for studies examining whether the risks of LTSB are consistent across all OPA groups.

### Implications for practice

Our findings support that LTPA is essential to the overall health of workers. One of the ways in which physical activity promotes better health is through its ability to improve cardiorespiratory fitness [83]. While LTPA is known to increase cardiorespiratory fitness [84], there is evidence to suggest that higher OPA does not necessarily confer the same benefits [85–88]. The combination of high OPA and low cardiorespiratory fitness (and thus a worker not fit for the physical job demands) may pose a greater risk for mortality compared to individuals with high OPA/high cardiorespiratory fitness [89, 90]. Therefore, improving the fitness of workers via regular LTPA in order to facilitate a reduced cardiovascular load when performing occupational tasks is likely an important strategy for improving worker health [91, 92]. Workplace-based physical activity interventions have been shown effective for increasing cardiorespiratory fitness, also among workers with high OPA [93]. Workplace design including access to showers and change rooms and bike facilities have been shown to be associated with active commuting among workers [94, 95], which is in turn associated with higher cardiorespiratory fitness [96, 97]. Workplace interventions, may therefore, benefit from including built environment changes that support active commuting to work, but also organizational changes that support workers to be physically active during leisure. An additional strategy to promote improved health and fitness could be to redesign and reorganize the content of jobs characterized by high OPA [98].

### Strengths and limitations

The strengths of this review include a very rigorous approach, an a priori protocol, a comprehensive and peer-reviewed search strategy, the use of harvest plots that considered both statistical and clinical significance, and the use of GRADE to assess the certainty of evidence. The review was, however, limited to studies where occupations could be categorized into one of the predefined OPA groups. Secondly, the measures of OPA and LTPA were all self-reported and varied widely. This led to a large amount of heterogeneity in the OPA and LTPA groups and likely misclassified physical activity intensity. Thirdly, while we did include data from fully adjusted models, it is possible that residual confounding remains, either because of misclassification within considered confounders, or from other factors that were not considered. Although our inclusion criteria required a longitudinal or experimental design to assess some degree of causality, the assessment of OPA and LTPA at one point in time may not accurately capture lifetime exposure. We attempted to reduce this bias by including a requirement for the analysis to adjust for reverse causality by removing at least the first 2 years of follow-up data. Unfortunately, most prospective studies did not undertake this approach. Lastly, most of the literature has come from Scandinavian and Western European countries. A recent study explored domain-specific physical activity and found a wide variation in levels of LTPA and OPA across countries [99]; it is possible that these findings are not generalizable to workforces in other countries/settings.

## Conclusions

Low certainty evidence from this review suggests that LTPA is overall beneficial for all workers, though the magnitude of benefit may depend on OPA levels. Across most health outcomes (i.e., all-cause mortality, cardiovascular disease, musculoskeletal pain, diabetes, and depression), greater LTPA was consistently protective among low OPA, but conferred less consistent protection among moderate and high OPA. For cardiovascular mortality and metabolic syndrome, we generally found more consistent evidence for a protective effect of LTPA across all OPA groups. These findings suggest that LTPA interventions may need to be tailored based on the OPA level of workers. We still lack high quality evidence to provide specific LTPA recommendations for workers with different types of occupational physical demands (e.g., higher level OPA or large volumes of stationary standing).

## Supplementary Information

**Additional file 1.**

## Data Availability

All data generated or analysed during this study are included in this published article [and its additional files].

## Abbreviations

GRADE: Grading of recommendations assessment, development and evaluation
HR: Hazards ratio
LTPA: Leisure time physical activity
LTSB: Leisure time sedentary behavior
OPA: Occupational physical activity
OR: Odds ratio
RR: Relative risk
