# Supplementary material for: The effect of leisure time physical activity and sedentary behaviour on the health of workers with different occupational physical activity demands: a systematic review
Source: Int J Behav Nutr Phys Act. 2021 Jul 20;18:100. doi: 10.1186/s12966-021-01166-z (PMC8290554; doi:10.1186/s12966-021-01166-z)
Supplement: Supplementary file 1 — Additional file 1. [file 12966_2021_1166_MOESM1_ESM.pdf]

Randomized controlled trials

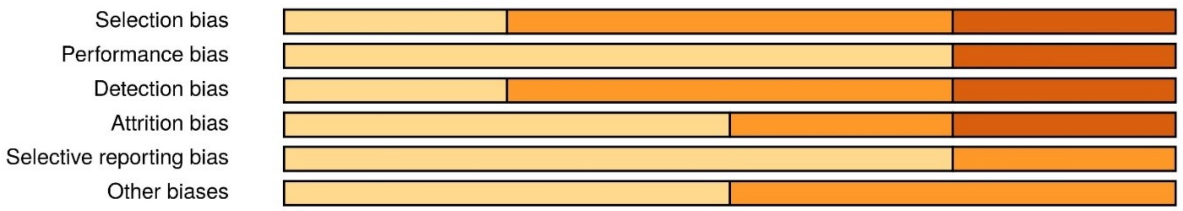

Prospective cohort and case-control studies

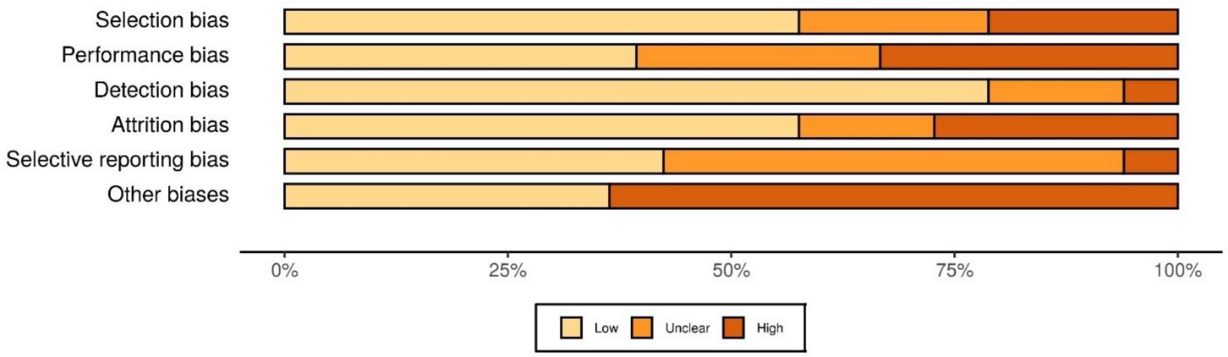

Supplemental figure 1. Summary risk of bias graphs

Randomized controlled trials: selection bias = sequence generation, allocation concealment; performance bias = blinding of participants, personnel and outcome assessors; detection bias = blinding of outcome assessment; attrition bias = incomplete outcome data; selective reporting bias = selective outcome reporting; and, other possible sources of bias = did not control for age, sex, smoking, and socioeconomic status if unbalanced at baseline, did not conduct analyses adjusting or removing those with outcome at baseline.

Prospective cohort and case-control studies: selection bias = sampling methods; performance bias = flawed measurement of exposure; detection bias = flawed measurement of outcome; attrition bias = incomplete follow-up, >20% missing data; selective reporting bias = selective/incomplete reporting of some outcomes; and, other possible biases = did not control for or examine interaction of age, sex, smoking, and socioeconomic status, for CVD, diabetes and

mortality; there was a minimum of five years of follow-up; and for prospective studies, the analysis adjusted for reverse causality by removing at least the first two years of follow-up.

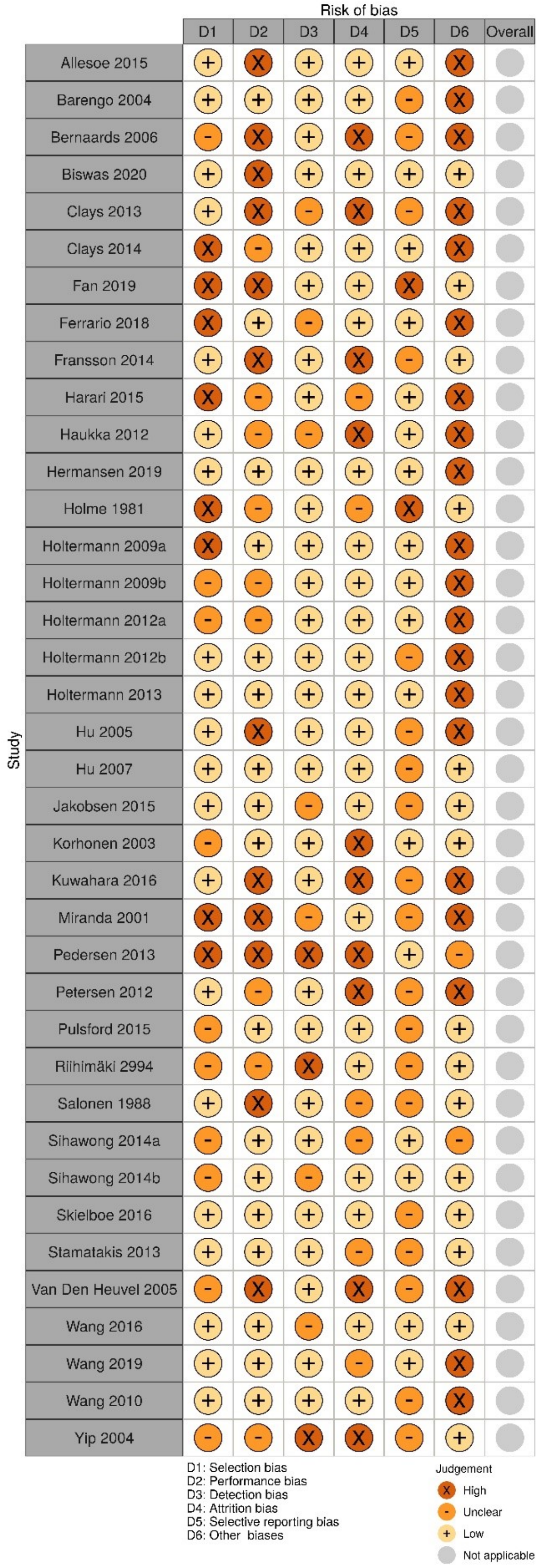

Supplemental figure 2. Risk of bias assessments per study

Supplemental table 1. Search strategies

Ovid MEDLINE(R) ALL (1946 to June 09, 2020)

#	Searches
1	*occupational health/ or *occupational diseases/ or *job description/
2	((occupation* or worker* or working or employe* or personnel or job or jobs or professional? or "at work") adj4 (physical* or labo?r or sit or sitting)).ti,kw,kf. or ((occupation* or worker* or working or employe* or personnel or job or jobs or professional? or "at work") adj4 (physical* or labo?r or sit or sitting)).ab.
3	(active adj3 (travel* or transportation or commut* or transport)).ti,kw,kf. or (active adj3 (travel* or transportation or commut* or transport)).ab. /freq=2
4	(((car or cars or vehicle? or bus or buses or subway? or streetcar? or "passive transport") adj4 commut*) or passive transport* or ((drive or driving) adj3 time)).ti,kf,kw. or (((car or cars or vehicle? or bus or buses or subway? or streetcar? or "passive transport") adj4 commut*) or passive transport* or ((drive or driving) adj3 time)).ab. /freq=2
5	or/1-4
6	*exercise/ or *circuit-based exercise/ or *cool-down exercise/ or *gymnastics/ or *high-intensity interval training/ or *muscle stretching exercises/ or *physical conditioning, human/ or *plyometric exercise/ or *resistance training/ or exp *running/ or *swimming/ or exp *walking/ or *warm-up exercise/ or exp *physical fitness/ or exp *sports/ or *dancing/ or *yoga/ or *"Physical Education and Training"/ or *motor activity/ or *movement/
7	("physical activit*" or walk* or pedestrian* or bicycl* or cycling or cyclist* or biking or bike* or "active lifestyle*" or "aerobic fitness" or "aerobic exercise*" or (running not "running water") or runner* or jog* or swim* or skate or skating or sport* or hockey or basketball or soccer or football or baseball or ski or skiing or volleyball or tennis or badminton or softball or squash or yoga or athlet* or (physical* adj2 (activit* or active or exercise*)) or ((promot* or encourag* or increas*) adj3 (play* or exercise*)) or ((exercise* or fitness or aerobic*) adj2 (regimen* or training or intervention* or program* or class* or course* or train*)) or mvpa).ti,kw,kf.
8	Sedentary Lifestyle/
9	(sedentar* or stationary).ti,kf,kw. or (sedentar* or stationary).ab. /freq=2

10	(physical inactivity or ((no or little or none or few or infrequent) adj4 (exercis* or activit*))).ti,kf,kw. or (physical inactivity or ((no or little or none or few or infrequent) adj4 (exercis* or activit*))).ab. /freq=2
11	(sitting or sit or sits or seated or recline? or reclining or lying or "lie down" or "lay down" or "laying down" or deskbound or (bound adj (desk? or chair?))).ti,kf,kw. or (sitting or sit or sits or seated or recline? or reclining or lying or "lie down" or "lay down" or "laying down" or deskbound or (bound adj (desk? or chair?))).ab. /freq=2
12	((screen? adj2 (time or watch* or view*)) or (video? adj2 (stream* or watch* or time or view*)) or television? or tv or "t.v." or computer? or laptop? or tablet? or ((brows* or surf* or use? or using) adj2 (internet or web or net))).ti,kf,kw. or ((screen? adj2 (time or watch* or view*)) or (video? adj2 (stream* or watch* or time or view*)) or television? or tv or "t.v." or computer? or laptop? or tablet? or ((brows* or surf* or use? or using) adj2 (internet or web or net))).ab. /freq=2
13	or/6-12
14	5 and 13
15	exp case-control studies/ or exp cohort studies/ or exp clinical trial/ or Observational study/ or Randomized controlled trials as Topic/ or double blind method/ or single blind method/
16	((cohort or followup or follow up or observational or longitudinal or prospective) adj2 (study or studies)).tw,kw,kf.
17	(intervention* or randomi?ed or (controlled adj2 (study or trial)) or case control* or retrospective).tw,kw,kf.
18	or/15-17
19	14 and 18
20	(child* or preschool* or pre school* or kindergarten* or toddler* or adolescent* or p?ediatric or school age or ((primary or elementary or middle or high) adj1 school*)).ti.
21	exp adult/
22	20 and 21
23	20 not 22
24	19 not 23

Embase 1941 to June 10 2020

#	Searches
1	*occupational health/ or *occupational diseases/
2	((occupation* or worker* or working or employe* or personnel or job or jobs or professional? or "at work") adj4 (physical* or labo?r or sit or sitting)).ti,kw. or ((occupation* or worker* or working or employe* or personnel or job or jobs or professional? or "at work") adj4 (physical* or labo?r or sit or sitting)).ab.
3	(active adj3 (travel* or transportation or commut* or transport)).ti,kw. or (active adj3 (travel* or transportation or commut* or transport)).ab. /freq=2
4	(((car or cars or vehicle? or bus or buses or subway? or streetcar? or "passive transport") adj4 commut*) or passive transport* or ((drive or driving) adj3 time)).ti,kw. or (((car or cars or vehicle? or bus or buses or subway? or streetcar? or "passive transport") adj4 commut*) or passive transport* or ((drive or driving) adj3 time)).ab. /freq=2
5	or/1-4
6	exp *exercise/ or exp *sport/ or exp *physical activity/ or *fitness/ or exp *athlete/ or *endurance/ or *training/ or *dancing/ or *physical education/ or exp *motor activity/ or exp *movement/
7	("physical activit*" or walk* or pedestrian* or bicycl* or cycling or cyclist* or biking or bike* or "active lifestyle*" or "aerobic fitness" or "aerobic exercise*" or (running not "running water") or runner* or jog* or swim* or skate or skating or sport* or hockey or basketball or soccer or football or baseball or ski or skiing or volleyball or tennis or badminton or softball or squash or yoga or athlet* or (physical* adj2 (activit* or active or exercise*)) or ((promot* or encourag* or increas*) adj3 (play* or exercise*)) or ((exercise* or fitness or aerobic*) adj2 (regimen* or training or intervention* or program* or class* or course* or train*)) or mvpa).ti,kw.
8	*sedentary lifestyle/ or *sedentary time/ or *sitting/
9	(sedentar* or stationary).ti,kw. or (sedentar* or stationary).ab. /freq=2
10	(physical inactivity or ((no or little or none or few or infrequent) adj4 (exercis* or activit*))).ti,kw. or (physical inactivity or ((no or little or none or few or infrequent) adj4 (exercis* or activit*))).ab. /freq=2
11	(sitting or sit or sits or seated or recline? or reclining or lying or "lie down" or "lay down" or "laying down" or deskbound or (bound adj (desk? or chair?))).ti,kw. or (sitting or sit or

	sits or seated or recline? or reclining or lying or "lie down" or "lay down" or "laying down" or deskbound or (bound adj (desk? or chair?))).ab. /freq=2
12	((screen? adj2 (time or watch* or view*)) or (video? adj2 (stream* or watch* or time or view*)) or television? or tv or "t.v." or computer? or laptop? or tablet? or ((brows* or surf* or use? or using) adj2 (internet or web or net))).ti,kw. or ((screen? adj2 (time or watch* or view*)) or (video? adj2 (stream* or watch* or time or view*)) or television? or tv or "t.v." or computer? or laptop? or tablet? or ((brows* or surf* or use? or using) adj2 (internet or web or net))).ab. /freq=2
13	or/6-12
14	5 and 13
15	case control study/ or cohort analysis/ or exp clinical trial/ or observational study/ or double blind procedure/ or single blind procedure/ or exp longitudinal study/ or prospective study/ or intervention study/ or follow up/
16	((cohort or followup or follow up or observational or longitudinal or prospective) adj2 (study or studies)).tw,kw.
17	(intervention* or randomi?ed or (controlled adj2 (study or trial)) or case control* or retrospective).tw,kw.
18	or/15-17
19	14 and 18
20	(child* or preschool* or pre school* or kindergarten* or toddler* or adolescent* or p?ediatric or school age or ((primary or elementary or middle or high) adj1 school*)).ti. or adolescent/ or exp child/
21	adult/ or exp aged/ or middle aged/ or young adult/
22	20 and 21
23	20 not 22
24	19 not 23

APA PsycInfo (1806 to June Week 1 2020)

#	Searches
1	exp occupational health/ or exp working conditions/
2	((occupation* or worker* or working or employe* or personnel or job or jobs or professional? or "at work") adj4 (physical* or labo?r or sit or sitting)).ti,id. or ((occupation* or worker* or working or employe* or personnel or job or jobs or professional? or "at work") adj4 (physical* or labo?r or sit or sitting)).ab.
3	(active adj3 (travel* or transportation or commut* or transport)).ti,id. or (active adj3 (travel* or transportation or commut* or transport)).ab. /freq=2
4	(((car or cars or vehicle? or bus or buses or subway? or streetcar? or "passive transport") adj4 commut*) or passive transport* or ((drive or driving) adj3 time)).ti,id. or (((car or cars or vehicle? or bus or buses or subway? or streetcar? or "passive transport") adj4 commut*) or passive transport* or ((drive or driving) adj3 time)).ab. /freq=2
5	or/1-4
6	exp exercise/ or physical activity/ or physical fitness/ or running/ or walking/ or swimming/ or exp sports/ or yoga/ or physical education/ or activity level/
7	("physical activit*" or walk* or pedestrian* or bicycl* or cycling or cyclist* or biking or bike* or "active lifestyle*" or "aerobic fitness" or "aerobic exercise*" or (running not "running water") or runner* or jog* or swim* or skate or skating or sport* or hockey or basketball or soccer or football or baseball or ski or skiing or volleyball or tennis or badminton or softball or squash or yoga or athlet* or (physical* adj2 (activit* or active or exercise*)) or ((promot* or encourag* or increas*) adj3 (play* or exercise*)) or ((exercise* or fitness or aerobic*) adj2 (regimen* or training or intervention* or program* or class* or course* or train*)) or mvpa).ti,id.
8	Sedentary Behavior/
9	(sedentar* or stationary).ti,id. or (sedentar* or stationary).ab. /freq=2
10	(physical inactivity or ((no or little or none or few or infrequent) adj4 (exercis* or activit*))).ti,id. or (physical inactivity or ((no or little or none or few or infrequent) adj4 (exercis* or activit*))).ab. /freq=2
11	(sitting or sit or sits or seated or recline? or reclining or lying or "lie down" or "lay down" or "laying down" or deskbound or (bound adj (desk? or chair?))).ti,id. or (sitting or sit or sits or seated or recline? or reclining or lying or "lie down" or "lay down" or "laying down" or deskbound or (bound adj (desk? or chair?))).ab. /freq=2

12	((screen? adj2 (time or watch* or view*)) or (video? adj2 (stream* or watch* or time or view*)) or television? or tv or "t.v." or computer? or laptop? or tablet? or ((brows* or surf* or use? or using) adj2 (internet or web or net))).ti,id. or ((screen? adj2 (time or watch* or view*)) or (video? adj2 (stream* or watch* or time or view*)) or television? or tv or "t.v." or computer? or laptop? or tablet? or ((brows* or surf* or use? or using) adj2 (internet or web or net))).ab. /freq=2
13	or/6-12
14	5 and 13
15	cohort analysis/ or exp experimental design/
16	((cohort or followup or follow up or observational or longitudinal or prospective) adj2 (study or studies)).tw,id.
17	(intervention* or randomi?ed or (controlled adj2 (study or trial)) or case control* or retrospective).tw,id.
18	or/15-17
19	14 and 18
20	(child* or preschool* or pre school* or kindergarten* or toddler* or adolescent* or p?ediatric or school age or ((primary or elementary or middle or high) adj1 school*)).ti.
21	19 not 20

Proquest Public Health (June 11, 2020)

((mesh.exact.explode("cohort studies") OR mesh.exact.explode(case-control studies") OR mesh.exact.explode("clinical trial") OR mesh.exact("Observational study") OR mesh.exact("Randomized controlled trials as Topic") OR mesh.exact("double blind method") OR mesh.exact("single blind method") OR noft((cohort or followup or "follow up" or observational or longitudinal or prospective) NEAR/2 (study or studies)) OR noft(intervention* or randomised or randomized or (controlled NEAR/2 (study or trial)) or "case control*" or retrospective)) AND (((MJMESH.EXACT("Yoga") OR MJMESH.EXACT("Muscle Stretching Exercises") OR MJMESH.EXACT.EXPLODE("Walking:G.11.427.410.698.277.937") OR MJMESH.EXACT("Plyometric Exercise") OR MJMESH.EXACT.EXPLODE("Running:I.03.350.750") OR MJMESH.EXACT.EXPLODE("Running:G.11.427.410.698.277.750") OR MJMESH.EXACT("Movement") OR MJMESH.EXACT("Motor Activity") OR MJMESH.EXACT.EXPLODE("Physical Fitness:I.03.450.642.845.054.800") OR MJMESH.EXACT.EXPLODE("Walking:I.03.450.642.845.940") OR MJMESH.EXACT("Gymnastics") OR MJMESH.EXACT("Dancing") OR

MJMESH.EXACT("Resistance Training") OR MJMESH.EXACT("Swimming") OR MJMESH.EXACT("Cool-Down Exercise") OR MJMESH.EXACT("High-Intensity Interval Training") OR MJMESH.EXACT.EXPLODE("Physical Fitness:G.11.427.685") OR MJMESH.EXACT("Exercise") OR MJMESH.EXACT("Physical Conditioning, Human") OR MJMESH.EXACT("Circuit-Based Exercise") OR MJMESH.EXACT.EXPLODE("Sports") OR MJMESH.EXACT.EXPLODE("Running:I.03.450.642.845.610") OR MJMESH.EXACT.EXPLODE("Running:G.11.427.410.568.610") OR MJMESH.EXACT.EXPLODE("Walking:I.03.350.937") OR MJMESH.EXACT("Physical Education and Training") OR MJMESH.EXACT.EXPLODE("Walking:G.11.427.410.568.900") OR MJMESH.EXACT("Warm-Up Exercise") OR MJMESH.EXACT.EXPLODE("Physical Fitness:N.01.400.545") OR ti,if("physical activity" or "physical activities" or walk or walked or walks or walking or pedestrian or pedestrians or bicycle or bicycling or bicycles or bicycled or cycling or cyclist or cyclists or biking or bike or bikes or biked or "active lifestyle" or "active lifestyles" or "aerobic fitness" or "aerobic exercise" or "aerobic exercises" or (running not "running water") or runner or runners or jog or jogging or joggers or jogged or jogs or swim or swimming or swims or swam or skate or skating or skates or skated or sport or sports or hockey or basketball or soccer or football or baseball or ski or skis or skied or skiing or volleyball or tennis or badminton or softball or squash or yoga or athlete or athletes or athletics or ((physical or physically) NEAR/2 (activity or activities or active or exercise or exercises or exercised or exercising)) or (active NEAR/3 (travel or travels or traveled or traveling or transportation or commute or commuting or commuter or commuters or transport)) or ((promote or promoting or promoted or promotes or encourage or encouraged or encourages or encouraging or increase or increases or increased or increasing) NEAR/3 (play or playing or played or plays or exercise or exercises or exercised or exercising)) or ((exercise or exercises or exercised or exercising or fitness or aerobic or aerobics) NEAR/2 (regimen or regimens or training or intervention or interventions or program or programs or programming or programmed or class or classes or course or courses or train or training or trains or trained)) or mvpa)) OR (MESH.EXACT("Sedentary Lifestyle") OR ti,if(sedentar* or stationary) OR ti,if(physical inactivity or ((no or little or none or few or infrequent) NEAR/4 (exercis* or activit*))) OR ti,if(sitting or sit or sits or seated or recline* or reclining or lying or "lie down" or "lay down" or "laying down" or deskbound or (bound NEAR/1 (desk* or chair*))) OR ti,if((screen* NEAR/2 (time or watch* or view*)) OR (video* NEAR/2 (stream* or watch* or time or view*)) OR television* or tv or "t.v." or computer* or laptop* or tablet* or ((brows* or surf* or use* or using) NEAR/2 (internet or web or net))))) AND (MJMESH.EXACT("occupational health") OR MJMESH.EXACT("occupational diseases") OR MJMESH.EXACT("job description") OR ti,if((occupation or occupational or occupations or worker or workers or working or employed or employee or employees or personnel or job or jobs or professional or professionals or "at work") NEAR/4 (physical or physically or labor or labour or sit or sitting)) OR ti,if(active NEAR/3 (travel or travels or traveled or travelling or transportation or commute or commuted or commuting or commutes or transport or transports or transported or transporting)) OR ti,if(((car or cars or vehicle or vehicles or bus or buses or subway or subways or streetcar or streetcars or "passive transport") NEAR/4 (commute or commuted or commuting or commutes)) or "passive transport*" or ((drive or driving) NEAR/3 time))))) NOT ti(child* or preschool* or "pre school*"

or kindergarten* or toddler* or adolescent* or pediatric or paediatric or "school age" or ((primary or elementary or middle or high) NEAR/1 school*))

Scopus (June 11, 2020)

(TITLE ((occupation OR occupational OR occupations OR worker OR workers OR working OR employed OR employee OR employees OR personnel OR job OR jobs OR professional OR professionals OR "at work") W/4 (physical OR physically OR labor OR labour OR sit OR sitting)) OR TITLE (active W/3 (travel OR travels OR traveled OR travelling OR transportation OR commute OR commuted OR commuting OR commutes OR transport OR transports OR transported OR transporting)) OR TITLE (((car OR cars OR vehicle OR vehicles OR bus OR buses OR subway OR subways OR streetcar OR streetcars OR "passive transport") W/4 (commute OR commuted OR commuting OR commutes)) OR "passive transport*" OR ((drive OR driving) W/3 time)) OR KEY ((occupation OR occupational OR occupations OR worker OR workers OR working OR employed OR employee OR employees OR personnel OR job OR jobs OR professional OR professionals OR "at work") W/4 (physical OR physically OR labor OR labour OR sit OR sitting)) OR KEY (active W/3 (travel OR travels OR traveled OR travelling OR transportation OR commute OR commuted OR commuting OR commutes OR transport OR transports OR transported OR transporting)) OR KEY (((car OR cars OR vehicle OR vehicles OR bus OR buses OR subway OR subways OR streetcar OR streetcars OR "passive transport") W/4 (commute OR commuted OR commuting OR commutes)) OR "passive transport*" OR ((drive OR driving) W/3 time))) AND ((TITLE ("physical activity" OR "physical activities" OR walk OR walked OR walks OR walking OR pedestrian OR pedestrians OR bicycle OR bicycling OR bicycles OR bicycled OR cycling OR cyclist OR cyclists OR biking OR bike OR bikes OR biked OR "active lifestyle" OR "active lifestyles" OR "aerobic fitness" OR "aerobic exercise" OR "aerobic exercises" OR (running AND NOT "running water") OR runner OR runners OR jog OR jogging OR joggers OR jogged OR jogs OR swim OR swimming OR swims OR swam OR skate OR skating OR skates OR skated OR sport OR sports OR hockey OR basketball OR soccer OR football OR baseball OR ski OR skis OR skied OR skiing OR volleyball OR tennis OR badminton OR softball OR squash OR yoga OR athlete OR athletes OR athletics OR ((physical OR physically) W/2 (activity OR activities OR active OR exercise OR exercises OR exercised OR exercising)) OR (active W/3 (travel OR travels OR traveled OR traveling OR transportation OR commute OR commuting OR commuter OR commuters OR transport)) OR ((promote OR promoting OR promoted OR promotes OR encourage OR encouraged OR encourages OR encouraging OR increase OR increases OR increased OR increasing) W/3 (play OR playing OR played OR plays OR exercise OR exercises OR exercised OR exercising)) OR ((exercise OR exercises OR exercised OR exercising OR fitness OR aerobic OR aerobics) W/2 (regimen OR regimens OR training OR intervention OR interventions OR program OR programs OR programming OR programmed OR class OR classes OR course OR courses OR train OR

training OR trains OR trained)) OR mvpa)) OR (TITLE (sedentar* OR stationary OR "physical inactivity" OR ((no OR little OR none OR few OR infrequent) W/4 (exercis* OR activit*)) OR sitting OR sit OR sits OR seated OR recline* OR reclining OR lying OR "lie down" OR "lay down" OR "laying down" OR deskbound OR (bound W/1 (desk* OR chair*)) OR (screen* W/2 (time OR watch* OR view*)) OR (video* W/2 (stream* OR watch* OR time OR view*)) OR television* OR tv OR "t.v." OR computer* OR laptop* OR tablet* OR ((brows* OR surf* OR use* OR using) W/2 (internet OR web OR net))))) AND (TITLE-ABS-KEY (((cohort OR followup OR "follow up" OR observational OR longitudinal OR prospective) near/2 (study OR studies)) OR intervention* OR randomised OR randomized OR (controlled W/2 (study OR trial)) OR "case control*" OR retrospective)) AND NOT TITLE (child* OR preschool* OR "pre school*" OR kindergarten* OR toddler* OR adolescent* OR pediatric OR paediatric OR "school age" OR ((primary OR elementary OR middle OR high) near/1 AND school*))

Supplemental table 2. Excluded studies with reasons

Title	Authors	Year	Journal	Volume	Issue	Pages	Notes
Effect of short term workplace exercise intervention on lipid profile, depression, work ability and selected physical parameters of university employees in Saudi Arabia: A randomized controlled trail	Tomar, R.; Allen, J. A.	2016	Indian Journal of Science and Technology	9	8		Exclusion reason: Outcome present at baseline
Neuromuscular exercise reduces low back pain intensity and improves physical functioning in nursing duties among female healthcare workers; Secondary analysis of a randomised controlled trial	Taulaniemi A.; et al	2019	BMC Musculoskeletal Disorders	20	1	328	Exclusion reason: Outcome present at baseline
Psychological and physical benefits of circuit weight training in law enforcement personnel	Norvell, N.; Belles, D.	1993	Journal of consulting and clinical psychology	61	3	520-7	Exclusion reason: Outcome present at baseline
The practice of active rest by workplace units improves personal relationships, mental health, and physical activity among workers	Michishita R.; et al	2017	Journal of occupational health	59	2	122-130	Exclusion reason: Outcome present at baseline
The relationship between low back pain and leisure time physical activity in a working population of cleaners--a study with weekly follow-ups for 1 year	Jespersen, Tobias; et al	2012	BMC musculoskeletal disorders	13		28	Exclusion reason: Outcome present at baseline
Effect of a general fitness program on musculoskeletal symptoms, clinical status, physiological capacity, and perceived work environment among home care service personnel	Gerdle, Bjorn; et al	1995	Journal of Occupational Rehabilitation	5	1	16-Jan	Exclusion reason: Outcome present at baseline
Health and Fitness Benefits But Low Adherence Rate: Effect of a 10-Month Onsite Physical Activity Program Among Tertiary Employees	Genin, Pauline M.; et al	2018	Journal of occupational and environmental medicine	60	9	e455-e462	Exclusion reason: Outcome present at baseline

Does leisure time physical activity protect against shoulder pain at work?	D'Onise R.; Shanahan E.M.; Gill T.; Hill C.L.	2010	Occupational Medicine	60	5	383-388	Exclusion reason: Wrong study design (e.g., cross-sectional or qualitative)
Sedentary workers have more to gain from sport physical activity in terms of cardiovascular disease risk reduction: The MONICA-Brianza, PAMELA and SEMM cohort studies	Roncaioli M.; et al	2017	European Journal of Preventive Cardiology	24	2	29	Exclusion reason: Duplicate study
Effect of physical exercise on musculoskeletal pain in multiple body regions among healthcare workers: Secondary analysis of a cluster randomized controlled trial	Jakobsen, Markus D.; et al	2018	Musculoskeletal science & practice	34		89-96	Exclusion reason: Chronic disease/condition population
Occupational and leisure time physical activity, fitness, coronary heart disease, and 22-year mortality: Results from the Kuopio ischemic heart disease risk factor study	Krause N.; Arah O.; Kauhanen J.	2017	European Journal of Preventive Cardiology	24	2	12-Nov	Exclusion reason: Unable to group workers based on OPA
High-intensity training reduces CVD risk factors among rotating shift workers: An eight-week intervention in industry	Mamen A.; et al	2020	International Journal of Environmental Research and Public Health	17	11	3943	Exclusion reason: No relevant health outcome
Physical work demands and leisure time physical activity in relation to risk for coronary heart disease	Els Clays E.; et al	2013	European Journal of Preventive Cardiology	20	1	S9	Exclusion reason: Duplicate study
The joint associations of occupational, commuting, and leisure-time physical activity, and the Framingham risk score on the 10-year risk of coronary heart disease; 17242011	Hu, G.; Tuomilehto, J.; Borodulin, K.; Jousilahti, P.	2007	European heart journal	28	4	492-498	Exclusion reason: Did not examine effect of LTPA in OPA groups
Exploring the combined effect of job strain with levels of occupational and sport physical activity on cardiovascular disease incidence: The	Ferrario M.M.; et al	2017	European Journal of Preventive Cardiology	24	2	27	Exclusion reason: Duplicate study

MONICA Brianza-PAMELA follow-up study							
Exploring the interplay between job strain and different domains of physical activity on the incidence of coronary heart disease in adult men	Ferrario M.M.; et al	2019	European Journal of Preventive Cardiology	26	17	1877-1885	Exclusion reason: Duplicate study
Work-site health promotion of frequent computer users: comparing selected interventions	Blasche, Gerhard; et al	2013	Work (Reading, Mass.)	46	3	233-41	Exclusion reason: Unable to group workers based on OPA
The cost-effectiveness of a lifestyle physical activity intervention in addition to a work style intervention on recovery from neck and upper limb symptoms and pain reduction in computer workers	Bernaards, Claire M.; et al	2011	Occupational and environmental medicine	68	4	265-72	Exclusion reason: Chronic disease/condition population
Occupation and physical activity and coronary heart disease	Cassel, J.; et al	1971	Archives of Internal Medicine	128	6	920-8	Exclusion reason: Wrong study design (e.g., cross-sectional or qualitative)
Effects of the Workplace Health Promotion Activities Soccer and Zumba on Muscle Pain, Work Ability and Perceived Physical Exertion among Female Hospital Employees	Barene, Svein; Krustrup, Peter; Holtermann, Andreas	2014	PloS one	9	12	e115059	Exclusion reason: Unable to group workers based on OPA;
The effect of a physical activity program on decreasing physical disability indicated by musculoskeletal pain and related symptoms among workers: a pilot study	Moreira-Silva, Isabel; Santos, Rute; Abreu, Sandra; Mota, Jorge	2014	International journal of occupational safety and ergonomics : JOSE	20	1	55-64	Exclusion reason: Unable to group workers based on OPA;
Individual factors, occupational loading, and physical exercise as predictors of sciatic pain	Miranda H.; et al	2002	Spine	27	10	1102-1109	Exclusion reason: Did not examine effect of LTPA in OPA groups;
Cardiovascular and other causes of death as a function of lifestyle habits in a quasi extinct middle-aged male	Menotti A.; Puddu P.E.; Maiani G.; Catasta G.	2016	International journal of cardiology	210		173-178	Exclusion reason: No leisure time PA or SB;

population. A 50-year follow-up study							
Body mass index, occupational activity, and leisure-time physical activity: an exploration of risk factors and modifiers for knee osteoarthritis in the 1946 British birth cohort	Martin, Kathryn R.; et al	2013	BMC musculoskeletal disorders	14		219	Exclusion reason: Did not examine effect of LTPA in OPA groups;
Effects of a home exercise programme on shoulder pain and functional status in construction workers	Ludewig, P. M.; Borstad, J. D.	2003	Occupational and environmental medicine	60	11	841-9	Exclusion reason: Intervention - ergonomic/productive work;
Risk factors of sciatic pain: A prospective study among middle-aged employees	Kaaria S.; et al	2011	European Journal of Pain	15	6	584-590	Exclusion reason: Did not examine effect of LTPA in OPA groups;
Physical activity protects against coronary death and deaths from all causes in middle-aged men: Evidence from a 20-year follow-up of the primary prevention study in Goteborg	Rosengren A.; Wilhelmsen L.	1997	Annals of Epidemiology	7	1	69-75	Exclusion reason: Did not examine effect of LTPA in OPA groups;
[Impact of lifestyle and obesity to the risk of type 2 diabetes: a prospective study in Jiangsu province]	Liu J.C.; et al	2012	Zhonghua yu fang yi xue za zhi [Chinese journal of preventive medicine]	46	4	311-315	Exclusion reason: Not English, French, Danish, Norwegian or Dutch
Occupational, commuting, and leisure-time physical activity in relation to total and cardiovascular mortality among Finnish subjects with type 2 diabetes	Hu, Gang; et al	2004	Circulation	110	6	666-73	Exclusion reason: Unable to group workers based on OPA
Physical demands at work, physical fitness, and 30-year ischaemic heart disease and all-cause mortality in the Copenhagen Male Study	Holtermann A.; et al	2010	Scandinavian Journal of Work, Environment and Health, Supplement	36	5	357-365	Exclusion reason: Did not examine effect of LTPA in OPA groups;
The effects of a graduated aerobic exercise programme on cardiovascular disease risk factors in	Hewitt, Jennifer A.; et al	2008	Journal of occupational medicine and toxicology (London, England)	3		7	Exclusion reason: No relevant health outcome

the NHS workplace: a randomised controlled trial							
Physical fitness and risk of myocardial infarction in Copenhagen males aged 40-59. A five- and seven-year follow-up study	Gyntelberg F.; Lauridsen L.; Schubell K.	1980	Scandinavian Journal of Work, Environment and Health	6	3	170-178	Exclusion reason: Did not examine effect of LTPA in OPA groups;
Effect of total, domain-specific, and intensity-specific physical activity on all-cause and cardiovascular mortality among hypertensive adults in China	Fan M.; et al	2018	Journal of hypertension	36	4	793-800	Exclusion reason: Did not examine effect of LTPA in OPA groups;
Work and leisure time physical activity and mortality in men and women from a general population sample	Dorn J.P.; et al	1999	Annals of Epidemiology	9	6	366-373	Exclusion reason: Did not examine effect of LTPA in OPA groups;
Sedentary behaviour and risk of mortality from all-causes and cardiometabolic diseases in adults: evidence from the HUNT3 population cohort	Chau J.Y.; et al	2015	British journal of sports medicine	49	11	737-742	Exclusion reason: Did not examine effect of LTPA in OPA groups;
Exercise DVD effect on musculoskeletal disorders in professional orchestral musicians	Chan, C.; Driscoll, T.; Ackermann, B.	2014	Occupational medicine (Oxford, England)	64	1	23-30	Exclusion reason: No leisure time PA or SB
The effectiveness of a work style intervention and a lifestyle physical activity intervention on the recovery from neck and upper limb symptoms in computer workers	Bernaards, Claire M.; et al	2007	Pain	132	02-Jan	142-53	Exclusion reason: Intervention - PA performed during work time
Associations of leisure-time and occupational physical activity and cardiorespiratory fitness with incident and recurrent major depressive disorder, depressive symptoms, and incident anxiety in a general population	Baumeister S.E.; et al	2017	Journal of Clinical Psychiatry	78	1	e41-e47	Exclusion reason: Did not examine effect of LTPA in OPA groups;

Occupational and leisure time physical activity and the risk of nonfatal acute myocardial infarction in Italy	Altieri A.; et al	2004	Annals of Epidemiology	14	7	461-466	Exclusion reason: Did not examine effect of LTPA in OPA groups;
A Longitudinal Evaluation of Risk Factors and Interactions for the Development of Nonspecific Neck Pain in Office Workers in Two Cultures	Jun D.; et al	2020	Human factors			1.87E+13	Exclusion reason: No leisure time PA or SB
Kettlebell training for musculoskeletal and cardiovascular health: A randomized controlled trial	Jay K.; et al	2011	Scandinavian Journal of Work, Environment and Health	37	3	196-203	Exclusion reason: Unable to group workers based on OPA
Leisure-time, occupational, and commuting physical activity and risk of type 2 diabetes in Japanese workers: a cohort study	Honda T.; et al	2015	BMC public health	15		1004	Exclusion reason: Did not examine effect of LTPA in OPA groups
Yoga for reducing perceived stress and back pain at work	Hartfiel, N.; et al	2012	Occupational medicine (Oxford, England)	62	8	606-12	Exclusion reason: Unable to group workers based on OPA
Physical activity and risk of myocardial infarction, cerebral stroke and death. A longitudinal study in Eastern Finland	Salonen J.T.; Puska P.; Tuomilehto J.	1982	American Journal of Epidemiology	115	4	526-537	Exclusion reason: Did not examine effect of LTPA in OPA groups;
Different domains of self-reported physical activity and risk of type 2 diabetes in a population-based Swedish cohort: the Malmo diet and Cancer study	Mutie P.M.; et al	2020	BMC public health	20	1	261	Exclusion reason: Unable to group workers based on OPA;
Case-control study of knee osteoarthritis and lifestyle factors considering their interaction with physical workload	Vrezas I.; et al	2009	International archives of occupational and environmental health			10-Jan	Exclusion reason: Did not examine effect of LTPA in OPA groups;
Reduced risk of myocardial infarction related to active commuting: Inflammatory and haemostatic	Wennberg P.; et al	2010	European Journal of Cardiovascular Prevention and Rehabilitation	17	1	56-62	Exclusion reason: Did not examine effect of LTPA in OPA groups;

effects are potential major mediating mechanisms							
Coronary artery disease risk factors in urban areas of Yazd City, Iran	Lotfi M.H.; Sadr S.M.; Nemayandea S.M.	2011	Asia-Pacific Journal of Public Health / Asia-Pacific Academic Consortium for Public Health	23	4	534-543	Exclusion reason: Did not examine effect of LTPA in OPA groups;
Physical activity, musculoskeletal disorders and cardiovascular risk factors in male physical education teachers	Pihl E.; Matsin T.; Jurimae T.	2002	Journal of Sports Medicine and Physical Fitness	42	4	466-471	Exclusion reason: No relevant health outcome;
Associations of leisure-time, occupational, and commuting physical activity with risk of depressive symptoms among Japanese workers: A cohort study	Kuwahara K.; et al	2015	International Journal of Behavioral Nutrition and Physical Activity	12	1	119	Exclusion reason: Unable to group workers based on OPA
Leisure-time, occupational, household physical activity and insulin resistance (HOMAIR) in the Midlife in the United States (MIDUS) national study of adults	Tsenkova V.K.	2017	Preventive Medicine Reports	5		224-227	Exclusion reason: Did not examine effect of LTPA in OPA groups;
Occupational and leisure time physical inactivity and the risk of type II diabetes and hypertension among Mexican adults: A prospective cohort study	Medina C.; et al	2018	Scientific reports	8	1	5399	Exclusion reason: Unable to group workers based on OPA;
Occupational physical activity and cardiovascular risk profile among the adult population of the southern cone of Latin America	Poggio R.; et al	2017	Circulation	135			Exclusion reason: Unable to group workers based on OPA;
The effects of commuting activity and occupational and leisure time physical activity on risk of myocardial infarction	Wennberg P.; et al	2006	European Journal of Cardiovascular Prevention and Rehabilitation	13	6	924-930	Exclusion reason: Did not examine effect of LTPA in OPA groups;
Does sitting at work cause low back pain?; 11851181	Hartvigsen, J.; et al	2002	Ugeskrift for laeger	164	6	759-761	Exclusion reason: Systematic review;

Effectiveness of small daily amounts of progressive resistance training for frequent neck/shoulder pain: randomised controlled trial	Andersen, Lars L.; et al	2011	PainÂ®	152	2	440-446	Exclusion reason: Intervention - ergonomic/productive work;
A prospective cohort study of risk factors for disability retirement because of back pain in the general working population	Hagen, KÃ¥re B.; et al	2002	Spine	27	16	1790-1796	Exclusion reason: Did not examine effect of LTPA in OPA groups;
Risk factors for new episodes of sick leave due to neck or back pain in a working population. A prospective study with an 18-month and a three-year follow-up	Bergstrom, G.; et al	2007	Occupational and environmental medicine	64	4	279-287	Exclusion reason: Unable to group workers based on OPA;
Effectiveness of dynamic muscle training, relaxation training, or ordinary activity for chronic neck pain: randomised controlled trial	Viljanen, Matti; et al	2003	Bmj	327	7413	475	Exclusion reason: Intervention - ergonomic/productive work;
Effects of leisure-time and occupational physical activity on total mortality risk in NHANES III according to sex, ethnicity, central obesity, and age	Richard, A.; et al	2015	Journal of physical activity & health	12	2	184-192	Exclusion reason: Did not examine effect of LTPA in OPA groups;
All-cause mortality associated with physical activity during leisure time, work, sports, and cycling to work	Andersen, Lars Bo; et al	2000	Archives of Internal Medicine	160	11	1621-1628	Exclusion reason: Did not examine effect of LTPA in OPA groups;
The effects of yoga on stress and psychological health among employees: An 8- and 16-week intervention study	Maddux, Rachel E.; et al	2018	Anxiety, Stress & Coping: An International Journal	31	2	121-134	Exclusion reason: Unable to group workers based on OPA
Predictors of back pain in a general population cohort	Kopec, Jacek A.; Sayre, Eric C.; Esdaile, John M.	2004	Spine	29	1	70-77	Exclusion reason: Not working population;
To what extent do current and past physical and psychosocial occupational factors explain care-seeking for low back pain in a	VingÃ¥rd, Eva; et al	2000	Spine	25	4	493-500	Exclusion reason: Did not examine effect of LTPA in OPA groups;

working population?: results from the Musculoskeletal Intervention Center-NorrtÃ¤lje study							
Disorders of the back and spine in construction workers: prevalence and prognostic value for disability	Rothenbacher, Dietrich; et al	1997	Spine	22	13	1481-1486	Exclusion reason: No leisure time PA or SB;
The relationships of change in physical activity with change in depression, anxiety, and burnout: A longitudinal study of Swedish healthcare workers.	Lindwall, Magnus; et al	2014	Health Psychology	33	11	1309	Exclusion reason: Unable to group workers based on OPA;
Impact of a physical activity program on the anxiety, depression, occupational stress and burnout syndrome of nursing professionals	Freitas, Anderson Rodrigues; et al	2014	Revista latino-americana de enfermagem	22	2	332-336	Exclusion reason: Intervention - PA performed during work time;
A randomized controlled intervention trial to relieve and prevent neck/shoulder pain	Andersen, Lars L.; et al	2008	Medicine and science in sports and exercise	40	6	983	Exclusion reason: Intervention - PA performed during work time;
An effective exercise-based intervention for improving mental health and quality of life measures: a randomized controlled trial	Atlantis, E.; et al	2004	Preventive medicine	39	2	424-434	Exclusion reason: Unable to group workers based on OPA;
Physical activity and risk of cardiovascular diseaseâ€"a meta-analysis of prospective cohort studies	Li, Jian; Siegrist, Johannes	2012	International journal of environmental research and public health	9	2	391-407	Exclusion reason: Systematic review;
Daily sedentary time and risk of cardiovascular disease: The national finrisk 2002 study	Borodulin K.; et al	2013	Circulation	127	12		Exclusion reason: Unable to group workers based on OPA;
The effect of physical activity on neck and upper limb symptoms	Van Den Heuve S.; et al	2007	Huisarts en Wetenschap	50	3	91-94	Exclusion reason: Duplicate study;
Occupational and leisure time physical activity: Trend in the Italian population	Trojani, M.; et al	2006	Giornale italiano di cardiologia	7	7	487-497	Exclusion reason: Not English, French, Danish, Norwegian or Dutch;

The relationship between physical activity level and selected cardiovascular risk factors and mortality of males <= 50 years in Poland - the results of follow-up of participants of National Multicenter Health Survey WOBASZ	Smigielski J.; et al	2016	International journal of occupational medicine and environmental health	29	4	633-648	Exclusion reason: Did not examine effect of LTPA in OPA groups;
Ten-year mortality from coronary heart disease among 172,000 men classified by occupational physical activity	Menotti, A.; Puddu, V.	1979	Scandinavian journal of work, environment & health	5	2	100-8	Exclusion reason: No leisure time PA or SB;
Leisure time physical activity initiated by employer and its health effects. A 15-month follow-up study	Marit Skogstad M.; et al	2017	European Journal of Preventive Cardiology	24	1	S140-S141	Exclusion reason: No relevant health outcome;
Physical activity levels, ownership of goods promoting sedentary behaviour and risk of myocardial infarction: results of the INTERHEART study	Held, Claes; et al	2012	European heart journal	33	4	452-66	Exclusion reason: Did not examine effect of LTPA in OPA groups;
Passive and mentally-active sedentary behaviors and incident major depressive disorder: A 13-year cohort study	Hallgren M.; et al	2018	Journal of affective disorders	241	Journal Article	579-585	Exclusion reason: Did not examine effect of LTPA in OPA groups;
Occupational and leisure-time physical activity differentially predict 6-year incidence of stroke and transient ischemic attack in women	Hall C.; et al	2019	Scandinavian Journal of Work, Environment and Health	45	3	267-279	Exclusion reason: Did not examine effect of LTPA in OPA groups;
Does an exercise intervention improving aerobic capacity among construction workers also improve musculoskeletal pain, work ability, productivity, perceived physical exertion, and sick leave?: a randomized controlled trial	Gram, Bibi; et al	2012	Journal of occupational and environmental medicine	54	12	1520-6	Exclusion reason: Intervention - PA performed during work time;

Types of sedentary behavior and risk of cardiovascular events and mortality in blacks: The Jackson heart study	Garcia J.M.; et al	2019	Journal of the American Heart Association	8	13	e010406	Exclusion reason: Did not examine effect of LTPA in OPA groups;
Effect of combined occupational tasks on cardiovascular events: PRIME study	Esquirol Y.; et al	2016	Archives of Cardiovascular Diseases Supplements	8	1	13-Dec	Exclusion reason: Did not examine effect of LTPA in OPA groups;
Could occupational physical activity mitigate the link between moderate kidney dysfunction and coronary heart disease?	Esquirol Y.; et al	2014	International journal of cardiology	177	3	1036-1041	Exclusion reason: Did not examine effect of LTPA in OPA groups;
Potential gain of occupational and leisure physical activities on the link between occupational social inequalities and cardiovascular diseases: The PRIME study	Esquirol Y.; et al	2016	European heart journal	37	Journal Article	334	Exclusion reason: Did not examine effect of LTPA in OPA groups;
Workplace exercise intervention to prevent depression: A pilot randomized controlled trial	De Zeeuw E.L.E.J.; et al	2010	Mental Health and Physical Activity	3	2	72-77	Exclusion reason: Intervention - PA performed during work time;
Time of sitting as an independent risk factor of CHD, type 2 diabetes, hypertension in PURE Poland study	Dagmara Gawel-Dabrowska D.; et al	2018	European Journal of Preventive Cardiology	25	2	S33	Exclusion reason: Not working population;
The combined relationship of occupational and leisure-time physical activity with all-cause mortality among men, accounting for physical fitness	Clays E.; et al	2014	American Journal of Epidemiology	179	5	559-566	Exclusion reason: Unable to group workers based on OPA
The effectiveness of a multidisciplinary exercise program in managing work-related musculoskeletal symptoms for low-skilled workers in the low-income community: A pre-post-follow-up study	Cheung K.; et al.	2019	International Journal of Environmental Research and Public Health	16	9	1548	Exclusion reason: Unable to group workers based on OPA;

Walks4Work: assessing the role of the natural environment in a workplace physical activity intervention	Brown, Daniel K.; et al	2014	Scandinavian journal of work, environment & health	40	4	390-9	Exclusion reason: No relevant health outcome;
Relationships between physical activity across lifetime and health outcomes in older adults: Results from the NuAge cohort	Boisvert-Vigneault K.; et al	2016	Preventive medicine	91	Journal Article	37-42	Exclusion reason: Unable to group workers based on OPA
Effect of an exercise programme for the prevention of back and neck pain in poultry slaughterhouse workers	Bertozzi, Lucia; et al	2015	Occupational therapy international	22	1	36-42	Exclusion reason: Intervention - ergonomic/productive work;
Physical Activity in Healthcare Workers with Low Back Pain. Effects of the Back-FIT Randomized Trial	Bernardelli G.; et	2020	Journal of occupational and environmental medicine		Journal Article		Exclusion reason: Intervention - PA performed during work time;
Association of Physical Activity With Risk of Major Cardiovascular Diseases in Chinese Men and Women	Bennett, Derrick A.; et al.	2017	JAMA cardiology	2	12	1349-1358	Exclusion reason: Unable to group workers based on OPA;
Low physical activity as a predictor for antihypertensive drug treatment in 25-64-year-old populations in Eastern and south-western Finland	Barengo N.C.; et al	2005	Journal of hypertension	23	2	293-299	Exclusion reason: Did not examine effect of LTPA in OPA groups;
Comparing the impact of specific strength training vs general fitness training on professional symphony orchestra musicians: A feasibility study	Andersen L.N.; et al	2017	Medical Problems of Performing Artists	32	2	94-100	Exclusion reason: Did not examine effect of LTPA in OPA groups;
Occupational physical activity and risk of ischaemic heart disease in women: The modifying effect of leisure time physical activity, hypertension and influence at work	Allesoe K.	2017	European Journal of Preventive Cardiology	24	2	21-22	Exclusion reason: Duplicate study;
High prevalence of sedentary risk factors amongst university employees and potential health	Alkhatib A.	2015	Work (Reading, Mass.)	52	3	589-595	Exclusion reason: No relevant health outcome;

benefits of campus workplace exercise intervention							
Effects of physical activity programmes in the workplace (PAPW) on the perception and intensity of musculoskeletal pain experienced by garment workers	Pereira, et al	2013	Work (Reading, Mass.)	44	4	415-21	Exclusion reason: No leisure time PA or SB;
Work and cardiovascular health: Viewpoint of occupational physiology	Ilmarinen J.	1989	Annals of Medicine	21	3	209-214	Exclusion reason: Did not examine effect of LTPA in OPA groups;
Low back pain and physical activity--A 6.5 year follow-up among young adults in their transition from school to working life	Lunde, Lars-Kristian; et al	2015	BMC public health	15	Journal Article	1115	Exclusion reason: Not working population;
Leisure time, occupational, and commuting physical activity and the risk of stroke		2005	Journal of Vascular Surgery	42	4	819	Exclusion reason: Duplicate study;
Protection against ischemic heart disease in the Belgian Physical Fitness Study: Physical fitness rather than physical activity?	Sobolski J.; et al	1987	American Journal of Epidemiology	125	4	601-610	Exclusion reason: Did not examine effect of LTPA in OPA groups;
Relationship between leisure time and occupational physical activity and incidence of hypertension and diabetes: The national population health survey (1994-2009)	Smith B.T.; et al	2012	American Journal of Epidemiology	175	Journal Article	S77	Exclusion reason: Unable to group workers based on OPA
Occupational physical activity in relation to risk of cardiovascular mortality: The Japan Collaborative Cohort Study for Evaluation for Cancer Risk (JACC Study)	Hayashi R.; et al	2016	Preventive medicine	89	Journal Article	286-291	Exclusion reason: No leisure time PA or SB;
Impact of occupational physical activity and related tasks on cardiovascular disease: emerging opportunities for prevention?	Esquirol, Yolande; et al	2013	International journal of cardiology	168	4	Aug-4475	Exclusion reason: Did not examine effect of LTPA in OPA groups;

Physical activity across the whole-of-day and vascular structure and function: The paris prospective study III	Climie R.; et al	2018	Journal of hypertension	36	Journal Article	e301	Exclusion reason: Unable to group workers based on OPA
Musculoskeletal problems and physical activity. Results from a long-term study	Moe K.; Thom E.	1997	Tidsskrift for den Norske laegeforening	117	29	4258-4261	Exclusion reason: Unable to group workers based on OPA
Can active commuting increase quality of life? Three-month results from a randomized control trial	Mutrie, N.; et al	2000	Journal of sports sciences	18	1	18-19	Exclusion reason: Unable to group workers based on OPA
A "Sit Less, Walk More" Workplace Intervention for Office Workers: Long-Term Efficacy of a Quasi-Experimental Study	Lin, Yun-Ping; et al	2018	Journal of occupational and environmental medicine	60	6	e290-e299	Exclusion reason: No relevant health outcome;
[Risk factors and mortality--a follow-up of the Oslo Health Study from 1972-73]	Holme, Ingar; Tonstad, Serena	2011	Tidsskrift for den Norske laegeforening : tidsskrift for praktisk medicin, ny raekke	131	5	456-60	Exclusion reason: Did not examine effect of LTPA in OPA groups;
Effects of an exercise program on musculoskeletal symptoms and physical capacity among nursing staff	Skargren, E.; Oberg, B.	1996	Scandinavian Journal of Medicine & Science in Sports	6	2	122-30	Exclusion reason: No leisure time PA or SB;
Twelve years experience of a fitness program for the salaried employees of a Toronto life assurance company	Shephard, R. J.	1992	American Journal of Health promotion : AJHP	6	4	292-301	Exclusion reason: Unable to group workers based on OPA;
An organizational behavior-based intervention model for worksite health promotion: Change in physical fitness level at a west coast Naval air station after one year	Linenger, Jerry M.	1991	Dissertation Abstracts International	51	7-	3347	Exclusion reason: No leisure time PA or SB; C
Incidence and Risk Factors of Neck Discomfort: A 6-month Sedentary-worker Cohort Study	Jau-Yih Tsauo; et al	2007	Journal of Occupational Rehabilitation	17	2	171-9	Exclusion reason: No leisure time PA or SB;
Hip pain onset in relation to cumulative workplace and leisure time mechanical load: a population based case-control study	Pope, D. P.; et al	2003	Annals of the Rheumatic Diseases	62	4	322-6	Exclusion reason: Did not examine effect of LTPA in OPA groups;

Is mode of transport to work associated with mortality in the working-age population? Repeated census-cohort studies in New Zealand 1996, 2001 and 2006	Shaw C.; et al	2020	International journal of epidemiology		Journal Article		Exclusion reason: Unable to group workers based on OPA;
Predictive factors for development of neck pain among computer users	Zomalheto, Zavier; et al	2012	Joint bone spine	79	6	632-3	Exclusion reason: No leisure time PA or SB;
Influence of Individual Determinants on Physical Activity at Work and During Leisure Time in Soldiers: A Prospective Surveillance Study	Schulze C.; et al	2016	U.S.Army Medical Department journal		Journal Article	53-59	Exclusion reason: Wrong study design (e.g., cross-sectional or qualitative);
Secular trends in acute myocardial infarction in relation to physical activity in the general Danish population	Sjol, A.; et al	2003	Scandinavian Journal of Medicine & Science in Sports	13	4	224-30	Exclusion reason: Did not examine effect of LTPA in OPA groups;
Efficacy of preventing ischemic heart disease and arterial hypertension at a factory manufacturing computer equipment	Mikunis R.I.; et al	1987	Terapevticheskii arkhiv	59	5	118-122	Exclusion reason: Not English, French, Danish, Norwegian or Dutch;
Association between objectively measured physical behaviour and neck- and/or low back pain: A systematic review	Overas C.K.; et al	2020	European Journal of Pain (United Kingdom)		Journal Article		Exclusion reason: Systematic review
Physical activity and cardiovascular mortality - Disentangling the roles of work, fitness, and leisure; 20686737	Krause, N.	2010	Scandinavian Journal of Work, Environment and Health	36	5	349-355	Exclusion reason: Commentary;
Physical activity levels and changes in relation to longevity: A prospective study of Swedish women	Lissner L.; et al	1996	American Journal of Epidemiology	143	1	54-62	Exclusion reason: Did not examine effect of LTPA in OPA groups;
Comparison of randomized and non-randomized controlled trials evidence regarding the effectiveness of workplace exercise on musculoskeletal pain control	Moreira, R. F. C.; et al	2012	Work (Reading, Mass.)	41 Suppl 1	Journal Article	Sep-4782	Exclusion reason: Systematic review;
Impact of Workplace Physical Activity Interventions on Physical Activity and	Reed, Jennifer L.; et al	2017	Circulation.Cardiovascular quality and outcomes	10	2		Exclusion reason: Systematic review;

Cardiometabolic Health Among Working-Age Women: A Systematic Review and Meta-Analysis							
The effects of exercise program on burnout and metabolic syndrome components in banking and insurance workers	Tsai, Han Hui; et al	2013	Industrial health	51	3	336-46	Exclusion reason: No relevant health outcome;
The Effects of Workplace Physical Activity Programs on Musculoskeletal Pain: A Systematic Review and Meta-Analysis	Moreira-Silva I.; et al	2016	Workplace health & safety	64	5	210-222	Exclusion reason: Systematic review
Health-related effects of worksite interventions involving physical exercise and reduced workhours	Schwarz, Ulrica von Thiele, et al	2008	Scandinavian journal of work, environment & health	34	3	179-88	Exclusion reason: Unable to group workers based on OPA;
Does aerobic exercise improve or impair cardiorespiratory fitness and health among cleaners? A cluster randomized controlled trial	Korshoj M.; et al	2015	Scandinavian Journal of Work, Environment and Health	41	2	140-152	Exclusion reason: Intervention - PA performed during work time;
Leisure time physical activity moderates the relationship between work stress and older workers' mental health	Farr, Dana	2018	Dissertation Abstracts International: Section B: The Sciences and Engineering	79	2-	No-Specified	Exclusion reason: Unable to group workers based on OPA;
The impact of lifestyle intervention on sedentary time in individuals at high risk of diabetes	Rockette-Wagner B.; et al	2015	Diabetologia		Journal Article		Exclusion reason: Unable to group workers based on OPA;
On the Effects of a Workplace Fitness Program upon Pain Perception: a Case Study Encompassing Office Workers in a Portuguese Context	Macedo, Angela C.; et al	2011	Journal of Occupational Rehabilitation	21	2	228-33	Exclusion reason: No leisure time PA or SB;
Effectiveness of a Worksite Intervention for Male Construction Workers on Dietary and Physical Activity Behaviors, Body Mass Index, and Health Outcomes: Results of a Randomized Controlled Trial	Laura, Viester; et al	2018	American Journal of Health promotion : AJHP	32	3	795-805	Exclusion reason: No relevant health outcome;

Associations of objectively measured sitting and standing with low-back pain intensity: A 6-month follow-up of construction and healthcare workers	Lunde L.-K.; et al	2017	Scandinavian Journal of Work, Environment and Health	43	3	269-278	Exclusion reason: No leisure time PA or SB;
Effects of active commuting on exercise capacity, cardiovascular risk, body composition and quality of life: Results of a randomized-controlled study	Rossi V.A.; et al	2019	European Journal of Preventive Cardiology	26	Journal Article	S77	Exclusion reason: Unable to group workers based on OPA;
Multi-wave cohort study of sedentary work and risk of ischemic heart disease	Moller, Simone Visbjerg; et al	2016	Scandinavian journal of work, environment & health	42	1	43-51	Exclusion reason: Did not examine effect of LTPA in OPA groups;
Long term effects on risk factors for cardiovascular disease after 12-months of aerobic exercise intervention - A worksite RCT among cleaners	Korshoj M.; et al	2016	PLoS ONE	11	8	e0158547	Exclusion reason: Intervention - PA performed during work time;
Decrease in musculoskeletal pain after 4 and 12 months of an aerobic exercise intervention: a worksite RCT among cleaners	Korshoj M.; et al	2018	Scandinavian Journal of Public Health	46	8	846-853	Exclusion reason: Intervention - PA performed during work time;
Daily physical activity and risk of atrial fibrillation	Kellerth O.; et al	2014	European heart journal	35	Journal Article	436	Exclusion reason: Did not examine effect of LTPA in OPA groups;
Diet, physical exercise and cognitive behavioral training as a combined workplace based intervention to reduce body weight and increase physical capacity in health care workers - a randomized controlled trial	Christensen, Jeanette R.; et al	2011	BMC Public Health	11	Journal Article	671	Exclusion reason: No relevant health outcome;
Contributions of leisure-time vs. occupational physical activity to variation in cardiorespiratory fitness and cardiometabolic risk profile:	Almeras N.; et al	2016	Canadian Journal of Cardiology	32	10	S241	Exclusion reason: Commentary;

Results from a workplace health management program							
Cardiorespiratory fitness, cardiovascular workload and risk factors among cleaners; a cluster randomized worksite intervention	Korshoj M.; et al	2012	BMC public health	12	Journal Article	645	Exclusion reason: Study protocol;
Systematic review and meta-analysis of reduction in all-cause mortality from walking and cycling and shape of dose response relationship	Kelly P.; et al	2014	International Journal of Behavioral Nutrition and Physical Activity	11	1	132	Exclusion reason: Systematic review;
Compliance with Physical Activity Recommendations and Associated Factors in a Cohort of Spanish Adult Workers	Moreno-Franco B.; et al	2015	Revista espanola de salud publica	89	5	447-457	Exclusion reason: Not English, French, Danish, Norwegian or Dutch;
A physical fitness programme during paid working hours - impact on health and work ability among women working in the social service sector: A three year follow up study	VingÃ¥rd, Eva; et al	2009	Work	34	3	339	Exclusion reason: No relevant health outcome;
Occupational Physical Activity and Cardiovascular Risk Factors Profile in the Adult Population of the Southern Cone of Latin America: Results From the CESCAS I Study	Poggio R.; et al	2018	Journal of occupational and environmental medicine	60	9	e470-e475	Exclusion reason: Wrong study design (e.g., cross-sectional or qualitative);
A randomized-controlled study on active commuting to work: Effects on cardiovascular risk, exercise capacity, body composition and quality of life	Niederseer D.; et al	2019	Kardiovaskulare Medizin	22	3		Exclusion reason: Unable to group workers based on OPA;
Associations of context-specific sitting time with markers of cardiometabolic risk in Australian adults	Dempsey P.C.; et al	2018	International Journal of Behavioral Nutrition and Physical Activity	15	1	114	Exclusion reason: Not working population;
Complexity of the relation between physical activity and stroke: a meta-analysis	Oczkowski, Wieslaw	2005	Clinical journal of sport medicine : official journal of the Canadian Academy of Sport Medicine	15	5	399	Exclusion reason: Systematic review;

Improving office workers' mental health and cognition: a 3-arm cluster randomized controlled trial targeting physical activity and sedentary behavior in multi-component interventions	Nooijen C.F.J.; et al	2019	BMC public health	19	1	266	Exclusion reason: Study protocol;
Short-Term Efficacy of a "Sit Less, Walk More" Workplace Intervention on Improving Cardiometabolic Health and Work Productivity in Office Workers	Lin, Yun-Ping; et al	2017	Journal of occupational and environmental medicine	59	3	327-334	Exclusion reason: No relevant health outcome;
Work-directed rehabilitation or physical activity to support work ability and mental health in common mental disorders: a pilot randomized controlled trial	Danielsson L.; et al	2020	Clinical rehabilitation	34	2	170-181	Exclusion reason: Chronic disease/condition population;
Mandatory exercise and heart disease risk in fire fighters. A longitudinal study	Green, J. S.; Crouse, S. F.	1991	International archives of occupational and environmental health	63	1	May-51	Exclusion reason: No relevant health outcome;
The relationships between active transport to work or school and cardiovascular health or body weight: A systematic review; 23572375	Xu, H.; Wen, L. M.; Rissel, C.	2013	Asia-Pacific Journal of Public Health	25	4	298-315	Exclusion reason: Systematic review;
Physical activity and stroke. A meta-analysis of observational data	Wendel-Vos, G.; et al	2004	International journal of epidemiology	33	4	787-98	Exclusion reason: Systematic review;
Workplace interventions for increasing standing or walking for decreasing musculoskeletal symptoms in sedentary workers	Parry, Sharon P.; et al	2019	The Cochrane database of systematic reviews	2019	11		Exclusion reason: Systematic review ;
Reducing occupational sedentary time: a systematic review and meta-analysis of evidence on activity-permissive workstations	Neuhaus, M.; et al	2014	Obesity reviews : an official journal of the International Association for the Study of Obesity	15	10	822-38	Exclusion reason: Systematic review;

Effect of workplace physical activity interventions on the cardio-metabolic health of working adults: Systematic review and meta-analysis	Mulchandani R.; et al	2019	International Journal of Behavioral Nutrition and Physical Activity	16	1	134	Exclusion reason: Systematic review;
An integrative, systematic review exploring the research, effectiveness, adoption, implementation, and maintenance of interventions to reduce sedentary behaviour in office workers	Macdonald B.; et al	2018	International Journal of Environmental Research and Public Health	15	12	2876	Exclusion reason: Systematic review;
Leisure time physical activity and mortality in Hong Kong: Case-control study of all adult deaths in 1998	Lam T.-H.; et al.	2004	Annals of Epidemiology	14	6	391-398	Exclusion reason: Did not examine effect of LTPA in OPA groups;
Workplace physical activity interventions: a systematic review	To Q.G.; et al	2013	American Journal of Health promotion : AJHP	27	6	e113-e123	Exclusion reason: Systematic review;
Effects of physical exercise at the workplace for treatment of low back pain: a systematic review with meta-analysis	Maciel, Roberto Rodrigues Bandeira Tosta; et al	2018	Revista brasileira de medicina do trabalho : publicacao oficial da Associacao Nacional de Medicina do Trabalho-ANAMT	16	2	225-235	Exclusion reason: Systematic review;
Physical activity and risk of cardiovascular disease: What does the new epidemiological evidence show?	Li J.; Loerbroks A.; Angerer P.	2013	Current opinion in cardiology	28	5	575-583	Exclusion reason: Systematic review;
Are incremental changes in physical activity and sedentary behaviours associated with improved employee health?: A 12-month prospective study in five organisations	Jackson C.; et al	2014	International Journal of Workplace Health Management	7	1	16-39	Exclusion reason: Unable to group workers based on OPA;
Association between physical activity, occupational sitting time and mortality in a general population: An 18-year prospective survey in Tanushimaru, Japan	Sakaue A.; et al	2020	European Journal of Preventive Cardiology	27	7	758-766	Exclusion reason: Unable to group workers based on OPA;

Can leisure-time physical activity improve health checkup results? Evidence from Japanese occupational panel data	Oshio, Takashi; Tsutsumi, Akizumi; Inoue, Akiomi	2016	Journal of occupational health	58	4	354-64	Exclusion reason: Unable to group workers based on OPA;
Using pedometers to promote physical activity among working urban women	Garbers S.; Nelson J.A.; Rosenberg T.; Chiasson M.A.	2006	Preventing chronic disease	3	2	A67	Exclusion reason: Unable to group workers based on OPA;
Work related physical activity and risk of a hospital discharge diagnosis of atrial fibrillation or flutter: The Danish Diet, Cancer, and Health Study	Frost L.; Frost P.; Vestergaard P.	2005	Occupational and environmental medicine	62	1	49-53	Exclusion reason: Did not examine effect of LTPA in OPA groups;
Mindful2Work: Effects of combined physical exercise, yoga, and mindfulness meditations for stress relieve in employees. A proof of concept study	de Bruin, Esther,I.; et al.	2017	Mindfulness	8	1	204-217	Exclusion reason: Unable to group workers based on OPA;
Walking and sports participation and mortality from coronary heart disease and stroke	Noda, Hiroyuki; et al	2005	Journal of the American College of Cardiology	46	9	1761-7	Exclusion reason: Unable to group workers based on OPA;
Physical activity, cardiorespiratory fitness and carotid intima thickness: Sedentary occupation as risk factor for atherosclerosis and obesity	Leischik R.; et al	2015	European review for medical and pharmacological sciences	19	17	3157-3168	Exclusion reason: No relevant health outcome;
Smoking, alcohol consumption, physical activity, and family history and the risks of acute myocardial infarction and unstable angina pectoris: A prospective cohort study	Merry A.H.H.; et al	2011	BMC Cardiovascular Disorders	11	Journal Article	13	Exclusion reason: Unable to group workers based on OPA;
Physical activity and physical demand on the job and risk of cardiovascular disease and death: the Framingham Study	Kannel, W. B.; et al	1986	American Heart Journal	112	4	820-5	Exclusion reason: Did not examine effect of LTPA in OPA groups;
Physical and psychosocial factors related to low back pain during a 24-	Thorbjornsson C.B.; et al	2000	Spine	25	3	369-375	Exclusion reason: Did not examine effect of LTPA in OPA groups;

year period: A nested case-control analysis							
[Physical activity, physical fitness and coronary disease]	Sobolski, J.; et al	1985	Revue d'epidemiologie et de sante publique	33	05-Apr	358-60	Exclusion reason: Unable to group workers based on OPA;
A longitudinal observational study of back pain incidence, risk factors and occupational physical activity in Swedish marine trainees	Monnier A.; et al	2019	BMJ Open	9	5	25150	Exclusion reason: Unable to group workers based on OPA;
Occupational physical activity, metabolic syndrome and risk of death from all causes and cardiovascular disease in the HUNT 2 cohort study	Moe, Borge; et al	2013	Occupational and environmental medicine	70	2	86-90	Exclusion reason: Did not examine effect of LTPA in OPA groups;
Self-reported occupational physical activity and cardiorespiratory fitness: Importance for cardiovascular disease and all-cause mortality	Holtermann A.; et al	2016	Scandinavian Journal of Work, Environment and Health	42	4	291-298	Exclusion reason: Did not examine effect of LTPA in OPA groups;
The predictive relationship of physical activity on the incidence of low back pain in an occupational cohort	Thiese, Matthew S.; et al	2011	Journal of occupational and environmental medicine	53	4	364-71	Exclusion reason: Unable to group workers based on OPA;
Physical activity and the incidence of type 2 diabetes in the Shanghai women's health study	Villegas R.; Shu X.-O.; Li H.; et al	2006	International journal of epidemiology	35	6	1553-1562	Exclusion reason: Unable to group workers based on OPA;
The effect of a worksite based walking programme on cardiovascular risk in previously sedentary civil servants [NCT00284479]	Murphy, Marie H.; et al	2006	BMC public health	6	Journal Article	136	Exclusion reason: No relevant health outcome;
Ischaemic heart disease, stroke and total mortality in women--results from a prospective population study in Gothenburg, Sweden	Lapidus L.	1985	Acta medica Scandinavica.Supplementum	705	Journal Article	1-42	Exclusion reason: Did not examine effect of LTPA in OPA groups;

Occupational physical activity and 20-year incidence of acute myocardial infarction: Results from the kuopio ischemic heart disease risk factor study	Krause N.; Brand R.J.; Arah O.A.; Kauhanen J.	2015	Scandinavian Journal of Work, Environment and Health	41	2	124-139	Exclusion reason: Did not examine effect of LTPA in OPA groups;
Musculoskeletal injuries in physical education versus non-physical education teachers: a prospective study	Goossens L.; et al	2016	Journal of sports sciences	34	12	1107-1115	Exclusion reason: No relevant health outcome;
The International Universities Walking Project: Employee step counts, sitting times and health status	Gilson, N.; et al	2008	International Journal of Workplace Health Management	1	3	152-161	Exclusion reason: Wrong study design (e.g., cross-sectional or qualitative);
Social class and cardiovascular risk factors in Danish men	Moller L.; Kristensen T.S.; Hollnagel H.	1991	Scandinavian journal of social medicine	19	2	116-126	Exclusion reason: No relevant health outcome;
Physical activity and cardiovascular health - Work and leisure differ	Kukkonen-Harjula K.	2007	Scandinavian Journal of Work, Environment and Health	33	6	401-404	Exclusion reason: Commentary
Association between Obesity and Carotid Intima-Media Thickness in Korean Office Workers: The Mediating Effect of Physical Activity; 30155478	Jin, Y.; et al	2018	BioMed Research International	2018	Journal Article		Exclusion reason: Wrong study design (e.g., cross-sectional or qualitative)
Mindful2Work the next steps: Effectiveness of a program combining physical exercise, yoga and mindfulness, adding a wait-list period, measurements up to one year later and qualitative interviews	de Bruin E.I.; et al	2020	Complementary therapies in clinical practice	39	Journal Article	101137	Exclusion reason: Unable to group workers based on OPA;
Effects of a physical exercise intervention on employees' perceptions of quality of life: A randomized controlled trial	Brand R.; Schlicht W.; Grossmann K.; Duhnsen R.	2006	Sozial- und Praventivmedizin	51	1	14-23	Exclusion reason: Unable to group workers based on OPA;

A prospective study of the role of cardiovascular risk factors and fitness in industrial back pain complaints	Battie, M. C.; et al	1989	Spine	14	2	141-7	Exclusion reason: Did not examine effect of LTPA in OPA groups;
Work stress, sleep deficiency, and predicted 10-year cardiometabolic risk in a female patient care worker population	Jacobsen H.B.; et al	2014	American Journal of Industrial Medicine	57	8	940-949	Exclusion reason: No relevant health outcome;
Job burnout and depression: unraveling their temporal relationship and considering the role of physical activity	Toker S.; Biron M.	2012	The Journal of applied psychology	97	3	699-710	Exclusion reason: Did not examine effect of LTPA in OPA groups;
Yoga in the workplace and health outcomes: a systematic review	Puerto Valencia L.M.; et al	2019	Occupational medicine (Oxford, England)	69	3	195-203	Exclusion reason: Systematic review;
Health improved by physical activity intervention at work	Paton, Nic	2010	Occupational Health	62	10	7	Exclusion reason: Magazine article;
Exercise therapy and work-related musculoskeletal disorders in sedentary workers	Kelly, D.; et al	2018	Occupational medicine (Oxford, England)	68	4	262-272	Exclusion reason: Systematic review;
Interventions for the prevention of risk factors and incidence of type 2 diabetes in the work environment: a systematic review	Inolopu, Jorge; et al	2019	Revista de saude publica	53	Journal Article	101	Exclusion reason: Systematic review;
Physical activity and low back pain: A systematic review of recent literature	Heneweer H.; et al	2011	European Spine Journal	20	6	826-845	Exclusion reason: Systematic review;
Exercise treatment effect modifiers in persistent low back pain: an individual participant data meta-analysis of 3514 participants from 27 randomised controlled trials	Hayden J.A.; et al	2019	British journal of sports medicine		Journal Article		Exclusion reason: Systematic review;
Is sitting-while-at-work associated with low back pain? A systematic, critical literature review	Hartvigsen J.; et al	2000	Scandinavian Journal of Public Health	28	3	230-239	Exclusion reason: Systematic review;
The effects of workplace nature-based interventions on the mental	Gritzka S.; et al	2020	Frontiers in Psychiatry	11	Journal Article	323	Exclusion reason: Systematic review;

health and well-being of employees: A systematic review							
Association between sitting-time and all-cause mortality in the multiethnic cohort study	Kim Y.; et al	2011	American Journal of Epidemiology	173	Journal Article	S117	Exclusion reason: Did not examine effect of LTPA in OPA groups;
Is heavy physical work a risk factor for ischemic heart disease?	Makowiec-Dabrowska, Teresa	1995	Medycyna pracy	46	3	263-274	Exclusion reason: Not English, French, Danish, Norwegian or Dutch;
Mortality associated with physical activity in leisure time, at work, in sports, and cycling to work; 11924475	Andersen, L. B.; et al	2002	Ugeskrift for laeger	164	11	1501-1506	Exclusion reason: Unable to group workers based on OPA;
Health and Medicine - Diet and Nutrition; Studies from Technical University of Denmark Yield New Information about Diet and Nutrition (The impact of worksite interventions promoting healthier food and/or physical activity habits among employees working 'around the clock' hours: a ...)		2018	Obesity, Fitness & Wellness Week		Newspaper Article	5560	Exclusion reason: Magazine article;
The impact of five lifestyle factors on nurses' and midwives' health: The Australian and New Zealand nurses' and midwives' e-cohort study	Tuckett A.; Henwood T.	2015	International Journal of Health Promotion and Education	53	3	156-168	Exclusion reason: Wrong study design (e.g., cross-sectional or qualitative);
An effective physical fitness program for small and medium-sized enterprises	Tsai H.H.; et al Y.	2011	Industrial health	49	3	311-320	Exclusion reason: No relevant health outcome;
A randomised controlled trial to investigate walking 6,000 steps per day on pain and function in knee osteoarthritis progression: The walkout study	O'Hanlon M.S.; et al	2016	Osteoarthritis and Cartilage	24	Journal Article	S486	Exclusion reason: Chronic disease/condition population;
Do cardiovascular benefits of active travel vary by pre-existing risk	Laverty, Anthony A.	2018	The Lancet	392	Journal Article		Exclusion reason: Unable to group workers based on OPA;

factors? A longitudinal observational study							
Physical loading and performance as predictors of back pain in healthy adults. A 5-year prospective study	Kujala U.M.; et al	1996	European journal of applied physiology and occupational physiology	73	5	452-458	Exclusion reason: Did not examine effect of LTPA in OPA groups;
Association of physical activity at work with mortality in Israeli industrial employees: The CORDIS study	Kristal-Boneh E.; et al	2000	Journal of Occupational and Environmental Medicine	42	2	127-135	Exclusion reason: Did not examine effect of LTPA in OPA groups;
Television Watching and Other Sedentary Behaviors in Relation to Risk of Obesity and Type 2 Diabetes Mellitus in Women	Hu F.B.; et al	2003	Journal of the American Medical Association	289	14	1785-1791	Exclusion reason: Unable to group workers based on OPA;
Fitness, work, and leisure-Time physical activity and ischaemic heart disease and all-cause mortality among men with pre-existing cardiovascular disease	Holtermann A.; et al	2010	Scandinavian Journal of Work, Environment and Health, Supplement	36	5	366-372	Exclusion reason: Chronic disease/condition population;
Physical fitness and perceived psychological pressure at work: 30-year ischemic heart disease and all-cause mortality in the copenhagen male study	Holtermann A.; et al	2011	Journal of Occupational and Environmental Medicine	53	7	743-750	Exclusion reason: No leisure time PA or SB;
The relationship between leisure time, physical activities and musculoskeletal symptoms and disability in worker populations	Hildebrandt, V. H.; et al	2000	International archives of occupational and environmental health	73	8	507-18	Exclusion reason: Wrong study design (e.g., cross-sectional or qualitative);
Incidence of Type 2 diabetes among occupational classes in Sweden: a 35-year follow-up cohort study in middle-aged men	Heden Stahl, C.; et al	2014	Diabetic medicine : a journal of the British Diabetic Association	31	6	674-80	Exclusion reason: Did not examine effect of LTPA in OPA groups;
Predictors of occupational low back disability: implications for secondary prevention	Feuerstein, M.; Berkowitz, S. M.; Huang, G. D.	1999	Journal of occupational and environmental medicine	41	12	1024-31	Exclusion reason: Unable to group workers based on OPA;
Exploring the combined effect of job strain and occupational physical	Ferrario M.M.; et al	2018	Occupational and environmental medicine	75	Journal Article	A59-A60	Exclusion reason: No leisure time PA or SB;

activity on cardiovascular disease incidence							
Benefits of sport on cardiovascular risk in sedentary workers only	Ferrario M.M.	2018	Salud(i)Ciencia	23	3	264-266	Exclusion reason: Duplicate study;
Effect of combined occupational tasks on cardiovascular events: Prime study	Esquirol Y.; et al	2015	European heart journal	36	Journal Article	284	Exclusion reason: No leisure time PA or SB;
Do psychosocial job resources buffer the relation between physical work demands and coronary heart disease? A prospective study among men	Clays E.; et al	2016	International archives of occupational and environmental health	89	8	1299-1307	Exclusion reason: Did not examine effect of LTPA in OPA groups;
Effect of a musicians' exercise intervention on performance-related musculoskeletal disorders	Chan, Cliffton; et al	2014	Medical problems of performing artists	29	4	181-8	Exclusion reason: Intervention - ergonomic/productive work;
Bilingual Text4Walking Food Service Employee Intervention Pilot Study	Buchholz, Susan Weber; et al	2016	JMIR mHealth and uHealth	4	2	e68	Exclusion reason: No relevant health outcome;
Projected Effect of Increased Active Travel in German Urban Regions on the Risk of Type 2 Diabetes	Brinks, Ralph; et al	2015	PLoS One	10	4		Exclusion reason: Unable to group workers based on OPA;
Acute myocardial infarction in relation to physical activities at work: A nationwide follow-up study based on job-exposure matrices	Bonde J.P.E.; et al	2020	Scandinavian Journal of Work, Environment and Health	46	3	268-277	Exclusion reason: Did not examine effect of LTPA in OPA groups;
Five-year workplace wellness intervention in the NHS	Blake, Holly; et al	2013	Perspectives in public health	133	5	262-71	Exclusion reason: Unable to group workers based on OPA;
Death rates among the Italian railroad employees, with special reference to coronary heart disease and physical activity at work	Menotti, A.; Puddu, V.	1976	Environmental research	11	3	331-42	Exclusion reason: No leisure time PA or SB;
Ferrari Corporate Wellness Program: Results of a Pilot Analysis and the "Drag" Impact in the Workplace	Biffi A.; et al	2018	High Blood Pressure and Cardiovascular Prevention	25	3	261-266	Exclusion reason: Unable to group workers based on OPA;

Voluntary and occupational physical activity have different effects on mortality	Bahls M.; et al	2015	Circulation	132	Journal Article		Exclusion reason: Unable to group workers based on OPA;
Decreasing population blood pressure is not mediated by changes in habitual physical activity. Results from 15 years of follow-up	Andersen U.O.; Jensen G.	2007	Blood pressure	16	1	28-35	Exclusion reason: Unable to group workers based on OPA;
Effect of two contrasting types of physical exercise on chronic neck muscle pain	Andersen, Lars L.; et al	2008	Arthritis and Rheumatism	59	1	84-91	Exclusion reason: Unable to group workers based on OPA;
Perceived physical exertion during healthcare work and prognosis for recovery from long-term pain in different body regions: Prospective cohort study	Andersen L.L.; et al	2012	BMC Musculoskeletal Disorders	13	Journal Article	253	Exclusion reason: Did not examine effect of LTPA in OPA groups;
Effects of an Integrated Physical Activity Program for Physically Inactive Workers: Based on the PRECEDE-PROCEED Model	Kim H.J.; Choo J.	2018	Journal of Korean Academy of Nursing	48	6	692-707	Exclusion reason: Not English, French, Danish, Norwegian or Dutch;
Socioeconomic factors and physical activity in relation to cardiovascular disease and death. A 12 year follow up of participants in a population study of women in Gothenburg, Sweden	Lapidus L.; Bengtsson C.	1986	British heart journal	55	3	295-301	Exclusion reason: Did not examine effect of LTPA in OPA groups;
The prevalence of low physical activity in an urban population and its relationship with other cardiovascular risk factors: Findings of a community-based study (KERCADRS) in southeast of Iran	Najafipour H.; et al	2016	ARYA Atherosclerosis	12	5	212-219	Exclusion reason: Unable to group workers based on OPA;
Shift work, occupational noise and physical workload with ensuing development of blood pressure and their joint effect on the risk of coronary heart disease	Virkkunen, Hanna; et al	2007	Scandinavian journal of work, environment & health	33	6	425-34	Exclusion reason: No leisure time PA or SB;

Testing a workplace physical activity intervention: A cluster randomized controlled trial	McEachan, Rosemary R. C.; et al	2011	The International Journal of Behavioral Nutrition and Physical Activity	8	Journal Article		Exclusion reason: No relevant health outcome;
A systematic review of physical activity-based interventions in shift workers	Flahr H.; Brown W.J.; Kolbe-Alexander T.L.	2018	Preventive Medicine Reports	10	Journal Article	323-331	Exclusion reason: Systematic review;
Physical activity initiated by employer and its health effects; an eight week follow-up study	Skogstad M.; et al	2016	BMC public health	16	Journal Article	377	Exclusion reason: No relevant health outcome;
Health benefits of a pedometer-based physical activity intervention in sedentary workers	Chan C.B.; et al	2004	Preventive medicine	39	6	1215-1222	Exclusion reason: No relevant health outcome;
Effectiveness of physical training for self-employed persons with musculoskeletal disorders: A randomized controlled trial	Heinrich J.; et al	2009	BMC Public Health	9	Journal Article	200	Exclusion reason: Chronic disease/condition population;
The association between physical workload and low back pain clouded by the "healthy worker" effect: Population-based cross-sectional and 5-year prospective questionnaire study	Hartvigsen J.; et al	2001	Spine	26	16	1788-1792	Exclusion reason: No leisure time PA or SB;
Feasibility and impact of sit-stand workstations with and without exercise in office workers at risk of low back pain: A pilot comparative effectiveness trial	Johnston V.; et al	2019	Applied Ergonomics	76	Journal Article	82-89	Exclusion reason: Intervention - PA performed during work time;
Impact of body size and physical activity during adolescence and adult life on overall and cause-specific mortality in a large cohort study from Iran	Etemadi A.; et al	2014	European journal of epidemiology	29	2	95-109	Exclusion reason: No leisure time PA or SB;
A longitudinal analysis of the effects of a preventive exercise programme on the factors that predict shoulder pain in construction apprentices	Borstad, John D.; et al	2009	Ergonomics	52	2	232-44	Exclusion reason: Unable to group workers based on OPA;

Sitting occupations and physical intensity of work as predictors of mortality: A retrospective study of a population of workers in southern Italy	Caputi A.; et al	2020	Open Public Health Journal	13	1	75-79	Exclusion reason: Age <18 years >65 years;
[Physical exercise in the prevention of musculoskeletal diseases in the elderly worker]	Capodaglio, P.	2000	La Medicina del lavoro	91	4	379-86	Exclusion reason: Not English, French, Danish, Norwegian or Dutch;
Television viewing, computer use, time driving and all-cause mortality: The SUN cohort	Basterra-Gortari F.J.; et al	2014	Journal of the American Heart Association	3	3	864	Exclusion reason: Unable to group workers based on OPA;
0018 Do highly active workers die early? elucidating the physical activity health paradox in a systematic review with meta-analyses	Coenen, Pieter; et al	2017	Occupational and environmental medicine	74	Journal Article		Exclusion reason: Duplicate study;
Association among socioeconomic status, health behaviors, and all-cause mortality in the United States	Nandi A.; Glymour M.M.; Subramanian S.V.	2014	Epidemiology	25	2	170-177	Exclusion reason: Did not examine effect of LTPA in OPA groups;
Physical activity, fitness and body composition of Finnish police officers: A 15-year follow-up study	Sorensen L.; et al.	2000	Occupational Medicine	50	1	10-Mar	Exclusion reason: No relevant health outcome;
Occupational physical activity, energy expenditure and 11-year progression of carotid atherosclerosis	Krause N.; et al	2007	Scandinavian Journal of Work, Environment and Health	33	6	405-424	Exclusion reason: No leisure time PA or SB;
Physical work demands and physical fitness in low social classes	Holtermann A.; et al	2011	Occupational and environmental medicine	68	Journal Article	A53-A54	Exclusion reason: No leisure time PA or SB
Objectively measured physical activity and 12-month trajectories of neck-shoulder pain in workers: A prospective study in DPHACTO	Hallman D.M.; et al	2017	Scandinavian Journal of Public Health	45	3	288-298	Exclusion reason: Did not examine effect of LTPA in OPA groups;
Association between socioeconomic status, health behaviors and all-cause mortality in the United States	Nandi A.; Glymour M.M.; Subramanian S.V.	2012	European journal of epidemiology	27	1	S60-S61	Exclusion reason: Unable to group workers based on OPA;

A case-control study of physical activity patterns and risk of non-fatal myocardial infarction	Gong J.; et al	2013	BMC public health	13	Journal Article	122	Exclusion reason: Unable to group workers based on OPA;
Experience from a 10-year prospective prophylactic examination of the workers of a computer manufacturing factory	Mikunis R.I.; et al	1991	Terapevticheskii arkhiv	63	1	44-47	Exclusion reason: Not English, French, Danish, Norwegian or Dutch
'Walking to wellness' in an ageing sedentary university community-a randomised controlled feasibility study	Mackey M.; et al	2011	Physiotherapy (United Kingdom)	97	Journal Article	eS733-eS734	Exclusion reason: Study protocol;
Physical activity and common mental disorder: Results from the Caerphilly study	Wiles N.J.; et al	2007	American Journal of Epidemiology	165	8	946-954	Exclusion reason: Did not examine effect of LTPA in OPA groups;
Occupational and leisure time physical activity in contrasting relation to ambulatory blood pressure	Clays E.; et al	2012	BMC public health	12	Journal Article	1002	Exclusion reason: No relevant health outcome;
Chronic low back pain: a successful intervention for desk-bound workers	Dennerlein, Jack T.	2018	Occupational and environmental medicine	75	5	319-320	Exclusion reason: Commentary;
Physical activity level at work and risk of chronic low back pain: A follow-up in the Nord-Trondelag Health Study	Heuch, Ingrid; et al	2017	PloS one	12	4	e0175086	Exclusion reason: Did not examine effect of LTPA in OPA groups;
Active commuting reduces the risk of wrist fractures in middle-aged women - The UFO study	Englund U.; et al	2013	Osteoporosis International	24	2	533-540	Exclusion reason: Unable to group workers based on OPA;
Relationships between Certain Individual Characteristics and Occupational Injuries for Various Jobs in the Construction Industry: A Case-Control Study	Chau N.; Mur J.-M.; et al	2004	American Journal of Industrial Medicine	45	1	84-92	Exclusion reason: No relevant health outcome;
Worksite health promotion programs with environmental changes: A systematic review	Engbers L.H.; et al	2005	American Journal of Preventive Medicine	29	1	61-70	Exclusion reason: Systematic review;

Physical activity dose-response effects on outcomes of depression and anxiety	Dunn A.L.; Trivedi M.H.; O'Neal H.A.	2001	Medicine and science in sports and exercise	33	6	S587-S597	Exclusion reason: Systematic review;
The effectiveness of eHealth interventions on physical activity and measures of obesity among working-age women: a systematic review and meta-analysis	Cotie L.M.; et al	2018	Obesity Reviews	19	10	1340-1358	Exclusion reason: Systematic review;
Integrated Approaches to Occupational Health and Safety: A Systematic Review	Cooklin A.; et al	2017	American Journal of Health promotion : AJHP	31	5	401-412	Exclusion reason: Systematic review;
Interventions to reduce sedentary behavior and increase physical activity during productive work: A systematic review	Commissaris D.A.C.M.; et al	2016	Scandinavian Journal of Work, Environment and Health	42	3	181-191	Exclusion reason: Systematic review;
Tai Chi and Workplace Wellness for Health Care Workers: A Systematic Review	Cocchiara, Rosario Andrea; et al	2020	International journal of environmental research and public health	17	1		Exclusion reason: Systematic review;
Effectiveness of workplace diabetes prevention programs: A systematic review of the evidence	Brown, Sharon A.; et al	2018	Patient education and counseling	101	6	1036-1050	Exclusion reason: Systematic review;
Sedentary lifestyle as a risk factor for low back pain: A systematic review	Chen S.-M.; et al	2009	International archives of occupational and environmental health	82	7	797-806	Exclusion reason: Systematic review;
Are workplace interventions to reduce sitting effective? A systematic review	Chau J.Y.; et al	2010	Preventive medicine	51	5	352-356	Exclusion reason: Systematic review;
Green exercise as a workplace intervention to reduce job stress. Results from a pilot study	Calogiuri, Giovanna; et al	2015	Work (Reading, Mass.)	53	1	99-111	Exclusion reason: No relevant health outcome;
Relation Between Leisure Time, Commuting, and Occupational Physical Activity With Blood Pressure in 125 402 Adults: The Lifelines Cohort	Byambasukh O.; Snieder H.; Corpeleijn E.	2020	Journal of the American Heart Association	9	4	e014313	Exclusion reason: Unable to group workers based on OPA;

It's Time to Start Changing the Game: A 12-Week Workplace Team Sport Intervention Study	Brinkley, Andrew; et al	2017	Sports medicine - open	3	1	30	Exclusion reason: No relevant health outcome;
Get moving: A web site that increases physical activity of sedentary employees; 21192750	Blair Irvine, A.; et al	2011	American Journal of Health Promotion	25	3	199-206	Exclusion reason: Unable to group workers based on OPA;
Effect of two choreographed fitness group-workouts on the body composition, cardiovascular and metabolic health of sedentary female workers	Barranco-Ruiz Y.; et al	2019	International Journal of Environmental Research and Public Health	16	24	4986	Exclusion reason: No relevant health outcome;
Impact of lifestyle intervention on physical activity and diet of Japanese workers	Arao T.; et al	2007	Preventive medicine	45	03-Feb	146-152	Exclusion reason: No relevant health outcome;
Psychosocial effects of workplace physical exercise among workers with chronic pain randomized controlled trial	Andersen L.L.; et al	2017	Medicine (United States)	96	1	5709	Exclusion reason: No relevant health outcome;
Leisure time but not occupational physical activity significantly affects cardiovascular risk factors in an adult population; 17976196	Sofi, F.; et al	2007	European journal of clinical investigation	37	12	947-953	Exclusion reason: No relevant health outcome;
Leisure time physical activity in a 22-year follow-up among Finnish adults	Borodulin K.; et al	2012	International Journal of Behavioral Nutrition and Physical Activity	9	Journal Article	121	Exclusion reason: No relevant health outcome;
The (cost-)effectiveness of a lifestyle physical activity intervention in addition to a work style intervention on the recovery from neck and upper limb symptoms in computer workers	Bernaards, Claire M.; et al	2006	BMC musculoskeletal disorders	7	Journal Article	80	Exclusion reason: Study protocol
Recreational and occupational physical activities as risk factors for cardiovascular disease	Kawada T.	2013	International journal of cardiology	165	3	559-560	Exclusion reason: Commentary
Effect of physical exercise interventions on musculoskeletal pain in all body regions among office	Andersen L.L.; et al	2010	Manual therapy	15	1	100-104	Exclusion reason: Intervention - PA

workers: A one-year randomized controlled trial							performed during work time;
Occupational but not leisure-time physical activity is associated with increased risk of atrial fibrillation, a prospective observational study of 15,818 healthy subjects	Skielboe A.K.; et al	2015	European Journal of Preventive Cardiology	22	1	S5	Exclusion reason: Did not examine effect of LTPA in OPA groups;
Television viewing time, physical activity and low back pain in community-based adults: Results from a prospective cohort study	Hussain S.M.; et al	2016	Annals of the Rheumatic Diseases	75	Journal Article	853	Exclusion reason: Not working population
Trends in active transportation and associations with cardiovascular disease risk factors among U.S. adults, 2007â€“2016; 30227156	Zwald, M. L.; et al	2018	Preventive medicine	116	Journal Article	150-156	Exclusion reason: Wrong study design (e.g., cross-sectional or qualitative);
Occupational versus leisure-time physical activity in reducing cardiovascular risks and mortality among ethnic Chinese adults in Taiwan	Hu G.-C.; et al	2014	Asia-Pacific Journal of Public Health / Asia-Pacific Academic Consortium for Public Health	26	6	604-613	Exclusion reason: Did not examine effect of LTPA in OPA groups;
Weak associations between occupational physical activity and myocardial infarction	Johnsen A.; et al	2016	Occupational and environmental medicine	73	Journal Article	A197-A198	Exclusion reason: No leisure time PA or SB
Sitting occupations are an independent risk factor for Ischemic stroke in North Indian population	Kumar A.; Prasad M.; Kathuria P.	2014	International Journal of Neuroscience	124	10	748-754	Exclusion reason: No leisure time PA or SB;
Patterns of physical activity and the risk of coronary heart disease: A pilot study	Al-Zoughool M.; Al-Ahmari H.; Khan A.	2018	International Journal of Environmental Research and Public Health	15	4	778	Exclusion reason: Unable to group workers based on OPA;
Effects of a Health-Partner Intervention on Cardiovascular Risk	Al Mheid, Ibhar; et al	2016	Journal of the American Heart Association	5	10		Exclusion reason: Unable to group workers based on OPA;
Do highly physically active workers die early? A systematic review with meta-analysis of data from 193 696 participants	Coenen P.; et al	2018	British journal of sports medicine	52	20	1320-1326	Exclusion reason: Systematic review;

Do workplace physical activity interventions improve mental health outcomes?	Chu A.H.; et al.	2014	Occupational medicine (Oxford, England)	64	4	235-245	Exclusion reason: Systematic review;

Supplemental table 3. Study characteristics table for studies examining effects of OPA and LTPA by health outcome

First author, year	Country, cohort	Population description	OPA		Age mean (SD), range in years	Study design	Sample size analyzed			Length of follow-up	LTPA		Outcome assessment
			Classification method	OPA groups			Total N	Men N (%)	Women N (%)		Assessment/ intervention	LTPA groups	
**Diabetes incidence**													
Biswas, 2020 [33]	Canada, Canadian Community Health Survey	Workers from population sample	Occupation title, JEMS	1. Sitters 2. Intermittent movers 3. Heavy labour	35-74	PCS	7,026	3,704 (52%)	3,322 (47%)	Median = 13.7 years	Survey LTPA items	Low (15-150 min/week of MVPA) vs. high (≥150 min/week of MVPA)	Hospital discharges and laboratory claims
**Metabolic syndrome**													
Kuwahara, 2016 [52]	Japan, J-ECOH Study	Manufacturing	Single OPA question	1. Sitters (sedentary) 2. Heavy labour (active)	45.2 (7.9), 30-64	PCS	22,383	18,992 (84%)	3,391 (16%)	Mean = 4.1 years	SR usual LTPA levels	No exercise (0 MET-h/week), low (>0 to 7.5 MET-h/week), medium (7.5 to 16.5 MET-h/week), high (16.5 to 25.5 MET-h/week), or very high (>25.5 MET-h/week)	Medical records
**Musculoskeletal pain**													
Haukka, 2012 [40] *MSKP	Finland, None	Kitchen workers	Occupation title	Intermittent movers	47, 21-62	PCS	289	0 (0%)	289 (100%)	2 years	Single Q about past 12-month exercise frequency	Exercise frequency classified using tertiles: ≤1/week (low), 2-3/ week (moderate) and ≥4/week (high)	SR MSKP Index
Holtermann, 2009 [43] *MSKP	Denmark, None	Cleaners	Occupation title	Heavy labour	49.5 (16.9)	CC	141; 83 cases, 25 controls	0 (0%)	141 (100%)	NR	Saltin & Grimby PA questions	Level 1 (almost physically inactive or LPA <2 h/week), Level 2 (LPA 2–4 h/week), Level 3 (LPA >4 h/week or more strenuous PA 2–4 h/week), Level 4 (more VPA >4 h/week or regular hard workouts and perhaps sport competitions, several times per week)	Modified Nordic questionnaire to SR MSK symptoms
Jakobsen, 2015 [50] *MSKP	Denmark, None	Health care workers (general)	Occupation title	heavy labour	18-67	RCT	200: 111 (I at home), 89 (C at work)	0 (0%)	200 (100%)	10 weeks	Intervention adherence / Strength training 5 x 10 minutes/week	Intervention vs. control	SR MSKP using a modified Nordic questionnaire
Korhonen, 2003 [51] *Neck pain	Finland, None	Municipal administrative workers	Occupation title	Sitters	47, 25-61	PCS	180	100 (56%)	80 (44%)	12 months	Frequency questionnaire	≥2 x week vs. ≤1 x week	Single question about radiating neck pain for

First author, year	Country, cohort	Population description	OPA		Age mean (SD), range in years	Study design	Sample size analyzed			Length of follow-up	LTPA		Outcome assessment
			Classification method	OPA groups			Total N	Men N (%)	Women N (%)		Assessment/ intervention	LTPA groups	
													at least 8 days
Miranda, 2001 [53]<br><br>*Shoulder pain	Finland, None	Employees of a forestry company	OPA questionnaire	Heavy labourers	White collar: 45.3 (9.2), blue collar: 45.3 (9.1)	PCS	2,094	NR	NR	1 year	Questionnaire with multiple questions re: LTPA	NR	SR: modified Nordic questionnaire
Pedersen, 2013 [54]<br><br>*Back, neck and upper extremity pain	Denmark, None	Industrial laboratory technicians	Occupation title	Sitters	First training group (TG1) = 42 (10); second training group (TG2) = 42 (11), 18-67	RCT	537, TG1: 282 (I), 255 (C), TG2: 255 (I), 211 (C)	82 (15%), TG1 = 51 (20%), TG2 = 31 (11%)	455 (85%), TG1 = 204 (80%), TG2 = 251 (89%)	20 weeks & 1 year	Intervention adherence / Strength training for 20 min 3 x week	Intervention vs. control	Disability of the Arm Shoulder and Hand (DASH) questionnaire
Riihimaki, 1994 [56,56]<br><br>*Sciatic pain	Finland, None	Office workers, machine operators, carpenters	Occupation title	1. Sitters<br>2. Standers<br>3. Heavy labourers	25-49	PCS	674 office workers, 852 machine operators, 696 carpenters	100%	0%	3 years	Recreation/ sport frequency questionnaire	Maximum 1x week vs. >1 x week	SR sciatic pain
Sihawong, 2014 [58]<br><br>*LBP	Thailand, None	Office workers	Occupation title	Sitters	I = 37.3 (10.1); C = 36.9 (10.7)<br><br>18-55	RCT	563: 282 (I), 281 (C)	I = 99 (34.9%) C = 77 (27.2%)	I = 183 (65.1%) C = 204 (72.8%)	12 months	Intervention adherence / muscle strengthening & endurance training 2 x week	Intervention vs. control	SR, pain intensity measured by VAS
Sihawong, 2014b [59]<br><br>*Neck pain	Thailand, None	Office workers	Occupation title	Sitters	I = 37.2 (10.1), C = 36.9 (10.7)<br><br>18-55	RCT	567; 285 (I), 282 (C)	I = 150 (52.4%), C = 135 (47.6%)	I = 135 (47.6%), C = 147 (52.4%)	12 months	Intervention adherence / muscle strengthening & endurance training 2 x week	Intervention vs. control	SR, pain intensity measured by VAS
Van den Heuvel, 2005 [62]<br><br>*Neck and upper limb pain	Netherlands, SMASH study	Employees of 34 different companies	Study OPA questionnaire	Sedentary work (sitters) vs. more active work	Mean age between 35 and 38	PCS	1,742	632 (70%)	268 (30%)	3 years	SR sport participation and active transportation	Sporting activities: <1h/week, 1-2h/week, ≥3h/week<br><br>Active transport: None, 10-150 min/week, ≥150 min/week	SR modified Nordic questionnaire
Yip, 2004 [66]<br><br>*LBP	Hong Kong, None	Nurses	Occupation title	Heavy labourers	30.7 and 31.4 for nurses who did and did not develop LBP during follow-up	PCS	144	21 (15%)	123 (85%)	12 months	Study specific questionnaire	Sedentary (no exercise), underactive (3+ /week exercise of MPA), active (3+ /week exercise at MVPA)	SR occurrence of any LBP using study-specific questionnaire

First author, year	Country, cohort	Population description	OPA		Age mean (SD), range in years	Study design	Sample size analyzed			Length of follow-up	LTPA		Outcome assessment
			Classification method	OPA groups			Total N	Men N (%)	Women N (%)		Assessment/ intervention	LTPA groups	
**Depression**													
Bernaards, 2006 [32]	Netherlands, SMASH	Workers in blue-collar jobs, white-collar jobs, and caring professions	Single Q	Sitters vs. non-sitters	NR	PCS	1,401	NR	NR	~3 years	Single Q on frequency per month	4 LTPA groups: <1 per month of strenuous PA; 1–3 times per month; 1–2 times per week; and, >3 times per week	SR using the CES-D
**CVD & IHD Incidence**													
Allesoe, 2015 [30]<br><br>*IHD	Denmark, Danish Nurse Cohort Study	Nurses	Saltin & Grimby PA questionnaire	1. Sitters<br>2. Standers<br>3. Intermittent movers<br>4. Heavy labourers	Median = 51, 45-64	PCS	12,093	0, 0%	12,093, 100%	15 years	Saltin & Grimby	Sedentary (mostly sedentary activities), moderate (walk, bicycle or LPA) vs. vigorous (athlete, heavy housework or vigorous training 4+ hours/week)	Medical records
Clays, 2013 [34]<br><br>*CHD	Belgium, BELSTRESS cohort	NR	Job Content Questionnaire	Low vs. high OPA	45.8 (6.0), 35-59	PCS	14,337	14,337 (100%)	0 (0%)	Median = 3.15 years	Study-specific questionnaire	Low (none to low PA) vs. moderate-high (20+ min at least 1 x week)	NR
Ferrario, 2018 [37]<br><br>*CHD events	Italy, MONICA Brianza surveys II and III; PAMELA, SEMM	Manufacturing, factory workers, executive and white- and blue-collar workers	Baecke Questionnaire	Low, medium, high OPA	Low OPA = 42.8 (9.1), mid OPA = 39.9 (8.9), high OPA = 39.6 (9.2)	PCS	3,574	3,574 (100%)	0 (0%)	Median = 14 years	Baecke Questionnaire	Tertiles, with cut-offs at 2.5 and 3.125 points (on a scale ranging from 1 to 5).	Death registry, hospital records
Fransson, 2004 [38]<br><br>*MI	Sweden, SHEEP	NR	Occupational sitting & PA questionnaire	1. Sitters<br>2. Heavy labourers	Men: cases = 59.0 (7.1), controls = 59.2 (7.2); Women: cases = 62.0 (6.6); controls = 62.1 (6.7)	CC	4,069	2,742 (67%)	1,327 (33%)	NR	Study-specific frequency questionnaire	Rarely, Occasional walks (1 time per week), Sometimes (2-3 times per week), Regularly (>3 times per week)	Death registry, medical records
Holtermann, 2012b [45]<br><br>*MI	Denmark, CCHS	NR	Saltin & Grimby questionnaire	1. Sitters<br>2. Intermittent movers<br>3. Heavy labourers	Men: low OPA = 52.1 (10.2), medium OPA = 53.0 (9.4), high OPA = 51.5 (8.6); Women: low OPA = 54.1 (9.1), medium	PCS	7,819	3,281 (42%)	4,538 (58%)	17.8 years	Single question	Low (almost entirely sedentary, LPA <2 h/week), medium (LPA 2-4 h/week), high (LPA >4 h/week or MVPA >2 h/week)	Medical records, death registry

First author, year	Country, cohort	Population description	OPA		Age mean (SD), range in years	Study design	Sample size analyzed			Length of follow-up	LTPA		Outcome assessment
			Classification method	OPA groups			Total N	Men N (%)	Women N (%)		Assessment/ intervention	LTPA groups	
					OPA = 54.4 (9.0), high OPA = 49.7 (9.1), 20-67								
Hu, 2005 [48]<br><br>*Stroke	Finland, None	NR	OPA questionnaire	1. Sitters (light OPA)<br>2. Intermittent movers (moderate OPA)<br>3. Heavy labourers (active OPA)	25-64	PCS	47,721	22,841 (48%)	24,880 (52%)	Mean = 19.0 years	Questions on usual LTPA per week and active transportation per week	LTPA: light (almost completely inactive); moderate (some PA for 4 h/week); high (VPA for >3 h/week)<br><br>Active transportation: (1) motorized transport or no work; (2) walking or cycling 1-29 min/day; (3) walking or cycling for ≥30 min/day	Medical records, death registry
Hu, 2007 [49]<br><br>*CHD	Finland, None	NR	OPA questionnaire	1. Sitters (low OPA)<br>2. Intermittent movers (moderate OPA)<br>3. Heavy labourers (high OPA)	Men: without CHD = 42.4, with CHD = 49.2 Women: with CHD = 43.4, without CHD = 53.8, 25-64	PCS	47,840	22,877 (48%)	24,963 (52%)	18.9 years	Questions on usual LTPA per week and active transportation per week	LTPA: light (almost completely inactive); moderate (some PA for 4 h/week); high (VPA for >3 h/week)<br><br>Active transportation: (1) motorized transport or no work; (2) walking or cycling 1-29 min/day; (3) walking or cycling for ≥30 min/day	Medical records, death registry
Petersen, 2012 [10]<br><br>*IHD	Denmark, DNHIS	Random sample of working population	Question on occupational heavy lifting	1. Heavy labourers (heavy lifting)<br>2. Sedentary + intermittent (no heavy lifting)	18-65	PCS	12,508	6,659 (53%)	5,850 (47%)	Unclear, max 21 years	Self-report questionnaire	Low (sedentary and LPA) vs. high (MVPA)	Medical records
Wang, 2010 [65]<br><br>*HF	Finland, Seven population-based cohorts	General population	Study specific OPA questions	1. Sitters<br>2. Intermittent movers<br>3. Heavy labourers	25-74	PCS	58,208	28,334 (49%)	29,874 (51%)	Mean = 18.4 years	SR questions on frequency of sport and active travel	LTPA: light (almost completely inactive); moderate (some PA for 4 h/week); high (VPA for >3 h/week)<br><br>Active transportation: (1) motorized transport or no work; (2) walking or	Medical records & death registry

First author, year	Country, cohort	Population description	OPA		Age mean (SD), range in years	Study design	Sample size analyzed			Length of follow-up	LTPA		Outcome assessment
			Classification method	OPA groups			Total N	Men N (%)	Women N (%)		Assessment/ intervention	LTPA groups	
												cycling 1-29 min/day; (3) walking or cycling for ≥30 min/day	
Wang, 2016 [63]<br><br>*MI	Finland, Kuopio Ischemic Heart Disease Risk Factor (KIHD) Study	NR	OPA questionnaire and assessment of relative aerobic strain (RAS)	1. Sitters<br>2. Intermittent movers<br><br>Low (<33% RAS) vs. high OPA (>33% RAS)	Without IHD - 51.5 (5.0); with HD - 53.5 (3.9), 42-60	PCS	1,891	1,891 (100%)	0 (0%)	20 years	Study 12-month LTPA history (frequency of recreation/sport PA)	Low (<75 min/week) vs. high (>75 min/week)	SR, medical records, death registry, hospital discharge records
Wang, 2019 [64]<br><br>*MI or CHD death	United States, Women's Health Initiative Observational Study	Office workers, teachers, health care workers (general), law enforcement	Occupation title, Occupational Information Network database	1. Sitters (low)<br>2. Heavy labourers (very high)<br><br>Low, moderate, high and very high	63.4 (7.2), 50-79	CC	Cumulative OPA = 8307; Most recent OPA = 8,057<br><br>Cases = 3,422, controls = 5,243	0%	100%	11.4 years	WHI PA questionnaire	Low vs. high (unclear how this was derived)	SR, death registry
**Arrhythmias**													
Skielboe, 2016 [60]	Denmark, CCHS	General population	Study questionnaire	1. Sitters<br>2. Intermittent movers<br>3. Heavy labourers x 2	20-93	PCS	17,196	7,258 (42%)	9,938 (58%)	20.3 years	Study PA questions	Low, moderate, high and very high	Objectively assessed by ECG, AF registered in patient registry or death registry
**CVD & IHD mortality**													
Barengo, 2004 [31]	Finland, North Karelia Project, FINMONICA /Finrisk	General population	SR Questionnaire	1. Sitters<br>2. Intermittent movers<br>3. Heavy labourers	Men: 43.4 (8.4), women: 43.8 (8.5)	PCS	32,677	15,853, 48.5%	16,824, 51.5%	Median = 20 years	General PA questionnaire	High (participation in recreational sports or in intense training or sports competitions for ≥3 h/week), Moderate (walking, cycling or practising some other form of light exercise ≥4 h/week), Low (reading, watching TV or working in the household without much PA)	Death registry
Fan, 2019 [36]	China, China Kadoorie Biobank	Factory and non-factory workers	Occupation title	Factory (standers) vs. non-factory workers	45.9	PCS	104,170	51.4%	48.6%	Median = 10 years	SR frequency of active travel over past year	No active commuting, working at/near home, walking to work, cycling to work	Medical records, death registry, disease registries.

First author, year	Country, cohort	Population description	OPA		Age mean (SD), range in years	Study design	Sample size analyzed			Length of follow-up	LTPA		Outcome assessment
			Classification method	OPA groups			Total N	Men N (%)	Women N (%)		Assessment/ intervention	LTPA groups	
													health insurance system
Fransson, 2004 [38]	Sweden, SHEEP	NR	Occupational sitting & PA questionnaire	1. Sitters<br>2. Heavy labourers x 2	Men: cases = 59.0 (7.1), controls = 59.2 (7.2); Women: cases = 62.0 (6.6); controls = 62.1 (6.7)	CC	4,069	2,742 (67%)	1,327 (33%)	NR	Single study-specific question	High (recreational sports or intense sports competition ≥3 h/week) vs. moderate (walking, cycling or other forms of light exercise ≥4 h/week) vs. low (largely inactive)	Death registry
Harari, 2015 [39]	Israel, CORDIS study	Manufacturing, machine operators	OPA questionnaire	None-mild vs. moderate-hard OPA	42.1 (12.1), 20-70	PCS	4,819	4,819 (100%)	0 (0%)	22 years	Study specific question	LTPA (≥2 x week for ≥30 mins) vs. none	Death registry
Holme, 1981 [42]	Norway, Oslo Study	NR	OPA questionnaire	Sedentary, Moderate, Intermediate, Great (not described)	40-49	PCS	14,701	14,701 (100%)	0 (0%)	4.5 years	Study specific questionnaire	Sedentary, Moderate, Intermediate, Great (not described)	Death registry
Holtermann, 2009 [44]	Denmark, Copenhagen Male Study	Construction workers, military, railway, postal and telephone services, customs, national banking, and medical industries	OPA questionnaire	1. Sitters<br>2. Intermittent movers<br>3. Heavy labourers	Low OPA = 48.7 (5.3), moderate OPA = 48.6 (5.3), high OPA = 48.7 (5.2), 40-59	PCS	4,876	4,876 (100%)	0 (0%)	30 years	Single study-specific question	High (recreational sports or intense sports competition ≥3 h/week) vs. moderate (walking, cycling or other forms of light exercise ≥4 h/week) vs. low (largely inactive)	Death registry
Holtermann, 2012a [46]	Denmark, Copenhagen Male Study	14 companies covering the railway, public road construction, military, post, telephone, customs, national bank and the medical industry	OPA questionnaire	1. Sitters<br>2. Intermittent movers<br>3. Heavy labourers x 2	40-59	PCS	5,249	5,249 (100%)	0 (0%)	30 years	Single study-specific question	High (LPA > 4 h/week or MVPA >2 h/week) vs. moderate (LPA 2-4 h/week) vs. low (largely inactive, LPA <2 h/week)	Death registry
Holtermann, 2013 [100]	Denmark, CCHS	General population	Saltin & Grimby OPA question	Men:<br>1. Sitters<br>2. Intermittent movers<br>3. Heavy labourers **x 2**	Males: low OPA = 44.8 (12.7), moderate OPA = 47.6 (11.4), high	PCS	16,237	7,411 (45.4%)	8,916 (54.6%)	22.4 years	Saltin & Grimby LTPA question	High (LPA > 4 h/week or MVPA >2 h/week) vs. moderate (LPA 2-4 h/week) vs. low (largely inactive, LPA <2 h/week)	Death registry

First author, year	Country, cohort	Population description	OPA		Age mean (SD), range in years	Study design	Sample size analyzed			Length of follow-up	LTPA		Outcome assessment
			Classification method	OPA groups			Total N	Men N (%)	Women N (%)		Assessment/ intervention	LTPA groups	
				Women: 1. Sitters 2. Intermittent movers 3. Heavy labourers	OPA = 47.4 (11.1), very high OPA = 46.7 (10.8). Females: low OPA = 45.2 (12.5), moderate OPA = 49.5 (11.0), high OPA = 44.1 (11.1), 20-67								
Salonen, 1988 [57]	Finland, North Karelia Project	General working population	Single OPA question	Active workers vs. sedentary (sitters)	30-59	PCS	15,088	NR	NR	6 years	General LTPA question	Low (<4 h/week) vs. high (≥4 h/week)	Death registry
Stamatakis, 2013 [61]	United Kingdom, HSE and two SHS	General working population	Single OPA question	Sitting vs. non-sitting occupations	≥ 40	PCS	11,168	5,788 (52%)	5,380 (48%)	12.9 years	Study LTPA question	Low vs. high LTPA based on sex-specific median	Death registry
**All-cause mortality**													
Barengo, 2004 [31]	Finland, North Karelia Project, FINMONICA / Finrisk	General population	PA Questionnaire	1. Sitters 2. Intermittent movers 3. Heavy labourers	Men: 43.4 (8.4), women: 43.8 (8.5)	PCS	32,677	15,853, 48.5%	16,824, 51.5%	Median = 20 years	General PA questionnaire	High (participation in recreational sports or in intense training or sports competitions for ≥3 h/week), Moderate (walking, cycling or practising some other form of light exercise ≥4 h/week), Low (reading, watching TV or working in the household without much PA)	Death registry
Clays, 2014 [35]	Belgium, Belgian Physical Fitness Study	NR	Study-specific PA questionnaire	Low (first tertile) vs. high (second and third tertiles)	46.3 (4.2), 40-55	PCS	1,456	1,456 (100%)	0 (0%)	Mean = 16.9 years	Minnesota LTPA Questionnaire	Low (bottom tertile) vs. high (top two tertiles)	Death registry
Harari, 2015 [39]	Israel, CORDIS study	Manufacturing, machine operators	OPA questionnaire	None-mild OPA vs. moderate-hard OPA	42.1 (12.1), 20-70	PCS	4,819	4,819 (100%)	0 (0%)	22 years	SR times/week, duration and type of LTPA	LTPA (≥2 x week for ≥30 mins/time) vs. none	Death registry
Hermansen, 2019 [41]	Norway, Finnmark Study	NR	OPA questionnaire	1. Sitters 2. Intermittent movers 3. Heavy labourers x 2	47.2 (9.2)	PCS	17,697	8,951 (50.6%)	8,746 (49.4%)	23.3 years	Saltin & Grimby PA questionnaire	'Inactive', 'moderate' (walking, bicycling, etc. ≥4 h/week) and 'active' (recreational sports etc. ≥4 h/week or hard training or	Death registry

First author, year	Country, cohort	Population description	OPA		Age mean (SD), range in years	Study design	Sample size analyzed			Length of follow-up	LTPA		Outcome assessment
			Classification method	OPA groups			Total N	Men N (%)	Women N (%)		Assessment/ intervention	LTPA groups	
												competitions several times a week).	
Holme, 1981 [42]	Norway, Oslo Study	NR	OPA questionnaire	Sedentary, Moderate, Intermediate, Great (not described)	40-49	PCS	14,701	14,701 (100%)	0 (0%)	4.5 years	Study specific questionnaire	Sedentary, Moderate, Intermediate, Great (not described)	Death registry
Holtermann, 2009 [44]	Denmark, Copenhagen Male Study	Construction workers, military, railway, postal and telephone services, customs, national banking, and medical industries	OPA questionnaire	1. Sitters<br>2. Intermittent movers<br>3. Heavy labourers	Low OPA = 48.7 (5.3), moderate OPA = 48.6 (5.3), high OPA = 48.7 (5.2), 40-59	PCS	4,876	4,876 (100%)	0 (0%)	30 years	Single study-specific question	High (recreational sports or intense sports competition ≥3 h/week) vs. moderate (walking, cycling or other forms of light exercise ≥4 h/week) vs. low (largely inactive)	Death registry
Holtermann, 2012a [46]	Denmark, Copenhagen Male Study	14 companies covering the railway, public road construction, military, post, telephone, customs, national bank and the medical industry	OPA questionnaire	1. Sitters<br>2. Intermittent movers<br>3. Heavy labourers **x 2**	40-59	PCS	5,249	5,249 (100%)	0 (0%)	NR	Single study-specific question	High (LPA > 4 h/week or vigorous >2 h/week) vs. moderate (LPA 2-4 h/week) vs. low (largely inactive, LPA <2 h/week)	Death registry
Holtermann, 2012b [45]	Denmark, CCHS	NR	Saltin & Grimby questionnaire	1. Sitters<br>2. Intermittent movers<br>3. Heavy labourers	Men: low OPA = 52.1 (10.2), medium OPA = 53.0 (9.4), high OPA = 51.5 (8.6); Women: low OPA = 54.1 (9.1), medium OPA = 54.4 (9.0), high OPA = 49.7 (9.1); All: 25-66	PCS	7,819	3,281 (42%)	4,538 (58%)	17.8 years	Single question	Low (almost entirely sedentary, LPA <2 h/week), medium (LPA 2-4 h/week), high (LPA >4 h/week or MVPA >2 h/week)	Medical records, death registry
Holtermann, 2013 [100]	Denmark, CCHS	General population	Saltin & Grimby OPA question	Men:<br>1. Sitters	Males: Low OPA = 44.8 (12.7),	PCS	16,237	7,411 (45.4%)	8,916 (54.6%)	22.4 years	Saltin & Grimby LTPA question	High (LPA > 4 h/week or MVPA >2 h/week) vs. moderate	Death registry

First author, year	Country, cohort	Population description	OPA		Age mean (SD), range in years	Study design	Sample size analyzed			Length of follow-up	LTPA		Outcome assessment
			Classification method	OPA groups			Total N	Men N (%)	Women N (%)		Assessment/ intervention	LTPA groups	
				2. Intermittent movers<br>3. Heavy labourers **x 2**<br>Women:<br>1. Sitters<br>2. Intermittent movers<br>3. Heavy labourers	moderate OPA = 47.6 (11.4), high OPA = 47.4 (11.1), very high OPA = 46.7 (10.8)<br><br>Females: Low OPA = 45.2 (12.5), moderate OPA = 49.5 (11.0), high OPA = 44.1 (11.1), 20-67							(LPA 2-4 h/week) vs. low (largely inactive, LPA <2 h/week)	
Petersen, 2012 [10]	Denmark, DNHIS	Random sample of working population	Question on occupational heavy lifting	Heavy lifting vs. no heavy lifting	18-65	PCS	12,508	6,659 (53%)	5,850 (47%)	Unclear, max 21 years	Self-report questionnaire	Low (sedentary and LPA) vs. high (MVPA)	Death registry
Stamatakis, 2013 [61]	United Kingdom, HSE and two SHS	General working population	Single OPA question	Sitting vs. non-sitting occupations	≥ 40	PCS	11,168	5,788 (52%)	5,380 (48%)	12.9 years	Study LTPA question	Low vs. high LTPA based on sex-specific median	Death registry

C – control group, CC – case control, CCHS – Copenhagen City Heart Study, CES-D – Centre for Epidemiological Studies Depression questionnaire, CHD – coronary heart disease, DNHIS – Danish National Health Interview Surveys, DM – device-measured, HADS – Hospital Anxiety and Depression Scale, HF – heart failure, HSE – Health Survey for England, I – intervention group, IHD – ischemic heart disease, J-ECOH – Japan Epidemiology Collaboration on Occupational Health Study, LBP – low back pain, LPA – light intensity physical activity, LTPA – leisure-time physical activity, MI – myocardial infraction, MONICA – Multinational Monitoring of Trends and Determinants in Cardiovascular Disease, MPA – moderate-intensity physical activity, MSKP – musculoskeletal pain, MVPA – moderate-to-vigorous intensity physical activity, NR – not reported, OPA – occupational physical activity, PA – physical activity, PAMELA – Pressioni Arteriose Monitorate e Loro Associazioni, PCS – prospective cohort study, SEMM – Study of Employed in the Municipality of Milan, SHEEP – Stockholm Heart Epidemiology Program, SHS – Scottish Health Survey, SMASH – Study on Musculoskeletal disorders, Absenteeism, Stress and Health, SR – self-report, VAS – visual analogue scale, VPA – vigorous intensity physical activity

Supplemental table 4. Findings table for studies examining effects of OPA and LTPA by health outcome

<table>
<tr><th rowspan="2">First author, year</th><th rowspan="2">OPA groups</th><th rowspan="2">Model, covariates</th><th colspan="3">Effect estimates</th><th rowspan="2">Narrative synthesis of findings</th></tr>
<tr><th>Combined men and women</th><th>Men</th><th>Women</th></tr>
<tr><td colspan="7">Diabetes incidence</td></tr>
<tr><td>Biswas, 2020</td><td>Sitters
Intermittent movers
Heavy labour</td><td>Model: cox proportional hazards

Covariates: age, sex, education, smoking, BMI, marital status, cohabitation with children <12 years, born outside of Canada, ethnicity, urban/rural residence, chronic medication conditions, other chronic conditions, presence of a long-term physical or mental health condition that limited activity performed at work, usual hours working/ week, weeks worked in past 12 months, shift work schedule, fruit & vegetable, alcohol</td><td><u>Analysis #1:</u>
Ref = low OPA/stationary + low LTPA vs.
Low OPA/stationary + high LTPA
HR = 0.63, 95% CI: 0.48, 0.84
Low OPA/movement + low LTPA
HR = 0.95, 95% CI: 0.66, 1.35
Low OPA/movement + high LTPA
HR = 0.95, 95% CI: 0.62, 1.46
High OPA + low LTPA
HR = 0.83, 95% CI: 0.61, 1.14
High OPA + high LTPA
HR = 0.80, 95% CI: 0.57, 1.12

<u>Analysis #2:</u>
Ref = Low OPA/stationary + low LTPA vs.
Low OPA/stationary + high LTPA
HR = 0.63, 95% CI: 0.47, 0.85

Ref = Low OPA/movement + low LTPA vs.
Low OPA/movement + high LTPA
HR = 0.92, 95% CI: 0.55, 1.55

Ref = High OPA + low LTPA vs.
high OPA + high LTPA
HR = 1.07, 95% CI: 0.73, 1.56</td><td><u>Analysis #1:</u>
Ref = low OPA/stationary + low LTPA vs.
Low OPA/stationary + high LTPA
HR = 0.73, 95% CI: 0.50, 7.09
Low OPA/movement + low LTPA
HR = 1.10, 95% CI: 0.69, 1.77
Low OPA/movement + high LTPA
HR = 1.04, 95% CI: 0.60, 1.79
High OPA + low LTPA
HR = 0.82, 95% CI: 0.55, 1.22
High OPA + high LTPA
HR = 0.78, 95% CI: 0.52, 1.15

<u>Analysis #2:</u>
Ref = Low OPA/stationary + low LTPA vs.
Low OPA/stationary + high LTPA
HR = 0.73, 95% CI: 0.49, 1.09

Ref = Low OPA/movement + low LTPA vs.
Low OPA/movement + high LTPA
HR = 0.93, 95% CI: 0.50, 1.72

Ref = High OPA + low LTPA vs.
high OPA + high LTPA
HR = 1.05, 95% CI: 0.65, 1.70</td><td><u>Analysis #1:</u>
Ref = low OPA/stationary + low LTPA vs.
Low OPA/stationary + high LTPA
HR = 0.62, 95% CI: 0.40, 0.96
Low OPA/movement + low LTPA
HR = 0.73, 95% CI: 0.42, 1.25
Low OPA/movement + high LTPA
HR = 0.93, 95% CI: 0.45, 1.92
High OPA + low LTPA
HR = 1.10, 95% CI: 0.63, 1.94
High OPA + high LTPA
HR = 1.21, 95% CI: 0.55, 2.63

<u>Analysis #2:</u>
Ref = Low OPA/stationary + low LTPA vs.
Low OPA/stationary + high LTPA
HR = 0.67, 95% CI: 0.43, 1.03

Ref = Low OPA/movement + low LTPA vs.
Low OPA/movement + high LTPA
HR = 1.31, 95% CI: 0.49, 3.52

Ref = High OPA + low LTPA vs.
high OPA + high LTPA
HR = 1.00, 95% CI: 0.34, 2.96</td><td>Sig. interaction between OPA and LTPA.

High LTPA had a more pronounced and statistically significant association with reduced diabetes risk for those involved in low OPA and stationary jobs, with a weaker and non-statistically significant association for those with low OPA involving movement.

In contrast, high LTPA was associated with a non-significant increase in diabetes risk for those in high OPA jobs.

An interaction analysis showed similar findings where the relationship between high LTPA and diabetes risk in the overall sample was attenuated by exposure to low OPA and stationary work compared to high OPA.

No evidence of interaction effects among men or women.</td></tr>
<tr><td colspan="7">Metabolic syndrome</td></tr>
<tr><td>Kuwahara, 2016</td><td>1. Sitters (sedentary)
2. Heavy labour (active)</td><td>Model:

Covariates: age, sex, smoking, BMI, alcohol, shift work, sleep duration</td><td><u>Among sedentary workers:</u>
Ref = no LTPA vs.
Very low LTPA
HR = 1.01, 95% CI: 0.89, 1.16
Low LTPA
HR = 0.91, 95% CI: 0.80, 1.03
Medium LTPA
HR = 0.86, 95% CI: 0.77, 0.95
High LTPA
HR = 0.83, 95% CI: 0.71, 0.98
Very high LTPA
HR = 0.88, 95% CI: 0.74, 1.05

<u>Among active workers:</u>
Ref = no LTPA vs.
Very low LTPA</td><td>NR</td><td>NR</td><td>NS interaction between OPA and LTPA.

Higher LTPA led to reduced risk of metabolic syndrome. This effect was statistically significant in the sedentary workers, and was lower and non-significant in the active workers.</td></tr>
</table>

First author, year	OPA groups	Model, covariates	Effect estimates			Narrative synthesis of findings
			Combined men and women	Men	Women	
			HR = 1.00, 95% CI: 0.83, 1.21<br>Low LTPA<br>HR = 1.01, 95% CI: 0.84, 1.20<br>Medium LTPA<br>HR = 1.04. 95% CI: 0.90, 1.20<br>High LTPA<br>HR = 0.97, 95% CI: 0.77, 1.23<br>Very high LTPA<br>HR = 0.88, 95% CI: 0.69, 1.12<br><br>P-values for trend effect: sedentary (p = 0.003), active (p = 0.44), and interaction OPA*LTPA (p = 0.38).			
**Musculoskeletal pain**						
Haukka, 2012<br><br>*MSK pain	Intermittent movers	**Model:** logistic regression<br><br>**Covariates:** age, smoking, BMI, baseline level of MSKP, psychosocial factors at work, organizational reforms	N/A	N/A	Moderate vs. high LTPA:<br>OR = 1.7, 95% CI: 0.8, 3.8<br>Low LTPA vs. high LTPA:<br>OR = 2.0, 95% CI: 0.9, 4.4<br><br>Logistic regressions assessing if LTPA predicted the course of MSKP trajectory over the 2-year follow-up.<br><br>Risk of high trajectory vs. low MSKP trajectory:<br>Moderate vs. high LTPA:<br>**OR = 2.4, 95% CI: 1.2, 4.9**<br>Low vs. high LTPA:<br>**OR = 2.3, 95% CI: 1.1, 4.7**<br><br>Risk of ascending trajectory vs. low MSKP trajectory:<br>Moderate vs. high LTPA:<br>OR = 1.2, 95% CI: 0.5, 3.4<br>Low vs. high LTPA:<br>OR = 1.5, 95% CI: 0.5, 4.3<br><br>Risk of descending trajectory vs. high MSKP trajectory (change of ref. group):<br>High vs. low LTPA:<br>OR = 0.8, 95% CI: 0.3, 2.2<br>Moderate vs. low LTPA:<br>OR = 1.1, 95% CI: 0.5, 2.3	Workers with low or moderate LTPA had higher odds of belonging to the high trajectory of MSKP compared with high LTPA.<br><br>LTPA did not predict occurrence of MSKP at 2-year follow-up.
Holtermann, 2009<br><br>*MSK pain	Heavy labour (cleaners)	**Model:** non-parametric Wilcoxon signed-rank test<br><br>**Covariates:** none	N/A	N/A	Compared cases (with MSKP) vs. controls for difference in LTPA<br><br>Proportion reporting LTPA level 3 or 4 among cases = 34.9% vs. controls = 40%, p-value = 0.94	No difference in LTPA between cleaners with and without musculoskeletal symptoms.

First author, year	OPA groups	Model, covariates	Effect estimates			Narrative synthesis of findings
			Combined men and women	Men	Women	
Jakobsen, 2015<br><br>*MSK pain	Heavy labour (healthcare workers)	**Model:** mean differences<br><br>**Covariates:** age, pain intensity at baseline	N/A	N/A	Baseline vs. follow-up:<br>Work: **MD = -0.8, 95% CI: -1.1, -0.5**<br>Home: MD = -0.2, 95% CI: -0.6, 0.1<br><br>Exercise at home group vs. exercise at work: **MD = -0.7, 95% CI: -1.0, -0.3**	Exercises sessions at work resulted in a decrease of MSKP among healthcare workers.<br><br>Compared with the home exercise group, average pain intensity decreased in the at work exercise group, however, exercise adherence was ½ in the home vs. work group (21% vs. 45%, respectively).
Korhonen, 2003<br><br>*Neck pain	Sitters (office workers)	**Model:** logistic regression<br><br>**Covariates:** age, sex, smoking, VDU working time, physical work environment, ergonomic exposures	Exercise ≤ 1 times/week vs. exercise ≥ 2 times/week: OR = 1.4, 95% CI: 0.7, 2.4	NR	NR	No effect of frequency of physical exercise on developing neck pain over 12 months.
Miranda, 2001<br><br>*Shoulder pain	Heavy labour (Employees of a forestry company)	Model:<br><br>**Covariates:** age, sex	No quantitative results reported.	NR	NR	No statistical significant effect of LTPA detected.
Pedersen, 2013<br><br>*Back, neck and upper extremity pain	Sitters (industrial laboratory technicians)	**Model:** mean differences<br><br>**Covariates:** pain intensity at baseline	Intention-to-treat analysis across the 1-year intervention reveals significant group by time effect for pain in neck, right shoulder, right hand, lower back and DASH.<br><br>No significant changes were observed during the intervention for left-side shoulder, elbow and hand (very low at baseline – data not shown).	NR	NR	Significant intervention effects observed for pain in neck, right shoulder, right hand, lower back and DASH scores. No significant intervention effects for left shoulder, elbow or hand, but these levels were very low at baseline.<br><br>Largest effects in terms of decrease in MSKP and disability was attained during the time of the supervised intervention, however, it was seen that after participation in 20-week strength training intervention there was a long-term effect, i.e. the decrease of MSKP/disability maintained at 1-year follow-up
Riihimaki, 1994<br><br>*Sciatic pain	1. Sitters<br>2. Standers<br>3. Heavy labour	**Model:** logistic regression	N/A	Recreation/sport max 1x/week vs. >1x/week<br><br>Machine operators:<br>RR = 1.24, 95% CI: 0.86, 1.81	N/A	Exercise was associated with an increased (although not statistically significant) risk of back pain. The effect was

First author, year	OPA groups	Model, covariates	Effect estimates			Narrative synthesis of findings
			Combined men and women	Men	Women	
		**Covariates:** smoking, history of back pain		Carpenters<br>RR = 1.37, 95% CI: 0.94, 2.00<br><br>Office workers<br>RR = 1.09, 95% CI: 0.67, 1.79		attenuated among office workers.
Sihawong, 2014<br><br>*neck pain	Sitters (office workers)	**Models:** Cox proportional hazards<br><br>**Covariates:** age, sex, psychological scores	Effects of exercise program on incident neck pain:<br>**HR = 0.45, 95% CI: 0.28, 0.71; p = 0.001**	NR	NR	The intervention is effective for the prevention of neck pain in office works.
Sihawong, 2014b<br><br>*LBP	Sitters (office workers)	**Models:** Mean differences, Cox proportional hazards<br><br>**Covariates:** age, sex, job control, psychological job demand, physical job demand, previous history of working as an office worker, # of working h/day, frequency of reaching, lifting moderate to heavy objects, neck extension and flexion, using a computer, sitting for >2 h/day, standing for >2 h/day, self-rated ergonomics	Pain intensity measured by VAS:<br>Intervention: M = 4.7 (1.5)<br>Control: M = 4.6 (1.4), p = 0.762<br><br>Effects of exercise program on incident LBP<br>**HR = 0.37, 95% CI: 0.22, 0.64, p<0.001**	NR	NR	Results show no significant difference in pain intensity between intervention and control groups.<br><br>The intervention is effective for the prevention of LBP in office works.
Van den Heuvel, 2005<br><br>*Neck and upper limb pain	Sedentary work (sitters) vs. more active work	**Model:** generalized estimating equation (GEE) method<br><br>**Covariates:** age, sex, smoking, alcohol	**<u>Neck/shoulder symptoms</u>**<br>**Sedentary work:**<br>Ref = Practiced sports 0-3 months vs.<br>Practice sports 4-9 months<br>OR = 0.83, 95% CI: 0.58, 1.20<br>**Practiced sports ≥10 months**<br>**OR = 0.70, 95% CI: 0.53, 0.94**<br>Practiced sports <1 h/week<br>OR = 1.00 (ref)<br>Practiced sports 1-3 h/week<br>OR = 0.77, 95% CI: 0.56, 1.04<br>Practiced sports ≥3 h/week<br>OR = 0.83, 95% CI: 0.60, 1.16	NR	NR	Aside from sporting activity for at least 10 months, there is no noticeable difference between sedentary and more active workers.<br><br>LTPA (sports/week) is associated with a reduced risk of neck/shoulder symptoms among sedentary workers. This effect is attenuated among active workers.

First author, year	OPA groups	Model, covariates	Effect estimates			Narrative synthesis of findings
			Combined men and women	Men	Women	
			No walking/cycling<br>OR = 1.00 (ref)<br>10-150 min/week<br>OR = 1.20, 95% CI: 0.91, 1.59<br>≥150 min/week<br>OR = 1.02, 95% CI: 0.67, 1.56<br><br>**More active work:**<br>Ref = Practiced sports 0-3 months vs.<br>Practice sports 4-9 months<br>OR = 1.11, 95% CI: 0.82, 1.52<br>Practiced sports ≥10 months<br>OR = 0.93, 95% CI: 0.71, 1.21<br>Practiced sports <1 h/week<br>OR = 1.00 (ref)<br>Practiced sports 1-3 h/week<br>OR = 1.07, 95% CI: 0.80, 1.44<br>Practiced sports ≥3 h/week<br>OR = 1.01, 95% CI: 0.76, 1.34<br>No walking/cycling<br>OR = 1.00 (ref)<br>10-150 min/week<br>OR = 1.07, 95% CI: 0.85, 1.34<br>≥150 min/week<br>OR = 0.85, 95% CI: 0.56, 1.28<br><br>**<u>Elbow/wrist/ hand symptoms</u>**<br>**Sedentary work:**<br>Ref = Practiced sports 0-3 months vs.<br>Practice sports 4-9 months<br>OR = 1.19, 95% CI: 0.77, 1.83<br>Practiced sports ≥10 months<br>OR = 1.05, 95% CI: 0.72, 1.54<br>Practiced sports <1 h/week<br>OR = 1.00 (ref)<br>Practiced sports 1-3 h/week<br>OR = 1.00, 95% CI: 0.65, 1.52<br>Practiced sports ≥3 h/week<br>OR =0.99, 95% CI: 0.64, 1.53<br>No walking/cycling<br>OR = 1.00 (ref)<br>10-150 min/week<br>OR = 1.01, 95% CI: 0.71, 1.42<br>≥150 min/week<br>OR = 0.93, 95% CI: 0.52, 1.67<br><br>**More active work:**<br>Ref = Practiced sports 0-3 months vs.<br>Practice sports 4-9 months<br>OR = 1.10, 95% CI: 0.78, 1.58<br>Practiced sports ≥10 months			Based on significance: Sporting activities ≥10 months/year are associated with reduced neck/shoulder symptoms among employees with sedentary work, but not active work.<br><br>No other significant protective effects were observed for other LTPA/worker grouping, though there was trending toward protective effects with greater frequency of sporting and active transportation.

First author, year	OPA groups	Model, covariates	Effect estimates			Narrative synthesis of findings
			**Combined men and women**	**Men**	**Women**	
			OR = 0.89, 95% CI: 0.66, 1.20<br>Practiced sports <1 h/week<br>OR = 1.00 (ref)<br>Practiced sports 1-3 h/week<br>OR = 1.08, 95% CI: 0.77, 1.52<br>Practiced sports ≥3 h/week<br>OR = 0.80, 95% CI: 0.56, 1.13<br>No walking/cycling<br>OR = 1.00 (ref)<br>10-150 min/week<br>OR = 1.10, 95% CI: 0.83, 1.46<br>≥150 min/week<br>OR = 0.92, 95% CI: 0.58, 1.45<br><br>*Also includes results for sickness absence due to neck and upper limb symptoms			
Yip, 2004<br><br>*LBP	Heavy labour (nurses)	**Model:** Chi-square test<br><br>**Covariates:** none	**<u>Nurses with LBP</u>**<br>Active LTPA = 12.5%<br>Underactive LTPA = 51.8%<br>Sedentary = 35.7%<br><br>**<u>Nurses without LBP</u>**<br>Active LTPA = 11.4%<br>Underactive LTPA = 40.9%<br>Sedentary = 47.7%<br><br>Chi-square p = 0.35	NR	NR	Among nurses, incidence of low back pain was similar regardless of activity level.
**Depression**						
Bernaards, 2006	Sitters vs. non-sitters	**Model:** generalized estimating equation (GEE) method<br><br>**Covariates:** sex, psychological complaints 1-year earlier, time of measurement	**<u>Sedentary job:</u>**<br>**Simple GEE model (controlling for previous depression):**<br>PA <1 per month vs.<br>1-3 x per month and sedentary job<br>OR = 0.73, 95% CI: 0.53, 1.00, p = 0.05<br>1-2 x per week and sedentary job<br>**OR = 0.64, 95% CI: 0.46, 0.88, p = 0.01**<br>≥3 x per week and sedentary job<br>**OR = 0.57, 95% CI: 0.35, 0.94, p = 0.03**<br><br>**GEE with time lag (future depression):**<br>PA <1 per month vs.<br>1-3 x per month and sedentary job<br>OR = 0.83, 95% CI: 0.58, 1.20, p = 0.33<br>1-2 x per week and sedentary job<br>**OR = 0.62, 95% CI: 0.43, 0.91, p = 0.01**<br>≥3 x per week and sedentary job<br>OR = 0.83, 95% CI: 0.50, 1.37, p = 0.46<br><br>**<u>Non-sedentary job:</u>**<br>**Simple GEE model (controlling for previous depression):**	NR	NR	A dose-response relationship between the frequency of strenuous PA and depression was observed.<br><br>The higher the frequency of strenuous PA, the lower the risk of depression.<br><br>Workers with a sedentary job who engaged in strenuous PA at least 1-2 x per week were at a significantly lower risk of depression than those who engaged in strenuous PA < 1 x month.<br><br>No significant association between strenuous PA and a lower risk of depression in workers with a non-sedentary job.

First author, year	OPA groups	Model, covariates	Effect estimates			Narrative synthesis of findings
			Combined men and women	Men	Women	
			PA <1 per month vs.<br>1-3 x per month and sedentary job<br>OR = 0.99, 95% CI: 0.77, 1.27, p = 0.92<br>1-2 x per week and sedentary job<br>OR = 0.96, 95% CI: 0.74, 1.24, p = 0.76<br>≥3 x per week and sedentary job<br>OR = 0.76, 95% CI: 0.55, 1.06, p = 0.11<br><br>**GEE with time lag (future depression):**<br>PA <1 per month vs.<br>1-3 x per month and sedentary job<br>OR = 0.91, 95% CI: 0.64, 1.29, p = 0.59<br>1-2 x per week and sedentary job<br>OR = 0.84, 95% CI: 0.62, 1.14, p = 0.27<br>≥3 x per week and sedentary job<br>OR = 0.80, 95% CI: 0.53, 1.21, p = 0.29			Workers with a sedentary job who engaged in strenuous PA once to twice a week, but not at a higher frequency, were at a reduced risk of future depression.<br><br>In contrast, the relation between strenuous PA (1–2 times per week) and a reduced risk of future depression was not found in workers with a non-sedentary job.
**CVD & IHD Incidence**						
Allesoe, 2015<br><br>*IHD	1. Sitters<br>2. Standers<br>3. Intermittent movers<br>4. Heavy labour	**Model:** Cox proportional hazards<br><br>**Covariates:** age, smoking, BMI, alcohol, family history of IHD, diabetes, work pressure, job influence, shift work and work hours/week	N/A	N/A	Moderate PA at work and vigorous LTPA (ref) vs.:<br><br>Sedentary at work and moderate LTPA<br>HR = 1.46, 95% CI: 0.89, 2.37<br>Sedentary at work and vigorous LTPA<br>**HR = 2.17, 95% CI: 1.25, 3.79**<br>Moderate PA at work and moderate LTPA<br>**HR = 1.71, 95% CI: 1.10, 2.66**<br>High PA at work and moderate LTPA<br>**HR = 2.09, 95% CI: 1.36, 3.21**<br>High PA at work and vigorous LTPA<br>**HR = 1.75, 95% CI: 1.10, 2.80**<br><br>Interaction between PA at work and during leisure time found to be significant p = 0.045.	High PA at work was associated with increased risk of IHD in all three combinations with LTPA, but lowest risk among those with vigorous PA during leisure time, but the confidence intervals overlap.<br><br>Sedentary work was associated with an increased risk of IHD among nurses with vigorous LTPA.
Clays, 2013<br><br>*CHD	Low vs. high OPA	**Model:** Cox proportional hazards<br><br>**Covariates:** age, education, smoking, BP, BMI, occupational class, job strain, alcohol, diabetes, total cholesterol, HDL	N/A	**<u>Analysis #1:</u>**<br>Low OPA/moderate-high LTPA (ref) vs.<br>Low OPA/low LTPA<br>HR = 1.98, 95% CI: 0.99, 3.96<br>High OPA/low LTPA<br>HR = 1.51, 95% CI: 0.54, 4.19<br>High OPA/mod-high LTPA<br>**HR = 3.82, 95% CI: 1.41, 10.36**<br><br>OPA*LTPA interaction term:<br>**HR = 5.01, 95% CI: 1.43, 17.53**<br><br>**<u>Analysis #2 stratified by OPA group:</u>**<br>Within low OPA group:<br>Low LTPA (ref) vs. moderate-to-high LTPA<br>HR = 0.52, 95% CI: 0.26, 1.04	N/A	A significant multiplicative interaction was found between LTPA and OPA in relation to incidence of coronary.<br><br>Men who combined high physical work demands with moderate to high levels of LTPA showed an almost four times significantly higher incidence rate of coronary events compared to the lowest risk group with low OPA and moderate to high LTPA.<br><br>The combined analysis showed that low LTPA was related with

First author, year	OPA groups	Model, covariates	Effect estimates			Narrative synthesis of findings
			Combined men and women	Men	Women	
				Within high OPA group (ref) vs. moderate-to-high LTPA<br>HR = 2.30, 95% CI: 0.75, 7.09		an increased coronary event incidence, although the fully adjusted association was no longer significant when combined with high OPA while it became borderline significant in combination with low physical job demands.<br><br>In men with low OPA, there appears to be an independent protective effect of moderate to high LTPA with CHD. No beneficial association of LTPA was observed in workers exposed to high OPA.
Ferrario, 2018<br><br>*CHD events	Low, medium, high OPA	**Model:** Cox proportional hazards<br><br>**Covariates:** age, cohort type, education, BMI, total cholesterol, HDL, SBP, smoking, diabetes, alcohol	N/A	**CHD events:**<br>Low OPA + intermed./rec SpPA vs. Low OPA + poor SpPA (ref)<br>**HR = 0.45, 95% CI: 0.24, 0.87**<br><br>Intermed. OPA + intermed./rec. SpPA vs. intermed. OPA + poor SpPA (ref)<br>HR = 1.20, 95% CI: 0.54, 2.67<br><br>High OPA + intermed./rec. SpPA vs. high OPA + poor SpPA (ref)<br>HR = 1.84, 95% CI: 0.88, 3.87<br><br>**CVD events:**<br>Low OPA + Intermed./rec. SpPA vs. Low OPA + poor SpPA (ref)<br>**HR 0.45, 95% CI: 0.25, 0.82**<br><br>Intermed. OPA + intermed./rec. SpPA vs. intermed. OPA + poor SpPA (ref)<br>HR 0.93, 95% CI: 0.43, 1.98<br><br>High OPA + intermed./rec. SpPA vs. high OPA + poor SpPA (ref)<br>HR = 1.66, 95% CI: 0.87, 3.14	N/A	Risk reduction was found among sedentary workers, while increased risk was found among workers with higher OPA levels.
Fransson, 2004<br><br>*MI	1. Sitters<br>2. Heavy labour	**Model:** Logistic regression<br><br>**Covariates:** age, smoking, fiber intake, hospital catchment area, socioeconomic status (blue or white collar based	NR	Results shown in a figure.	Results shown in a figure.	Beneficial effect of regular LTPA among each category of perceived OPA, but more statistically significant among those without repetitive lifting at work or heavy lifting at work.<br><br>Lifting or carrying heavy things at work in combination with a lack of regular LTPA resulted in

First author, year	OPA groups	Model, covariates	Effect estimates			Narrative synthesis of findings
			Combined men and women	Men	Women	
		on occupation and education level), alcohol				increased MI risk among women.<br><br>Not having demanding household work in combination with sitting a lot at work was hazardous for women.<br><br>Simultaneous lack of LTPA and having a job sitting most of the work day was particularly harmful esp. among women.
Holtermann, 2012b<br><br>*MI	1. Sitters<br>2. Intermittent movers<br>3. Heavy labour	**Model:** Cox proportional hazards<br><br>**Covariates:** Age, income, smoking, BP, BMI, alcohol, diabetes, BP medication, cholesterol	N/A	Low LTPA and low OPA (ref) vs.<br>Low LTPA and moderate OPA<br>HR = 1.39, 95% CI: 0.76, 2.53<br>Low LTPA and high OPA<br>HR = 1.15, 95% CI: 0.62, 2.13<br><br>Moderate LTPA and low OPA (ref) vs.<br>Moderate LTPA and moderate OPA<br>HR = 1.27, 95% CI: 0.89, 1.80<br>Moderate LTPA and high OPA<br>HR = 1.41, 95% CI: 0.96, 2.06<br><br>High LTPA and low OPA (ref) vs.<br>High LTPA and moderate OPA<br>HR = 1.27, 95% CI: 0.87, 1.86<br>High LTPA and high OPA<br>HR = 1.04, 95% CI: 0.69, 1.55	Low LTPA and low OPA (ref) vs.<br>Low LTPA and moderate OPA<br>HR = 1.03, 95% CI: 0.49, 2.15<br>Low LTPA and high OPA<br>HR = 1.55, 95% CI: 0.55, 4.35<br><br>Moderate LTPA and low OPA (ref) vs.<br>Moderate LTPA and moderate OPA<br>HR = 0.65, 95% CI: 0.45, 0.95<br>Moderate LTPA and high OPA<br>HR = 0.78, 95% CI: 0.46, 1.33<br><br>High LTPA and low OPA (ref) vs.<br>High LTPA and moderate OPA<br>HR = 1.00, 95% CI: 0.49, 2.01<br>High LTPA and high OPA<br>HR = 1.18, 95% CI: 0.54, 2.60	Among men, with low or moderate LTPA, risk of MI increased with higher OPA, though not significantly, and no significant interaction was found.<br><br>Among women, OPA did not increase the risk of MI in any of the LTPA and no significant interaction was found.
Hu, 2005<br><br>*Stroke	1. Sitters (light OPA)<br>2. Intermittent movers (moderate OPA)<br>3. Heavy labourers (active OPA)	**Model:** Cox proportional hazards<br><br>**Covariates:** age, sex, education, smoking, BP, BMI, area, study year, cholesterol, alcohol, diabetes	Ref = Light OPA, low LTPA, and no commuting vs.<br><br>Light OPA, low LTPA, and walking or cycling to/from work >1 min/day<br>**HR = 0.79, 95% CI: 0.63, 0.98***<br>Light OPA, moderate or high LTPA, no active commuting<br>**HR = 0.81, 95% CI: 0.71, 0.93***<br>Light OPA, moderate or high LTPA, and walking or cycling to/from work >1 min/day<br>**HR = 0.69, 95% CI: 0.57, 0.82***<br>Moderate or active OPA, low LTPA, no active commuting<br>HR = 0.86, 95% CI: 0.74, 1.00<br>Moderate or active OPA, low LTPA, walking or cycling to/from work >1 min/day<br>**HR = 0.79, 95% CI: 0.69, 0.90***<br>Moderate or active OPA, moderate or high LTPA, no active commuting<br>**HR = 0.69, 95% CI: 0.60, 0.81***	NR	NR	Compared with those who report low levels of LTPA, OPA, and commuting PA, those who reported 2 or 3 types of moderate to high PA had a 21% to 31% decreased risk of stroke, whereas those who reported only 1 of 3 types of moderate to high PA had a 14% to 21% decreased risk of stroke.

First author, year	OPA groups	Model, covariates	Effect estimates			Narrative synthesis of findings
			Combined men and women	Men	Women	
			Moderate or active OPA, moderate or high LTPA, walking or cycling to/from work >1 min/day<br>**HR = 0.69, 95% CI: 0.61, 0.79***			
Hu, 2007<br><br>*CHD	1. Sitters (low OPA)<br>2. Intermittent movers (mod. OPA)<br>3. Heavy labour (high OPA)	**Model:** Cox proportional hazards<br><br>**Covariates:** age, education, smoking, SBP, BMI, study year, cholesterol, alcohol, diabetes	NR	Ref = Low LTPA involving no active commuting (no walking or cycling to and from work daily) and light OPA<br><br>Low LTPA involving active commuting (walking or cycling to and from work ≥1 min/day) and light OPA vs. ref<br>HR = 0.90, 95% CI: 0.71, 1.15<br><br>Mod. or high LTPA involving no active commuting (no walking or cycling to and from work daily) and light OPA vs. ref<br>**HR = 0.77, 95% CI: 0.67, 0.89**<br><br>Mod. or high LTPA involving active commuting (walking or cycling to and from work ≥1 min/day) and light OPA vs. ref<br>**HR = 0.80, 95% CI: 0.68, 0.96**<br><br>Low LTPA involving no active commuting (no walking or cycling to and from work daily) and mod. or high OPA vs. ref<br>**HR = 0.74, 95% CI: 0.63, 0.87**<br><br>Low LTPA involving active commuting (walking or cycling to and from work ≥1 min/day) and mod. or high OPA vs. ref<br>**HR = 0.75, 95% CI: 0.65, 0.87**<br><br>Mod. or high LTPA involving no active commuting (no walking/cycling to and from work daily) and mod. or high OPA vs. ref<br>**HR = 0.77, 95% CI: 0.67, 0.89**<br><br>Mod. or high LTPA involving active commuting (walking/cycling to/from work ≥1 min/day) and mod. or high OPA vs. ref<br>**HR = 0.75, 95% CI: 0.66, 0.86**	Ref = Low LTPA involving no active commuting (no walking or cycling to and from work daily) and light OPA<br><br>Low LTPA involving active commuting (walking or cycling to and from work ≥1 min/day) and light OPA vs. ref<br>HR = 0.79, 95% CI: 0.61, 1.00<br><br>Mod. or high LTPA involving no active commuting (no walking or cycling to and from work daily) and light OPA vs. ref<br>**HR = 0.77, 95% CI: 0.66, 0.91**<br><br>Mod. or high LTPA involving active commuting (walking or cycling to and from work ≥1 min/day) and light OPA vs. ref<br>**HR = 0.56, 95% CI: 0.43, 0.72**<br><br>Low LTPA involving no active commuting (no walking or cycling to and from work daily) and mod. or high OPA vs. ref<br>**HR = 0.70, 95% CI: 0.58, 0.84**<br><br>Low LTPA involving active commuting (walking or cycling to and from work ≥1 min/day) and mod. or high OPA vs. ref<br>**HR = 0.61, 95% CI: 0.51, 0.72**<br><br>Mod. or high LTPA involving no active commuting (no walking/cycling to and from work daily) and mod. or high OPA vs. ref<br>**HR = 0.55, 95% CI: 0.44, 0.67**<br><br>Mod. or high LTPA involving active commuting (walking/cycling to/from work ≥1 min/day) and mod. or high OPA vs. ref<br>**HR = 0.61, 95% CI: 0.52, 0.72**	Among men, moderate or high levels of LTPA were strongly associated with a reduced risk of CHD among men with low OPA, but the protective effect of LTPA was not seen among those with a more physically active work.<br><br>Among women, LTPA was associated with a reduced risk of CHD at all levels of OPA.<br><br>In addition, commuting PA had a moderate inverse association with CHD risk.<br><br>Men with high levels of all three types of PA had a lower age-adjusted risk of CHD as compared with the least active men.<br><br>In women, the risk reduction was even larger.<br><br>Exclusion of the participants who died during the first 2 years of follow-up did not appreciably change the results (data not shown).
Petersen, 2012<br><br>*IHD	1. Heavy labour (heavy lifting)<br>2. Sedentary + intermittent (no heavy lifting)	**Model:** Cox proportional hazards<br><br>**Covariates:** age, education, smoking, alcohol, stress, OPA	NR	Ref = High LTPA, no occupational lifting<br><br>High LTPA, occupational lifting vs ref.<br>HR = 1.38, 95% CI: 0.82, 2.35<br>Low LTPA, no occupational lifting vs ref.<br>HR = 1.31, 95% CI: 0.95, 1.81<br>Low LTPA, occupational lifting vs ref.<br>**HR = 2.05**, 95% CI: **1.39, 3.03**	Ref = High LTPA, no occupational lifting<br><br>High LTPA, occupational lifting vs ref.<br>HR = 0.24, 95% CI: 0.05, 1.06<br>Low LTPA, no occupational lifting vs ref.<br>HR = 0.79, 95% CI: 0.47, 1.32<br>Low LTPA, occupational lifting vs ref.<br>HR = 0.74, 95% CI: 0.39, 1.41	For men, low LTPA was associated with increased risk of IHD incidence. Effects were stronger among those who did a lot of occupational lifting, compared to those without. Among those with high LTPA, heavy living appeared to raise the risk for IHD compared to

First author, year	OPA groups	Model, covariates	Effect estimates			Narrative synthesis of findings
			Combined men and women	Men	Women	
						those with no occupational lifting.<br><br>Among women the associations were the other way around
Wang, 2010<br><br>*HF	1. Sitters<br>2. Intermittent movers<br>3. Heavy labour<br><br>Low, moderate, high	**Model:** Cox proportional hazards<br><br>**Covariates:** Age, education, smoking, BP, alcohol, study year, history of CVD, diabetes, lung disease or medication use, and cholesterol		Ref = Low LTPA, no commute PA, moderate or active OPA<br><br>Low LTPA, no commute PA, light OPA<br>**HR = 0.78**<br>Low LTPA, >1 min commute PA, light OPA<br>**HR = 0.77**<br>Moderate or high LTPA, no commute PA, light OPA<br>**HR = 0.65**<br>Moderate or high LTPA, >1 min commute PA, light OPA<br>**HR = 0.69**<br>Low LTPA, >1 min commute PA, mod. or active OPA<br>HR = 1.08<br>Moderate or high LTPA, no commute PA, mod. or active OPA<br>**HR = 0.77**<br>Moderate or high LTPA, >1 min commute PA, mod. or active OPA<br>**HR = 0.68**	Ref = Low LTPA, no commute PA, moderate or active OPA<br><br>Low LTPA, no commute PA, light OPA<br>HR = 0.93<br>Low LTPA, >1 min commute PA, light OPA<br>**HR = 0.83**<br>Moderate or high LTPA, no commute PA, light OPA<br>**HR = 0.64**<br>Moderate or high LTPA, >1 min commute PA, light OPA<br>**HR = 0.66**<br>Low LTPA, >1 min commute PA, mod. or active OPA<br>HR = 0.86<br>Moderate or high LTPA, no commute PA, mod. or active OPA<br>HR = 0.92<br>Moderate or high LTPA, >1 min commute PA, mod. or active OPA<br>**HR = 0.72**	High levels of LTPA and commute PA reduce the risk of heart failure. This effect was stronger among males with moderate or active OPA
Wang, 2016<br><br>*MI	1. Sitters<br>2. Intermittent movers<br><br>Low (<33% RAS) vs. high OPA (>33% RAS)<br><br>relative aerobic strain (RAS)	**Model:** Cox proportional hazards<br><br>**Covariates:** age, education, smoking, alcohol, baseline IHD	N/A	Ref = low OPA/high LTPA<br><br>**<u>Men without IHD</u>**<br>High OPA/high LTPA vs. ref<br>HR = 1.10, 95% CI: 0.76, 1.60<br>Low OPA/low LTPA vs ref<br>HR = 1.05, 95% CI: 0.80, 1.37<br>High OPA/low LTPA vs. ref<br>HR = 1.33, 95% CI: 0.99, 1.78<br><br>**<u>Men with IHD</u>**<br>High OPA/ high LTPA vs. ref<br>HR = 1.04, 95% CI: 0.61, 1.79<br>Low OPA/low LTPA vs ref<br>HR = 0.85, 95% CI: 0.50, 1.46<br>High OPA/low LTPA vs. ref<br>HR = 1.36, 95% CI: 0.84, 2.18	N/A	Impact of one PA domain on AMI depended on the level of the other PA domain on the multiplicative but not additive scale, when accounting for individual fitness.<br><br>LTPA did not reveal an independent effect on AMI after accounting for OPA and OPA-LTPA interaction.
Wang, 2019<br><br>*MI or CHD death	1. Sitters (low)<br>2. Heavy labour (very high)	**Model:** Cox proportional hazards<br><br>**Covariates:** age, ethnicity,	N/A	N/A	Ref = High LTPA/low OPA<br><br><u>Cumulative OPA:</u><br>Low OPA + low LTPA<br>HR = 1.24, 95% CI: 1.00, 1.53<br>Mod. OPA + low LTPA	Study shows no overall association between OPA and CHD risk.<br><br>Women with low and mod. OPA + low LTPA have

First author, year	OPA groups	Model, covariates	Effect estimates			Narrative synthesis of findings
			Combined men and women	Men	Women	
	Low, moderate, high and very high	education, income, occupation class, BMI, diabetes, cholesterol, hypertension			**HR = 1.38, 95% CI: 1.12, 1.70**<br>High OPA + low TPA<br>**HR = 1.36, 95% CI: 1.11, 1.67**<br>Very high OPA + low LTPA<br>HR = 1.13, 95% CI: 0.92, 1.40<br>Mod. OPA + high LTPA<br>HR = 1.15, 95% CI: 0.94, 1.47<br>High OPA + high LTPA<br>HR = 1.19, 95% CI: 0.97, 1.47<br>Very high OPA + high LTPA<br>HR = 1.14, 95% CI: 0.92, 1.41<br><br>Recent OPA:<br>Low OPA + low LTPA<br>HR = 1.24, 95% CI: 1.00, 1.53<br>Mod. OPA + low LTPA<br>**HR = 1.38, 95% CI: 1.12, 1.70**<br>High OPA + low TPA<br>**HR = 1.36, 95% CI: 1.11, 1.67**<br>Very high OPA + low LTPA<br>HR = 1.13, 95% CI: 0.92, 1.40)<br>Mod. OPA + high LTPA<br>HR = 1.05, 95% CI: 0.85, 1.30<br>High OPA + high LTPA<br>HR = 1.03, 95% CI: 0.85, 1.24<br>Very high OPA + high LTPA<br>HR = 0.89, 95% CI: 0.70, 1.13<br><br>The associations of CHD with the combined measure of either the cumulative or the most recent OPA and LTPA were significant (**p = .0007** for the interaction between LTPA and cumulative OPA; **p = .005** for the interaction between LTPA and most recent OPA).	increased risk of CHD. Highest increased risk of CHD was among women who performed moderate to high OPA and low LTPA.
**Arrhythmias**						
Skielboe, 2016<br><br>*AF	1. Sitters<br>2. Intermittent movers<br>3. Heavy labour x 2<br><br>Low, moderate, high and very	**Model:** Cox proportional hazards<br><br>**Covariates:** age, sex, education, smoking, BP, BMI, alcohol, resting heart rate, spirometry, cardiac medication,	NR	NR	NR	The overall test for interaction between OPA and LTPA was statistically insignificant

First author, year	OPA groups	Model, covariates	Effect estimates			Narrative synthesis of findings
			Combined men and women	Men	Women	
	high volume OPA	diabetes mellitus, IHD				
**CVD & IHD mortality**						
Barengo, 2004	1. Sitters<br>2. Intermittent movers<br>3. Heavy labour	**Model:** Cox proportional hazards<br><br>**Covariates:** age, sex, education, smoking, SBP, cholesterol, BMI and the other types of PA.	NR	Ref = low OPA/no commuting/low LTPA<br><br>No commute/low LTPA/mod OPA<br>**HR = 0.69**<br>≥ 15min commute/low LTPA/light OPA<br>HR = 0.83<br>≥15min commute/low LTPA/mod OPA<br>HR = 0.86<br>No commute/mod-high LTPA/light OPA<br>HR = 0.90<br>No commute/mod-high LTPA/mod OPA<br>**HR = 0.67**<br>≥15min commute/mod-high LTPA/light OPA<br>HR= 0.85<br>≥15min commute/mod-high LTPA/mod OPA<br>**HR = 0.70**	Ref = low OPA/no commuting/low LTPA<br><br>No commute/low LTPA/mod OPA<br>**HR = 0.58**<br>≥15min commute/low LTPA/light OPA<br>**HR = 0.69**<br>≥15min commute/low LTPA/mod OPA<br>**HR = 0.56**<br>No commute/mod-high LTPA/light OPA<br>**HR = 0.66**<br>No commute/mod-high LTPA/mod OPA<br>**HR = 0.54**<br>≥15min commute/mod-high LTPA/light OPA<br>**HR = 0.49**<br>≥15min commute/mod-high LTPA/mod OPA<br>**HR = 0.55**	There was a statistically significant risk reduction in CVD mortality among women in all joint association forms of PA compared to the references category.<br><br>In men, a significant risk reduction of CVD mortality was mainly found among those who had moderate or high OPA.
Fan, 2019 [36]	Factory (standers) vs. non-factory workers	**Model:** Cox proportional hazards<br><br>**Covariates:** age, sex, education, income, smoking, BP, BMI, marital status, alcohol, intake of red meet, fruits and veg, leisure sedentary time, comorbidities, household air pollution, passive smoking, other domain PA, family history of heart attack, hypertension, diabetes, cooking pollution, heating pollution, passive smoking	Stratified on OPA (low / intermediate / high). In all groups, ref = poor SpPA and group 1 = intermediate/recommended SpPA.<br><br>**CHD events:**<br>Low OPA + Inter/rec SpPA vs. Low OPA + poor SpPA (ref)<br>**HR = 0.45, 95% CI: 0.24, 0.87**<br>Inter. OPA + Inter./rec SpPA vs. inter. OPA + poor SpPA (ref)<br>HR = 1.20, 95% CI: 0.54, 2.67<br>High OPA + Inter/rec SpPA vs. high OPA + poor SpPA (ref)<br>HR = 1.84, 95% CI: 0.88, 3.87<br><br>**CVD events:**<br>Low OPA + Inter/rec SpPA vs. Low OPA + poor SpPA (ref)<br>**HR = 0.45, 95% CI: 0.25, 0.82**<br>Inter. OPA + Inter/rec SpPA vs. inter. OPA + poor SpPA (ref)<br>HR = 0.93, 95% CI: 0.43, 1.98<br>High OPA + Inter/rec SpPA vs. high OPA + poor SpPA (ref)<br>HR = 1.66, 95% CI: 0.87, 3.14	NR	NR	SpPA was strongly dependent of OPA level. Risk reduction found among sedentary workers, while increased risk was found among workers with higher OPA levels.
Fransson, 2004	1. Sitters<br>2. Heavy labour x 2	**Model:** Logistic regression<br><br>**Covariates:** age, smoking, fiber	NR	Narrative findings.	Narrative findings.	Similar effects as those for MI incidence. A beneficial effect of regular LTPA among each category of perceived OPA. Simultaneous lack of LTPA and

First author, year	OPA groups	Model, covariates	Effect estimates			Narrative synthesis of findings
			Combined men and women	Men	Women	
		intake, hospital catchment area, socioeconomic status (blue or white collar based on occupation and education level), alcohol				having a job sitting most of the work day was particularly harmful esp. among women.
Harari, 2015	None-mild OPA vs. moderate-hard OPA (Manufacturing, machine operators)	**Model:** Cox proportional hazards<br><br>**Covariates:** age, education, smoking, BMI, SES, father's country of origin, cholesterol, HDL, hypertension, diabetes, coffee consumption, alcohol, diet, shift work	N/A	Ref = Moderate-hard OPA + no LTPA.<br><br>Non-mild OPA + no LTPA vs. ref<br>HR = 0.78, 95% CI: 0.53, 1.53<br>Moderate-hard OPA + LTPA vs. ref<br>HR = 1.00, 95% CI: 0.58, 1.73<br>Non-mild OPA + LTPA vs. ref<br>HR = 0.44, 95% CI: 0.18, 1.11	N/A	Employees who perform moderate-hard OPA and no LTPA had the greatest risk of all-cause mortality while employees who performed none-light OPA and LTPA had the lowest risk.<br><br>Does not appear to be risk of performing LTPA among moderate-hard OPA group.
Holme, 1981	Sedentary, Moderate, Intermediate, Great (not described)	**Model:** Death rates per 1,000<br><br>**Covariates:** None	N/A	Death rates presented as number of deaths divided by number of men at risk per 1000.<br><br>Group 1 - Sedentary at work + sedentary at leisure time, CHD death rate per 1000: 5.7<br><br>Group 2 - Moderate at work + sedentary at leisure time, CHD death rate per 1000: 9.4<br><br>Group 3 - Intermediate at work + sedentary at leisure time, CHD death rate per 1000: 7.5<br><br>Group 4 - Great at work + sedentary at leisure time, CHD death rate per 1000: 13.5<br><br>Group 5 - Sedentary at work + moderate at leisure time, CHD death rate per 1000: 2.8<br><br>Group 6 - Moderate at work + moderate at leisure time, CHD death rate per 1000:<br><br>Group 7 - Intermediate at work + moderate at leisure time, CHD death rate per 1000: 6.2<br><br>Group 8 - Great at work + moderate at leisure time, CHD death rate per 1000: 7.2	N/A	Increasing LTPA across OPA groups associated with falling CVD and total risk.

First author, year	OPA groups	Model, covariates	Effect estimates			Narrative synthesis of findings
			Combined men and women	Men	Women	
				Group 9 - Sedentary at work + intermediate at leisure time, CHD death rate per 1000: 2.8<br><br>Group 10 - Moderate at work + intermediate at leisure time, CHD death rate per 1000: 2.7<br><br>Group 11 - Intermediate at work + intermediate at leisure time, CHD death rate per 1000: 5.0<br><br>Group 12 - Great at work + intermediate at leisure time, CHD death rate per 1000: -<br><br>Group 13 - Sedentary at work + great at leisure time, CHD death rate per 1000:<br><br>Group 14 - Moderate at work + great at leisure time, CHD death rate per 1000:<br><br>Group 15 - Intermediate at work + great at leisure time Total death rate per 1000: , CHD death rate per 1000: -<br><br>Group 16 - Great at work + great at leisure time, CHD death rate per 1000: -		
Holtermann, 2009<br><br>*IHD	1. Sitters<br>2. Intermittent movers<br>3. Heavy labour	**Model:** Cox proportional hazards<br><br>**Covariates:** age, smoking, BP, BMI, treatment of diabetes or hypertension, alcohol, social class	N/A	Ref = Low LTPA<br><br>**<u>Low OPA</u>**<br>Moderate LTPA vs. ref<br>HR = 0.72, 95% CI: 0.47, 1.10<br>High LTPA vs. ref<br>HR = 0.61, 95% CI: 0.30, 1.26<br><br>**<u>Moderate OPA</u>**<br>Moderate LTPA vs. ref<br>HR = 0.79, 95% CI: 0.59, 1.05<br>High LTPA vs. ref<br>**HR = 0.37, 95% CI: 0.19, 0.70**<br><br>**<u>High OPA</u>**<br>Moderate LTPA vs. ref<br>**HR = 0.62, 95% CI: 0.40, 0.97**<br>High LTPA vs. ref<br>HR = 0.82, 95% CI: 0.42, 1.56	N/A	Men who were moderately or highly physically active at leisure time, had a decreased risk of IHD mortality in all groups, though not always statistically significant
Holtermann, 2012a<br><br>*IHD	1. Sitters<br>2. Intermittent movers<br>**3.** Heavy labour x 2	**Model:** Cox proportional hazards<br><br>**Covariates:** age, smoking, BMI, alcohol, BP	N/A	Ref = Low LTPA<br><br>**<u>Low physical work demands only:</u>**<br>Medium LTPA vs. ref<br>HR = 0.74, 95% CI: 0.47, 1.15<br>High LTPA vs. ref<br>HR = 0.76, 95% CI: 0.38, 1.53	N/A	Low LTPA was a statistically significant risk factor among those with a moderate or high level of physical work demands. Among those with low physical work demands, the association

First author, year	OPA groups	Model, covariates	Effect estimates			Narrative synthesis of findings
			Combined men and women	Men	Women	
		including treatment, physical fitness, # of hours at work, psychosocial stress at work and leisure, social class		<u>**Moderate physical work demands only:**</u><br>Medium LTPA vs. ref<br>HR = 0.78, 95% CI: 0.59, 1.04<br>High LTPA vs. ref<br>**HR = 0.37, 95% CI: 0.19, 0.72**<br><br><u>**High physical work demands only:**</u><br>Medium LTPA vs. ref<br>**HR = 0.56, 95% CI: 0.36, 0.88**<br>High LTPA vs. ref<br>HR = 0.77, 95% CI: 0.40, 1.48		was weaker and did not reach statistical significance.<br><br>Only among men with high physical work demands, those with highest level of physical fitness had a significantly lower risk of IHD mortality compared with those with a low fitness level.
Holtermann, 2013	Men:<br>1. Sitters<br>2. Intermittent movers<br>3. Heavy labour x 2<br>Women:<br>1. Sitters<br>2. Intermittent movers<br>3. Heavy labour	**Model:** Cox proportional hazards<br><br>**Covariates:** age, income, smoking, BP, BMI, calendar time, alcohol, cholesterol, BP medication		Ref = Low OPA, High LTPA<br><br>Low OPA, Low LTPA vs. ref<br>**HR = 1.54, 95% CI: 1.25, 1.88**<br>Low OPA, Moderate LTPA vs. ref<br>HR = 0.98, 95% CI: 0.83, 1.16<br>Moderate OPA, Low LTPA vs. ref<br>**HR = 1.59, 95% CI: 1.25, 2.01**<br>Moderate OPA, Moderate LTPA vs. ref<br>HR = 0.98, 95% CI: 0.82, 1.17<br>Moderate OPA, High LTPA vs. ref<br>HR = 0.91, 95% CI: 0.75, 1.11<br>High OPA, Low LTPA vs. ref<br>HR = 1.15, 95% CI: 0.87, 1.52<br>High OPA, Moderate LTPA vs. ref<br>HR = 1.07, 95% CI: 0.89, 1.28<br>High OPA, High LTPA vs. ref<br>HR = 1.01, 95% CI: 0.83, 1.23<br>Very high OPA, Low LTPA vs. ref<br>HR = 1.36, 95% CI: 0.95, 1.95<br>Very high OPA, Moderate LTPA vs. ref<br>HR = 1.24, 95% CI: 0.95, 1.63<br>Very high OPA, High LTPA vs. ref<br>HR = 1.01, 95% CI: 0.77, 1.32	Ref = Low OPA, High LTPA<br><br>Low OPA, Low LTPA vs. ref<br>**HR = 1.59, 95% CI: 1.22, 2.07**<br>Low OPA, Moderate LTPA vs. ref<br>HR = 1.20, 95% CI: 0.95, 1.51<br>Moderate OPA, Low LTPA vs. ref<br>**HR = 1.63, 95% CI: 1.27, 2.10**<br>Moderate OPA, Moderate LTPA vs. ref<br>HR = 1.03, 95% CI: 0.83, 1.29<br>Moderate OPA, High LTPA vs. ref<br>HR = 0.89, 95% CI: 0.69, 1.13<br>High OPA, Low LTPA vs. ref<br>**HR = 1.72, 95% CI: 1.24, 2.40**<br>High OPA, Moderate LTPA vs. ref<br>HR = 1.25, 95% CI: 0.96, 1.62<br>High OPA, High LTPA vs. ref<br>HR = 1.03, 95% CI: 0.76, 1.39	There was a consistent risk reductions from higher levels of LTPA in all groups of OPA.<br><br>No statistical interaction between LTPA and OPA in either men or women for cardiovascular mortality (p = 0.14) was found. The effect of LTPA on cardiovascular mortality seems to be independent of the level of OPA.
Salonen, 1988	Active workers vs. sedentary (sitters)	**Model:** incidence rate per 1,000<br><br>**Covariates:** None	NR	<u>**Low BMI, males**</u><br>Active workers, high LTPA = 8.10<br>Active workers, low LTPA = 14.08<br>Sedentary workers, high LTPA = 19.90<br>Sedentary workers, low LTPA = 25.86<br><br><u>**High BMI, males**</u><br>Active workers, high LTPA = 7.37<br>Active workers, low LTPA = 17.14<br>Sedentary workers, high LTPA = 8.64<br>Sedentary workers, low LTPA = 41.67	<u>**Low BMI, females**</u><br>Active workers, high LTPA = 0.45<br>Active workers, low LTPA = 3.18<br>Sedentary workers, high LTPA = 1.14<br>Sedentary workers, low LTPA = 0.00<br><br><u>**High BMI, females**</u><br>Active workers, high LTPA = 2.05<br>Active workers, low LTPA = 0.98<br>Sedentary workers, high LTPA = 6.15<br>Sedentary workers, low LTPA = 0.00	In both sedentary and active workers, those with LTPA had the lowest risk of IHD mortality
Stamatakis, 2013	Sitting vs. non-sitting occupations	**Model:** Cox proportional hazards	NR	No differences in association of main activity at work and mortality by LTPA level.	No significant interaction b/w OPA and LTPA for CVD mortality (p = 0.087).	Among men and women no evidence for an interaction b/w OPA and LTPA for CVD mortality.

First author, year	OPA groups	Model, covariates	Effect estimates			Narrative synthesis of findings
			Combined men and women	Men	Women	
		**Covariates:** age, education, smoking, alcohol, general health, CVD at baseline, cancer at baseline, occupational class, WC, non-OPA, psychological health and social class				Unknown if LTPA infers protection (not reported in paper).
**All-cause mortality**						
Barengo, 2004	1. Sitters<br>2. Intermittent movers<br>3. Heavy labour	**Model:** Cox proportional hazards<br><br>**Covariates:** age, sex, education, smoking, SBP, cholesterol, BMI and the other types of PA.	NR	Ref = no commuting/low LTPA/low OPA<br><br>No commute/low LTPA/mod OPA<br>**HR = 0.67**<br>≥15min commute/low LTPA/ low OPA<br>HR = 0.89<br>≥15min commute/low LTPA/mod OPA<br>**HR = 0.80**<br>No commute/mod-high LTPA/ low OPA<br>**HR = 0.83**<br>No commute/mod-high LTPA/mod OPA<br>**HR = 0.66**<br>≥15min commute/mod-high LTPA/low OPA<br>**HR = 0.83**<br>≥15min commute/mod-high LTPA/mod OPA<br>**HR = 0.67**	Ref = no commuting/low LTPA/low OPA<br><br>No commute/low LTPA/mod OPA<br>**HR = 0.62***<br>≥15min commute/low LTPA/ low OPA<br>HR = 0.84<br>≥15min commute/low LTPA/mod OPA<br>**HR = 0.63**<br>No commute/mod-high LTPA/ low OPA<br>**HR = 0.78**<br>No commute/mod-high LTPA/mod OPA<br>**HR = 0.62**<br>≥15min commute/mod-high LTPA/ low OPA<br>**HR = 0.67**<br>≥15min commute/mod-high LTPA/mod OPA<br>**HR = 0.66**	Active men and women who engaged in only one form of PA had a statistically significant lower all-cause mortality compared to the reference category.
Clays, 2014	Low (first tertile) vs. high (second and third tertiles)	**Model:** Cox proportional hazards<br><br>**Covariates:** age, education, BP, BMI, fitness level, occupational class, total cholesterol	N/A	Ref = low OPA/high LTPA vs.<br><br>High OPA/high LTPA vs. ref<br>HR = 1.83, 95% CI: 0.95, 3.54<br>Low OPA/low LTPA vs. ref<br>**HR = 2.07, 95% CI: 1.03, 4.19**<br>High OPA/low LTPA vs. ref<br>**HR = 2.04, 95% CI: 1.07, 3.91**	N/A	No significant interaction effects were observed among LTPA, OPA, and fitness in relation to total mortality.
Harari, 2015	None-mild OPA vs. moderate-hard OPA (Manufacturing, machine operators)	**Model:** Cox proportional hazards<br><br>**Covariates:** age, education, smoking, BMI, SES, father's country of origin, cholesterol, HDL,	N/A	Ref = moderate-hard OPA + no LTPA<br><br>Non-mild OPA + no LTPA vs. ref<br>**HR = 0.70, 95% CI: 0.57, 0.87**<br>Moderate-hard OPA + LTPA vs. ref<br>HR = 0.86, 95% CI: 0.63, 1.17<br>Non-mild OPA + LTPA vs. ref<br>**HR = 0.54, 95% CI: 0.36, 0.82**	N/A	Employees who perform moderate-hard OPA and no LTPA had the greatest risk of all-cause mortality while employees who performed none-light OPA and LTPA had the lowest risk. Does not appear to be risk of performing LTPA among moderate-hard OPA group.

First author, year	OPA groups	Model, covariates	Effect estimates			Narrative synthesis of findings
			Combined men and women	Men	Women	
		hypertension, diabetes, coffee consumption, alcohol, diet, shift work				
Hermansen, 2019	1. Sitters<br>2. Intermittent movers<br>3. Heavy labour x 2<br><br>Mostly sedentary, walking, walking and lifting, heavy manual labour	**Model:** Cox proportional hazards<br><br>**Covariates:** age, sex, smoking, BMI, also adjusted for self-reported angina pectoris, MI, cerebral insult, diabetes, anti-hypertensive medication and OPA	Ref = active LTPA + walking and lifting OPA, which showed the lowest HR.<br><br>Inactive LTPA + sedentary OPA<br>**HR = 1.29, 95% CI: 1.08, 1.55**<br>Inactive LTPA + walking OPA<br>HR = 1.15, 95% CI: 0.95, 1.40<br>Inactive LTPA + walking and lifting OPA<br>HR = 0.89, 95% CI: 0.72, 1.11<br>Inactive LTPA + heavy OPA<br>HR = 1.12, 95% CI: 0.88, 1.43<br>Moderate LTPA + sedentary OPA<br>HR = 1.14, 95% CI: 0.95, 1.36<br>Moderate LTPA + walking OPA<br>HR = 1.10, 95% CI: 0.92, 1.31<br>Moderate LTPA + walking and lifting OPA<br>HR = 1.01, 95% CI: 0.84, 1.21<br>Moderate LTPA + heavy OPA<br>HR = 1.06, 95% CI: 0.87, 1.30<br>Active LTPA + sedentary OPA<br>HR = 0.82, 95% CI: 0.66, 1.04<br>Active LTPA + walking OPA<br>HR = 0.95, 95% CI: 0.77, 1.18<br>Active LTPA + heavy OPA<br>HR = 1.17, 95% CI: 0.91, 1.49	NR	NR	Decreasing mortality with increasing LTPA was only found in the two lowest OPA categories; in higher levels of OPA, LTPA did not seem to influence mortality
Holme, 1981	Sedentary, Moderate, Intermediate, Great (not described)	**Model:** Death rates per 1,000<br><br>**Covariates:** None	N/A	Death rates are presented as the number of deaths divided by the number of men at risk per 1000.<br><br>Group 1 - Sedentary at work and sedentary at leisure time<br>Total death rate per 1000: 19.4<br><br>Group 2 - Moderate at work and sedentary at leisure time<br>Total death rate per 1000: 20.0<br><br>Group 3 - Intermediate at work and sedentary at leisure time<br>Total death rate per 1000: 24.1<br><br>Group 4 - Great at work and sedentary at leisure time<br>Total death rate per 1000: 20.3	N/A	Increasing LTPA associated with falling risk.<br><br>Greater LTPA appears to associate with lower risk reduction among higher OPA groups compared to more sedentary occupation groups.

First author, year	OPA groups	Model, covariates	Effect estimates			Narrative synthesis of findings
			Combined men and women	Men	Women	
				Group 5 - Sedentary at work and moderate at leisure time<br>Total death rate per 1000: 8.9<br><br>Group 6 - Moderate at work and moderate at leisure time<br>Total death rate per 1000: 10.0<br><br>Group 7 - Intermediate at work and moderate at leisure time<br>Total death rate per 1000: 15.2<br><br>Group 8 - Great at work and moderate at leisure time<br>Total death rate per 1000: 18.1<br><br>Group 9 - Sedentary at work and intermediate at leisure time<br>Total death rate per 1000: 7.0<br><br>Group 10 - Moderate at work and intermediate at leisure time<br>Total death rate per 1000: 5.5<br><br>Group 11 - Intermediate at work and intermediate at leisure time<br>Total death rate per 1000: 14.9<br><br>Group 12 - Great at work and intermediate at leisure time<br>Total death rate per 1000: -<br><br>Group 13 - Sedentary at work and great at leisure time<br>Total death rate per 1000: -<br><br>Group 14 - Moderate at work and great at leisure time<br>Total death rate per 1000: -<br><br>Group 15 - Intermediate at work and great at leisure time<br>Total death rate per 1000: -<br><br>Group 16 - Great at work and great at leisure time<br>Total death rate per 1000: -		
Holtermann, 2009	1. Sitters<br>2. Intermittent movers	**Model:** Cox proportional hazards	N/A	Ref = Low LTPA<br><br>**<u>Low OPA</u>**<br>Moderate LTPA vs. ref	N/A	Men who were moderately or highly physically active at leisure time, had a decreased risk of mortality in all groups,

First author, year	OPA groups	Model, covariates	Effect estimates			Narrative synthesis of findings
			Combined men and women	Men	Women	
	3. Heavy labour	**Covariates:** age, smoking, BP, BMI, treatment of diabetes or hypertension, alcohol, social class		**HR = 0.81, 95% CI: 0.66, 0.98**<br>High LTPA vs. ref<br>HR = 0.81, 95% CI: 0.60, 1.11<br><br>**Moderate OPA**<br>Moderate LTPA vs. ref<br>**HR = 0.82, 95% CI: 0.71, 0.94**<br>High LTPA vs. ref<br>**HR = 0.64, 95% CI: 0.50, 0.81**<br><br>**High OPA**<br>Moderate LTPA vs. ref<br>HR = 0.90, 95% CI: 0.71, 1.12<br>High LTPA vs. ref<br>HR = 0.84, 95% CI: 0.60, 1.18		however, not reaching statistical significance when controlling for confounding factors among men with the highest work demands. In addition, the strength of the association was somewhat smaller.
Holtermann, 2012a	1. Sitters<br>2. Intermittent movers<br>3. Heavy labour x 2	**Model:** Cox proportional hazards<br><br>**Covariates:** age, smoking, BMI, alcohol, BP including treatment, physical fitness, # of hours at work, psychosocial stress at work and leisure, social class	N/A	Among men with low physical work demands, low LTPA was associated with higher risk of all-cause mortality.<br><br>Among men with moderate physical work demands, low LTPA was significant associated with greater risk of all-cause mortality.<br><br>Among men with high physical work demands, no significant associations were found for LTPA.	N/A	
Holtermann, 2012b	1. Sitters<br>2. Intermittent movers<br>3. Heavy labour	**Model:** Cox proportional hazards<br><br>**Covariates:** age, income, smoking, BP, BMI, alcohol, diabetes, BP medication, cholesterol	N/A	Low LTPA and low OPA (ref) vs.<br>Low LTPA and moderate OPA<br>HR = 1.15, 95% CI: 0.81, 1.62<br>Low LTPA and high OPA<br>**HR = 1.56, 95% CI: 1.11, 2.18**<br><br>Moderate LTPA and low OPA (ref) vs.<br>Moderate LTPA and moderate OPA<br>**HR = 1.28, 95% CI: 1.05, 1.57**<br>Moderate LTPA and high OPA<br>**HR = 1.31, 95% CI: 1.05, 1.63**<br><br>High LTPA and low OPA (ref) vs.<br>High LTPA and moderate OPA<br>HR = 0.96, 95% CI: 0.76, 1.22<br>High LTPA and high OPA<br>HR = 1.00, 95% CI: 0.78, 1.26	Low LTPA and low OPA (ref) vs.<br>Low LTPA and moderate OPA<br>HR = 0.89, 95% CI: 0.66, 1.20<br>Low LTPA and high OPA<br>HR = 0.99, 95% CI: 0.63, 1.54<br><br>Moderate LTPA and low OPA (ref) vs.<br>Moderate LTPA and moderate OPA<br>**HR = 0.80, 95% CI: 0.68, 0.95**<br>Moderate LTPA and high OPA<br>HR = 0.92, 95% CI: 0.71, 1.18<br><br>High LTPA and low OPA (ref) vs.<br>High LTPA and moderate OPA<br>HR = 1.06, 95% CI: 0.81, 1.39<br>High LTPA and high OPA<br>HR = 1.05, 95% CI: 0.76, 1.45	Among men with low or moderate LTPA, risk of all-cause mortality was increased with higher OPA (test for trend, p<0.01 in both cases). However, among men who were highly physically active during leisure time, the risk of all cause mortality was independent of OPA level. A significant interaction between OPA and LTPA was found even after control for potential confounders.<br><br>Among women, OPA did not increase the risk of all-cause mortality in any of the LTPA groups and no significant interaction was found.

First author, year	OPA groups	Model, covariates	Effect estimates			Narrative synthesis of findings
			Combined men and women	Men	Women	
Holtermann, 2013	Men:<br>1. Sitters<br>2. Intermittent movers<br>3. Heavy labour **x 2**<br>Women:<br>1. Sitters<br>2. Intermittent movers<br>3. Heavy labour	**Model:** Cox proportional hazards<br><br>**Covariates:** age, income, smoking, BP, BMI, calendar time, alcohol, cholesterol, BP medication		Ref = Low OPA, High LTPA<br><br>Low OPA, Low LTPA vs. ref<br>**HR = 1.64, 95% CI: 1.41, 1.89**<br>Low OPA, Moderate LTPA vs. ref<br>HR = 1.13, 95% CI: 1.00-1.28<br>Moderate OPA, Low LTPA vs. ref<br>**HR = 1.60, 95% CI: 1.36, 1.89**<br>Moderate OPA, Moderate LTPA vs. ref<br>**HR = 1.19, 95% CI: 1.05, 1.35**<br>Moderate OPA, High LTPA vs. ref<br>HR = 1.01, 95% CI: 0.88, 1.16<br>High OPA, Low LTPA vs. ref<br>**HR = 1.48, 95% CI: 1.22, 1.79**<br>High OPA, Moderate LTPA vs. ref<br>**HR = 1.21, 95% CI: 1.06, 1.38**<br>High OPA, High LTPA vs. ref<br>HR = 1.07, 95% CI: 0.93, 1.23<br>Very high OPA, Low LTPA vs. ref<br>**HR = 1.65, 95% CI: 1.30, 2.09**<br>Very high OPA, Moderate LTPA vs. ref<br>**HR = 1.33, 95% CI: 1.10, 1.62**<br>Very high OPA, High LTPA vs. ref<br>HR = 1.20, 95% CI: 1.00, 1.45	Ref = Low OPA, High LTPA<br><br>Low OPA, Low LTPA vs. ref<br>**HR = 1.58, 95% CI: 1.34, 1.87**<br>Low OPA, Moderate LTPA vs. ref<br>**HR = 1.22, 95% CI: 1.05, 1.41**<br>Moderate OPA, Low LTPA vs. ref<br>**HR = 1.51, 95% CI: 1.29, 1.78**<br>Moderate OPA, Moderate LTPA vs. ref<br>HR = 1.08, 95% CI: 0.94, 1.24<br>Moderate OPA, High LTPA vs. ref<br>HR = 1.04, 95% CI: 0.89, 1.21<br>High OPA, Low LTPA vs. ref<br>**HR = 1.52, 95% CI: 1.23, 1.89**<br>High OPA, Moderate LTPA vs. ref<br>**HR = 1.24, 95% CI: 1.06, 1.46**<br>High OPA, High LTPA vs. ref<br>HR = 1.07, 95% CI: 0.89, 1.29	Corresponding with the consistent risk reductions from higher levels of LTPA in all groups of OPA, no statistical interaction between LTPA and OPA in either men or women for and all-cause mortality (P=0.47) were found.<br><br>Hence, the effect of LTPA on all-cause mortality seems to be independent of the level of OPA.
Petersen, 2012	Heavy lifting vs. no heavy lifting	**Model:** Cox proportional hazards<br><br>**Covariates:** age, education, smoking, alcohol, stress, OPA	NR	Ref = High LTPA, no occupational lifting<br><br>High LTPA, occupational lifting vs ref.<br>HR = 0.82, 95% CI: 0.45, 1.50<br>Low LTPA, no occupational lifting vs ref.<br>HR = 1.28, 95% CI: 0.90, 1.83<br>Low LTPA, occupational lifting vs ref.<br>HR = 1.33, 95% CI: 0.87, 2.04	Ref = High LTPA, no occupational lifting<br><br>High LTPA, occupational lifting vs ref.<br>HR = 1.76, 95% CI: 0.62, 4.98<br>Low LTPA, no occupational lifting vs ref.<br>HR = 1.44, 95% CI: 0.77, 2.69<br>Low LTPA, occupational lifting vs ref.<br>HR = 1.25, 95% CI: 0.59, 2.67	For men, low LTPA was associated with increased risk of all-cause mortality. Effects were stronger among those who did a lot of occupational lifting, compared to those without.<br><br>Among women the associations were the other way around
Stamatakis, 2013	Sitting vs. non-sitting occupations	**Model:** Cox proportional hazards<br><br>**Covariates:** age, education, smoking, alcohol, general health, CVD at baseline, cancer at baseline, occupational class, WC, non-OPA, psychological health, social class		No differences in association of sitting vs. non-sitting occupations by LTPA level.<br><br>Ref = Low LTPA, sitting occupation vs. High LTPA/non-sitting occupation<br>**HR = 0.74, 95% CI: 0.56, 0.97** (figure includes other comparisons)<br><br>Risk lowest among high LTPA groups and was similar between sitting and non-sitting occupations.	Among women, evidence for an interaction b/w OPA and LTPA for all-cause mortality (**p = 0.011**).<br><br>Ref = Low LTPA, sitting occupation vs. High LTPA/non-sitting occupation<br>**HR = 0.47, 95% CI: 0.32, 0.70** (figure includes other comparisons)<br><br>Among women, sitting occupations linked to increased risk of all-cause regardless of LTPA (Figure 1).	High LTPA was associated with a reduced risk of all-cause mortality. Effects were stronger among those with non-sitting occupations. In women, effects were weaker.<br><br>Greatest protection among those with highest LTPA and non-sitting occupation.

Bolded data = statistically significant ($p<0.05$). BMI – body mass index, BP – blood pressure, CES-D – Centre for Epidemiological Studies Depression questionnaire, CI – confidence interval, CVD – cardiovascular disease, DASH – Disability of the Arm, Shoulder and Hand, HADS – Hospital Anxiety and

Depression Scale, HDL – high-density lipoprotein, HR – hazards ratio, IHD – ischemic heart disease, LPA – light intensity physical activity, LTPA – leisure-time physical activity, M – mean, MD – mean difference, mod. – moderate, MI – myocardial infarction, MPA – moderate-intensity physical activity, MSKP – musculoskeletal pain, MVPA – moderate-to-vigorous intensity physical activity, NR – not reported, OPA – occupational physical activity, OR – odds ratio, PA – physical activity, ref – referent group, RR – relative risk, SBP – systolic blood pressure, VAS – visual analogue scale, VPA – vigorous intensity physical activity, WC – waist circumference

Supplemental table 5. Study characteristics table for studies examining effects of OPA and LTSB by health outcome

First author, year	Country, cohort	Population description	OPA		Age mean (SD), range in years	Study design	Sample size analyzed			Length of follow-up	Sedentary time		Outcome assessment
			Classification method	OPA groups			Total N	Men N (%)	Women N (%)		Assessment/ intervention	Sedentary groups	
**All-cause mortality**													
Pulsford, 2015 [55]	United Kingdom, Whitehall II Study	Office workers (Civil servants including clerical and office support, middle-ranking executive and senior administrative grades)	Occupation title	Sitters	35-55	PCS	5,132	NR	NR	Mean = 15.7 years	Study-specific, Phase 5 SR sitting questionnaire	Total leisure sitting time: Ref = 0 to <15 h/week, ≥15 to <18 h/week, ≥18 to <26 h/week, ≥26 h/week<br><br>TV viewing: Ref = 0 to < 8 h/week vs. ≥8 to <15 h/week, ≥15 to <16 h/week, ≥16 h/week	Death registry

NR – not reported, PCS – prospective cohort study, Ref – referent group, SR – self-report

Supplemental table 6. Study findings table for studies examining effects of OPA and LTSB and all-cause morality

First author, year	OPA groups	Model, covariates	Effect estimates			Narrative synthesis of findings
			Combined men and women	Men	Women	
**All-cause mortality**						
Pulsford, 2015	Sitters (office workers)	**Model:** cox proportional hazards<br><br>**Covariates:** age, sex, smoking, BMI, ethnicity, alcohol, employment grade, diet, physical function, MVPA	Ref = leisure sitting 0 to <15 h/week<br>≥15 to <18 h/week<br>HR = 1.06, 95% CI: 0.79, 1.41<br>≥18 to <26 h/week<br>HR = 1.03, 95% CI: 0.78, 1.36<br>≥26 h/week<br>HR = 1.29, 95% CI: 0.94, 1.67<br>P_{trend} = 0.18<br><br>Ref = TV viewing 0 to < 8 h/week<br>≥8 to <15 h/week<br>HR = 1.00, 95% CI: 0.66, 1.51<br>≥15 to <16 h/week<br>HR = 1.01, 95% CI: 0.68, 1.49<br>≥16 h/week<br>HR = 1.13, 95% CI: 0.77, 1.68<br>P_{trend} = 0.80	NR	NR	Among sedentary workers, those who sit most during leisure time had the highest risk for all-cause morality, though not statistically significant.<br><br>Being in the group that watched the most television was not statistically associated with higher all-cause mortality.

BMI – body mass index, HR – hazard ratio, MVPA – moderate-to-vigorous intensity physical activity, Ref = referent group
